# Single Electron Transfer to Diazomethane–Borane Adducts Prompts C−H Bond Activations

**DOI:** 10.1002/anie.201912338

**Published:** 2019-10-30

**Authors:** Levy L. Cao, Jiliang Zhou, Zheng‐Wang Qu, Douglas W. Stephan

**Affiliations:** ^1^ Department of Chemistry University of Toronto 80 St. George St. Toronto Ontario M5S3H6 Canada; ^2^ Mulliken Center for Theoretical Chemistry Institut für Physikalische und Theoretische Chemie Rheinische Friedrich-Wilhelms-Universität Bonn Beringstrasse 4 53115 Bonn Germany

**Keywords:** borane, DFT, diazomethane, one-electron reduction, radical anions

## Abstract

While (Ph_2_CN_2_)B(C_6_F_5_)_3_ is unstable, single electron transfer from Cp*_2_Co affords the isolation of stable products [Cp*_2_Co][Ph_2_CNNHB(C_6_F_5_)_3_] **1** and [Cp*Co(C_5_Me_4_CH_2_B(C_6_F_5_)_3_)] **2**. The analogous combination of Ph_2_CN_2_ and BPh_3_ showed no evidence of adduct formation and yet single electron transfer from Cp*_2_Cr affords the species [Cp*_2_Cr][PhC(C_6_H_4_)NNBPh_3_] **3** and [Cp*_2_Cr][Ph_2_CNNHBPh_3_] **4**. Computations showed both reactions proceed via transient radical anions of the diphenyldiazomethane–borane adducts to effect C−H bond activations.

## Graphical abstract

**Single electron transfer** to diphenyldiazomethane–borane adducts generates transient radical anions that effect C−H bond activations. Such reductions provide a strategy to stabilize weak and even undetectable donor–acceptor adducts.

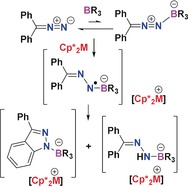

The activation of small molecules has been a major driver of organometallic chemistry over the last 60 years. Such efforts have spawned great interest and important developments. In recent years, such inquiries have begun to permeate main group chemistry. One avenue of main group chemistry exploited for the activation of small molecules has been frustrated Lewis pair (FLP) chemistry.^1^ While this initially emerged from the finding of the heterolytic activation of H_2_ by combinations of Lewis acids and bases,^2^ subsequent efforts demonstrated reactivity of FLPs with a wide range of small molecules.^3^ Noticeably absence from these investigations have been studies involving dinitrogen.

Organometallic chemists have studied metal–N_2_ systems since the seminal report of A. D. Allen^4^ who described the first transition metal–dinitrogen complex. Over the past 50 years numerous advances have emerged from the luminaries of organometallic chemistry including Schrock,^5^ Cummins,^6^ Peters,^7^ Fryzuk,^8^ Evans,^9^ Gambarotta,^10^ Nishibiashi,^11^ Holland,^12^ Chatt,^13^ and Liddle^14^ among others.^15^ Avenues to metal‐mediated N_2_ chemistry have typically involved stoichiometric reductants.8d More recently, in 2017 the Szymczak^16^ and Simonneau^17^ groups demonstrated the utility of a Lewis acidic borane in promoting reactivity of metal‐bound N_2_ fragments, effecting protonation, borylation and silylation of N_2_ bound between the metal and boron.

Main group interactions with N_2_ have drawn much less attention. A number of computational studies have addressed the interactions of N_2_ with Lewis acids, while the species (N_2_)BF_3_ was spectroscopically characterized upon generation by supersonic expansion at 600 torr and 170 K.^18^ The compound Ph_3_PNNPPh_3_ 19 although not derived from N_2_, was controversially described as N_2_ stabilized by two phosphine donors.^20^ However, in a truly seminal finding, Braunschweig et al.^21^ described the first metal‐free capture of N_2_ using a cAAC‐stabilized borylene (cAAC: cyclic (alkyl)(amino) carbene).

In our own efforts towards main group‐N_2_ chemistry, we initiated studies of diazomethanes which liberate N_2_. Such systems may provide insight for the design of main group‐N_2_ systems.^22^ In 2012, we reported the insertion of diazomethanes into B−C bonds of electrophilic boranes with the liberation of N_2_ (Scheme 1).^23^ Such insertions were recently exploited in organic synthesis by Melen et al.^24^ In recent work,^25^ we showed that the sterically‐encumbered diazomethane, Ph_2_CN_2_, does not insert but rather forms a highly reactive, yet isolable borane‐adduct, (Ph_2_CN_2_)B(C_6_F_5_)_3_. Moreover, we also showed weak Lewis acid–base adducts were stabilized by stoichiometric reduction.^26^ This notion was also exploited by the Erker group in the isolation of Lewis acid stabilized radicals.^27^ Herein, we probe the impact of reduction on the reactivity of the unstable (Ph_2_CN_2_)B(C_6_F_5_)_3_, demonstrating that single electron transfer to diazomethane‐borane adducts stabilizes weak B⋅⋅⋅N interactions providing reactive transient radicals which effect C−H bond activation.

**Scheme 1:**
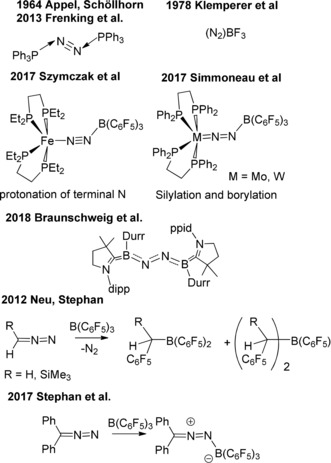
Interactions of main group systems with N_2_‐fragments.

A 1:1 combination of diphenyldiazomethane (Ph_2_CN_2_) with B(C_6_F_5_)_3_ in chlorobenzene was stirred at −35 °C. Addition of an equal molar amount of Cp*_2_Co immediately gave a yellow solution. The crude ^19^F NMR spectrum showed two sets of resonances at −134.0, −163.9, and −167.3 ppm and −130.2, −161.9, −162.7 ppm, attributable to inequivalent C_6_F_5_ rings. The ^11^B NMR spectrum showed two resonances at −7.6 and −13.0 ppm attributable to two tetra‐coordinated boron species, **1** and **2**, respectively. ^1^H NMR data showed resonances at 6.61 and 2.43 ppm attributable to NH and CH_2_ fragments. Fractional recrystallization permitted formulation of the two products **1** and **2** by X‐ray crystallographic analysis. Compound **1** was found to be the salt [Cp*_2_Co][Ph_2_CNNHB(C_6_F_5_)_3_] (Figure 1 a). While the cation was unexceptional, the diazoborate anion was derived from the interaction of the hydrazide bound to borane. The B−N(H) bond length in **1** is 1.539(7) Å, while the N−N and N−C bonds lengths is 1.342(5) and 1.303(7) Å, respectively. The B−N−N angle was determined to be 118.2(3)° while the C−N−N angle is 120.9(4)°. The second isolated product was confirmed to be [Cp*Co(C_5_Me_4_CH_2_B(C_6_F_5_)_3_)] **2** (Figure 1 b). In this species, one of the hydrogen atoms in one of the Cp* methyl groups has been replaced by borane, affording the zwitterionic Co^III^‐borate **2**, with a methylene–boron B−C bond length of 1.66(1) Å.

**Figure 1:**
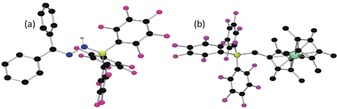
POV‐Ray depiction of the anion of a) **1** and b) **2**. The cation and hydrogen atoms (except NH) are omitted for clarity. C: black, N: blue, F: pink, B: yellow‐green, Co: green, and H: grey.

Collectively, the identification of **1** and **2** is consistent with two possible reaction mechanisms involving single electron transfer from a Co^II^ center to either B(C_6_F_5_)_3_ 28 or the diazomethane adduct of the borane, (Ph_2_CN_2_)B(C_6_F_5_)_3_.^25^ It is noteworthy that although C−H activation by the radical [B(C_6_F_5_)_3_)]^.−^,^29^ is expected to give the anion [HB(C_6_F_5_)_3_]^−^, independent combination of diazomethane with [HB(C_6_F_5_)_3_]^−^ showed no reaction. This supports the view that compound **1** is formed through hydrogen atom abstraction from Cp*_2_Co by the transient diazomethane‐borane adduct radical anion [Ph_2_CN_2_B(C_6_F_5_)_3_]^.−^ (Scheme 2), consistent with the overall reaction ratio of diazomethane:Cp*_2_Co:B(C_6_F_5_)_3_ of 1:2:2.

**Scheme 2:**
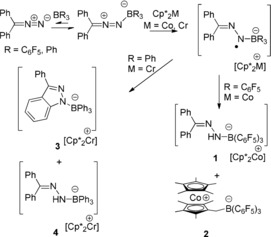
Reactions of Ph_2_CN_2_ with B(C_6_F_5_)_3_ and Cp*_2_Co, and with BPh_3_ and Cp*_2_Cr.

Ph_2_CN_2_ was combined with BPh_3_ in chlorobenzene at −35 °C. Monitoring the solution by multinuclear NMR spectroscopy revealed no evidence of adduct formation. This is consistent with the poor Lewis basicity of the diazomethane and the weaker Lewis acidity of the BPh_3_ in comparison to B(C_6_F_5_)_3_, in line with the computed free energies (see Supporting Information). Addition of Cp*_2_Cr to a mixture of BPh_3_ and diazomethane at −35 °C generated an orange solution. The ^11^B NMR spectrum showed resonances at 23.0 and −3.5 ppm consistent with the formation of two products, **3** and **4** which were isolated by fractional crystallization. An X‐ray diffraction study revealed species **3** to be [Cp*_2_Cr][PhC(C_6_H_4_)NNBPh_3_] (Scheme 2, Figure 2 a). While the cation was typical, the anion of **3** was shown to be a borate with a substituent derived from the cyclization of the N_2_ fragment onto the *ortho* position of one of the aryl rings on the diazomethane carbon. The resulting five membered ring which is fused to the aryl ring is 1,3‐disubstituted with and phenyl ring on carbon and BPh_3_ bound to nitrogen. The resulting N−B bond is 1.566(6) Å, while the N−N and new N−C bond distances are determined to be 1.312(5) and 1.351(5) Å. The second product **4** was also characterized crystallographically revealing its formulation as [Cp*_2_Cr][Ph_2_CNNHBPh_3_] (Scheme 2, Figure 2 b). The B−N and N−N distances in the anion of **4** were determined to be 1.562(4) Å and 1.322(3) Å, respectively.

**Figure 2:**
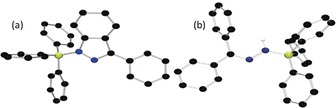
POV‐Ray depiction of the anion of a) **3** and b) **4**. The cation and hydrogen atoms (except NH) are omitted for clarity. C: black, N: blue, and B: yellow‐green.

In contrast, the reaction of 9‐diazofluorene ((C_12_H_8_)CN_2_) with B(C_6_F_5_)_3_ did not form an adduct but led to loss of N_2_ (Scheme 3) and the formation of the carboboration product (C_12_H_8_)C(C_6_F_5_)(B(C_6_F_5_)_2_) as confirmed spectroscopically and crystallographically (see Supporting Information). This is consistent with observations seen for less sterically encumbered diazomethanes.^23^, ^30^ However, monitoring the reaction of (C_12_H_8_)CN_2_ with BPh_3_ and Cp*_2_Cr by ^11^B NMR spectroscopy revealed the generation of three products as evidenced by the resonances at 26.2, 2.4, and −1.7 ppm. The peaks at −1.7 and 2.4 ppm were unambiguously assigned to [Cp*_2_Cr][C_12_H_8_CNNHBPh_3_] **5** and [Cp*Cr(C_5_Me_4_CH_2_BPh_3_)] **7** by NMR and crystallographic methods (Figure 3, see Supporting Information). The remaining resonance at 26.2 ppm, was attributed to the species [Cp*_2_Cr][C_13_H_7_N_2_BPh_3_] **6** by analogy to **3**. These results suggest that the electron transfer to the weak adducts gives a transient radical [(C_12_H_8_)CN_2_BPh_3_]^.−^, which reacts further through competitive pathways involving either intramolecular cyclization or intermolecular H‐atom abstraction.

**Figure 3:**
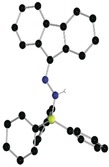
POV‐Ray depiction of the anion of **5**. The cation and hydrogen atoms (except NH) are omitted for clarity. C: black, N: blue, and B: yellow‐green.

**Scheme 3:**
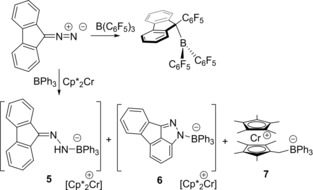
Reactions of (C_12_H_8_)CN_2_ with BPh_3_ and Cp*_2_Cr.

The mechanism of these reactions were probed by density functional theory (DFT) calculations at the PW6B95‐D3/def2‐QZVP + COSMO‐RS// TPSS‐D3/def2‐TZVP + COSMO level of theory in chlorobenzene solution.^31^ The reaction of Ph_2_CN_2_ with BPh_3_ and Cp*_2_Cr is initiated by single electron transfer from Cp*_2_Cr to the unstable Ph_2_CN_2_⋅BPh_3_ adduct (Figure 4) affording the radical anion [Ph_2_CNNHBPh_3_]^.−^ **INT1** (spin on N next to B: 0.53*e*) is 4.3 kcal mol^−1^ endergonic. Alternative pathways involving electron transfers to separated BPh_3_ and Ph_2_CN_2_ species (14.8 and 8.9 kcal mol^−1^) are significantly less favorable. Similarly, further electron transfer to **INT1** is unlikely (12.2 kcal mol^−1^ endergonic). The N‐centered radical **INT1** may then add intramolecularly to the *ortho* position of a phenyl ring (via transition structure **TS1**) to give **INT2** affording delocalization of the spin onto the ring. From here, a highly exergonic H‐transfer to another Ph_2_CN_2_ molecule (via **TS2**) gives the anion of **3** and the neutral N‐radical (Ph_2_CNNH)^.^ (spin on N next to H: 0.54*e*) with a moderate overall barrier of 23.5 kcal mol^−1^. The latter radical is readily reduced by Cp*_2_Cr through electron transfer and trapped by BPh_3_ giving the anion of **4** (−66.4 kcal mol^−1^).

**Figure 4:**
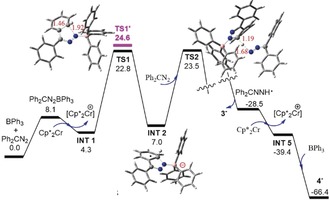
DFT‐computed free energy profile (kcal mol^−1^, at 298 K and 1 mol L^−1^ reference concentration) for the formation of anion of **3** and **4**. Selected bond lengths are given in angstroms while selected C: grey, H: white, N: blue, and B: pink.

For the reaction of Ph_2_CN_2_ with the stronger Lewis acid B(C_6_F_5_)_3_ and the more reductive Cp*_2_Co,^32^ electron transfer from Cp*_2_Co to the reversible adduct (Ph_2_CNN)B(C_6_F_5_)_3_ is −15.3 kcal mol^−1^ exergonic affording the radical anion [Ph_2_CN_2_B(C_6_F_5_)_3_]^⋅−^. This intermediate is computed to effect H‐atom from one methyl group of Cp*_2_Co over a barrier of 16.6 kcal mol^−1^ to form the stable anion of **1** (see Supporting Information). The alternative intramolecular pathway, analogous to that above encounters a higher overall barrier of 23.3 kcal mol^−1^ (see Supporting Information).

To garner further support for the computed mechanism, efforts to observe the transient radical adducts in the reactions were undertaken, but were unsuccessful. However, monitoring the reaction of (C_12_H_8_)CN_2_, Cp*_2_Fe and Al(C_6_F_5_)_3_ in C_6_H_5_Cl at room temperature by EPR spectroscopy revealed a pentet resonance at g(iso)=2.0039, with (^14^N) hyperfine couplings of 3.70 G and 3.58 G. This signal was similar to the related N‐based radicals^33^ and was attributed to the radical species [Ph_2_CN_2_Al(C_6_F_5_)_3_]^.−^. This signal slowly degrades at room temperature over 5 h, leaving a broad resonance attributed to an organic radical (see Supporting Information). Subsequent addition of Ph_3_SnH generated a mixture of products, from which [Cp*_2_Fe][(C_12_H_8_)CNNHAl(C_6_F_5_)_3_] **8** was identified by NMR spectroscopy while single crystals of [Cp*_2_Fe][(C_12_H_8_)CHAl(C_6_F_5_)_3_] **9** were obtained from the reaction mixture (Figure 5). Compound **8** was independently prepared and crystallographically characterized from the reaction of (C_12_H_8_)CNNH_2_, Cp*_2_Fe, and Al(C_6_F_5_)_3_. The formulations of **8** and **9** are consistent with the generation of the spectroscopically observed radical anions [(C_12_H_8_)CN_2_Al(C_6_F_5_)_3_]^.−^ and [(C_12_H_8_)CAl(C_6_F_5_)_3_]^.−^ (Scheme 4).

**Figure 5:**
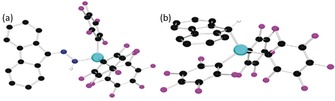
POV‐Ray depiction of the anion of a) **8** and b) **9**. The cation and hydrogen atoms (except the NH and the Al‐bound CH) are omitted for clarity. C: black, N: blue, Al: Cyan, and F: pink.

**Scheme 4:**
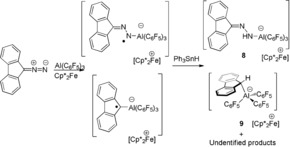
Reactions of (C_12_H_8_)CN_2_, Al(C_6_F_5_)_3_ and Cp*_2_Fe with Ph_3_SnH.

In conclusion, we have demonstrated that single electron transfer to unstable diazomethane‐borane adducts, accesses reactive radical anions that effect H‐atom abstraction from C−H bonds. The resulting anionic species are significantly more stable than the corresponding neutral adducts, suggesting that in situ reductions may be a useful strategy to infer the presence of weakly bound adducts. We suggest that this strategy could be exploited in developing main group‐N_2_ chemistry. At the same time, the potential utility of main group radical anions in C−H bond homolysis offers an interesting prospect for C−H functionalization.

## Conflict of interest

The authors declare no conflict of interest.

## Supporting information

As a service to our authors and readers, this journal provides supporting information supplied by the authors. Such materials are peer reviewed and may be re‐organized for online delivery, but are not copy‐edited or typeset. Technical support issues arising from supporting information (other than missing files) should be addressed to the authors.

Supplementary
