# Supplementary material for: Single Electron Transfer to Diazomethane–Borane Adducts Prompts C−H Bond Activations
Source: Angew Chem Int Ed Engl. 2019 Oct 30;58(51):18487–91. doi: 10.1002/anie.201912338 (PMC6972512; doi:10.1002/anie.201912338)
Supplement: Supplementary file 1 — Supplementary [file ANIE-58-18487-s001.pdf]

Angewandte Chemie
Eine Zeitschrift der Gesellschaft Deutscher Chemiker

Supporting Information

Single Electron Transfer to Diazomethane–Borane Adducts Prompts C–H Bond Activations

Levy L. Cao, Jiliang Zhou, Zheng-Wang Qu, and Douglas W. Stephan**

anie_201912338_sm_miscellaneous_information.pdf

Supporting information

Contents

1. Syntheses... 3

General Considerations.. 3

Synthesis of [Cp*$_2$Co][(Ph$_2$CNNHB(C$_6$F$_5$)$_3$] (**1**) and [Cp*CoC$_5$Me$_4$CH$_2$B(C$_6$F$_5$)$_3$] (**2**) 4

Synthesis of [Cp*$_2$Cr][(PhC(C$_6$H$_4$)NNBPh$_3$] (**3**) and [Cp*$_2$Cr][Ph$_2$CNNHBPh$_3$] (**4**).............. 10

Synthesis of (C$_{12}$H$_8$)C(C$_6$F$_5$)(B(C$_6$F$_5$)$_2$).. 13

Generation of [Cp*$_2$Cr][C$_{12}$H$_8$CNNHBPh$_3$] (**5**), [Cp*$_2$Cr][C$_{13}$H$_7$N$_2$(BPh$_3$)] (**6**) and [Cp*Cr(C$_5$Me$_4$CH$_2$BPh$_3$)] (**7**) 16

Synthesis of [Cp*$_2$Fe][C$_{12}$H$_8$CNNH(Al(C$_6$F$_5$)$_3$)] (**8**)... 18

Generation of **8** and [Cp*$_2$Fe][(C$_{12}$H$_8$)CH(Al(C$_6$F$_5$)$_3$)] (**9**).. 21

2. X-ray Crystallography 23

3. Computational Chemistry 27

4. References... 51

Lists of Figures

Figure S1. $^{11}B\{^1H\}$ NMR of the crude reaction for **1** and **2**. 4
Figure S2. $^{19}F\{^1H\}$ NMR of the crude reaction for **1** and **2**. 5
Figure S3. ^1H NMR of **1** 5
Figure S4. $^{13}C\{^1H\}$ NMR of **1** 6
Figure S5. $^{19}F\{^1H\}$ NMR of **1** 6
Figure S6. $^{11}B\{^1H\}$ NMR of **1** 7
Figure S7. ^1H NMR of **2** 8
Figure S8. $^{13}C\{^1H\}$ NMR of **2** 8
Figure S9. $^{19}F\{^1H\}$ NMR of **2** 9
Figure S10. $^{11}B\{^1H\}$ NMR of **2** 10
Figure S11. ^1H NMR of **3** 11
Figure S12. $^{13}C\{^1H\}$ NMR of **3** 11
Figure S13. $^{11}B\{^1H\}$ NMR of **3** 11
Figure S14. ^1H NMR of **4** 12
Figure S15. $^{13}C\{^1H\}$ NMR of **4** 13
Figure S16. $^{11}B\{^1H\}$ NMR of **4** 13
Figure S17. POV-Ray depiction of $(C_{12}H_8)C(C_6F_5)(B(C_6F_5)_2)$. The hydrogen atoms are omitted for clarity. C: black, F: pink B: yellow-green. 14
Figure S18. ^1H NMR of $(C_{12}H_8)C(C_6F_5)(B(C_6F_5)_2)$. 14
Figure S19. $^{13}C\{^1H\}$ NMR of $(C_{12}H_8)C(C_6F_5)(B(C_6F_5)_2)$. 15
Figure S20. ^{19}F NMR of $(C_{12}H_8)C(C_6F_5)(B(C_6F_5)_2)$. 15
Figure S21. $^{11}B\{^1H\}$ NMR of $(C_{12}H_8)C(C_6F_5)(B(C_6F_5)_2)$. 16
Figure S22. $^{11}B\{^1H\}$ NMR of the crude reaction for **5**, **6**, and **7**. 17
Figure S23. $^{13}C\{^1H\}$ NMR of **5**. 17
Figure S24. $^{11}B\{^1H\}$ NMR of **5**. 18
Figure S25. POV-Ray depiction of **6**. The hydrogen atoms are omitted for clarity. C: black, Cr: green B: yellow-green. 18
Figure S26. ^1H NMR of **8**. 19
Figure S27. $^{13}C\{^1H\}$ NMR of **8**. 20
Figure S28. ^{19}F NMR of **8**. 20
Figure S29. Experimental and simulation EPR spectra of the reaction crude for generation of **8** and **9**. 21
Figure S30. Stacked ^{19}F NMR spectra for the crude reaction comparing with **8**. 22
Figure S31. The DFT-computed reaction free energy paths in PhCl solution (in kcal/mol, at 298 K and 1 mol/L reference concentration) for the reactions of Ph_2CNN with (A) $B(C_6F_5)_3$ and $Cp*_2Co^·$, and (B) BPh_3 and triplet $Cp*_2Cr$, computed at the PW6B95-D3/def2-QZVP + COSMO-RS // TPSS-D3/def2-TZVP + COSMO level of theory. More detailed energies and brief description of potential reactions are given in Tables S1. Selected bond lengths are given in Å; crucial H, C, N and B atoms are highlighted as white, grey, blue and pink balls, respectively. 28

1. Syntheses

General Considerations: All manipulations were performed under an atmosphere of dry, oxygen-free N_2 by means of standard Schlenk or glovebox techniques (Innovative Technology glovebox equipped with a -35 °C freezer). Toluene (tol) and pentane were collected from a Grubbs-type column system manufactured by Innovative Technology, and stored over 4 Å molecular sieves. Molecular sieves, type 4 Å (pellets, 3.2 mm diameter) purchased from Sigma Aldrich were activated prior to usage by iteratively heating with 1050 W Haier microwave for 5 minutes and cooling under vacuum. The process was repeated until no further moisture was released upon heating. Benzene-d_6 (C_6D_6), bromobenzene-d_5 (C_6D_5Br) and chloroform-d ($CDCl_3$), purchased from Cambridge Isotope Laboratories, were degassed and stored over 4 Å molecular sieves in the glovebox for at least 8 h prior to use. Chlorobenzene, dichloromethane-d_2 (CD_2Cl_2) and Difluorobenzene (DFB) and benzene were dried by stirring over CaH_2 for several days followed by distillation. Unless otherwise mentioned, chemicals were purchased from Sigma Aldrich, Strem Chemical or TCI. Ph_2CN_2 and $(C_{12}H_8)CN_2$ prepared using literature methods.[1] NMR Spectra were recorded on a Bruker Avance III 400 MHz, Agilent DD2 500 MHz or an Agilent DD2 600 MHz, spectrometer and spectra were referenced to residual solvents of CD_2Cl_2 (^1H = 5.32 ppm and ^{13}C = 53.84 ppm) , C_6D_6 (^1H = 7.16 ppm and ^{13}C = 128.06 ppm) and $CDCl_3$ (^1H = 7.26 ppm and ^{13}C = 77.16 ppm), or externally (^{19}F: $CFCl_3$ and ^{11}B: $(Et_2O)BF_3$). Chemical shifts (δ) are reported in ppm. In some instances, signal and/or coupling assignments were derived from 2D NMR experiments. Mass spectrometry using a JEOL Accu TOF modelJMS-T1000LC (with DART ion source) were attempted for compound **1 - 4**, **5**, and **8,** however, as all the compounds are highly sensitive to moisture, none satisfied results were obtained, Elemental analysis (C, H, N) were also attempted for the compounds, but results show low on carbon content, while providing satisfactory H and N values. This phenomenon is attributed to the formation of boron carbides during combustion and has been previously reported for other boron compounds.

Synthesis of $[Cp^*{}_2Co][(Ph_2CNNHB(C_6F_5)_3]$ (1) and $[Cp^*CoC_5Me_4CH_2B(C_6F_5)_3]$ (2)

A solution of Ph_2CN_2 (11.3 mg, 0.058 mmol) in 2 mL of pentane was stirred in a vial at -35℃, while $B(C_6F_5)_3$ (59.3 mg, 0.116 mmol) and $Cp^*{}_2Co$ (38.2 mg, 0.116 mmol) were added sequentially, resulting a yellow solid immediately crashed out from the solution. The solvent was decanted carefully with a pipette, and the resulting solid was washed with 3 × 2 mL of pentane. Clean compound **2** was extracted from the crude product with 3 × 2 mL of benzene, and dried as yellow powder(16.5 mg Yield 34%), while the residue was dried to give the clean compound **1** as yellow powder (34.0 mg, Yield 57%).

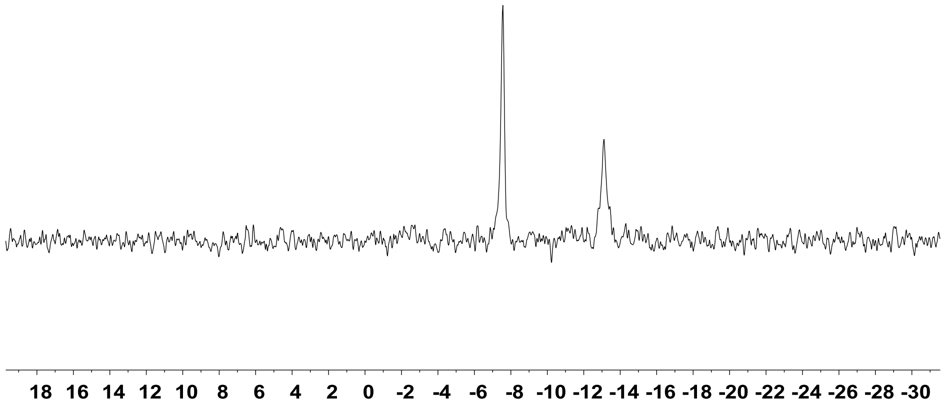

Figure S1. $^{11}B\{^1H\}$ NMR of the crude reaction for **1** and **2**.

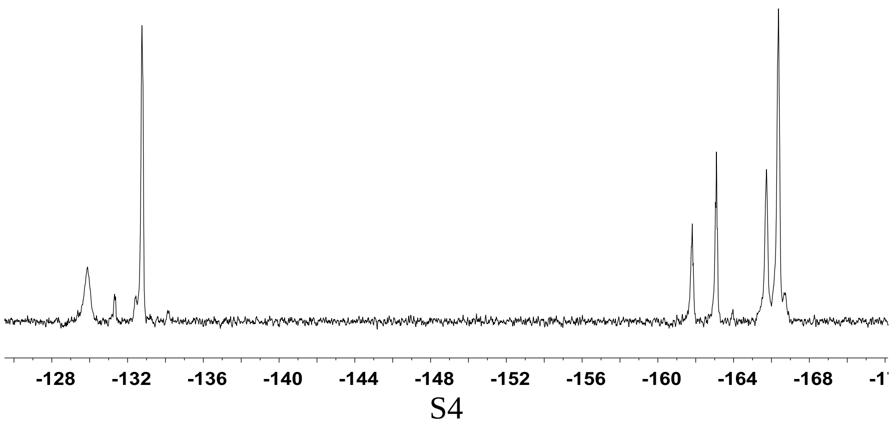

Figure S2. $^{19}F\{^1H\}$ NMR of the crude reaction for **1** and **2**.

Single crystals for X-ray studies were obtained from slow evaporation of a pentane into a saturated chlorobenzene solution for **1** and a saturated benzene solution for **2** at room temperature to give yellow crystals.

For **1**:

^{1}H NMR (500MHz, CD_2Cl_2, 298 K, pentane omitted): δ 7.52 (t, 2H, -C_6H_5, $^3J_{HH}$ = 7.5 Hz), 7.40 (t, 1H, -C_6H_5, $^3J_{HH}$ = 7.5 Hz), 7.35 – 7.28 (m, 2H, -C_6H_5), 7.14 – 7.01 (m, 4H, -C_6H_5), 6.94 (t, 1H, -C_6H_5, $^3J_{HH}$ = 6.7 Hz), 6.61(s, br, 1H, -N*H*-), 1.70 (s, 30H, -C*H*$_3$). **^{13}C{^{1}H} NMR** (128 MHz, CD_2Cl_2, 298 K): δ 142.1, 135.9, 130.8, 130.2, 129.4, 129.3, 128.9, 128.6, 127.9, 127.7, 126.9, 124.7, 124.6, 94.4 (*C*-CH_3), 8.2 (-*C*H_3). **^{19}F{^{1}H} NMR** (C_6D_5Br, 377 MHz, 298 K): δ -134.0 (d, 6F, *o*–C_6F_5, $^3J_{FF}$ = 24 Hz), -163.9 (t, 3F, *p*–C_6F_5, $^3J_{FF}$ = 21 Hz)), -167.3 (t, 6F, *m*–C_6F_5, $^3J_{FF}$ = 22 Hz). **^{11}B{^{1}H} NMR** (C_6D_5Br, 128 MHz, 298 K): δ -7.6.

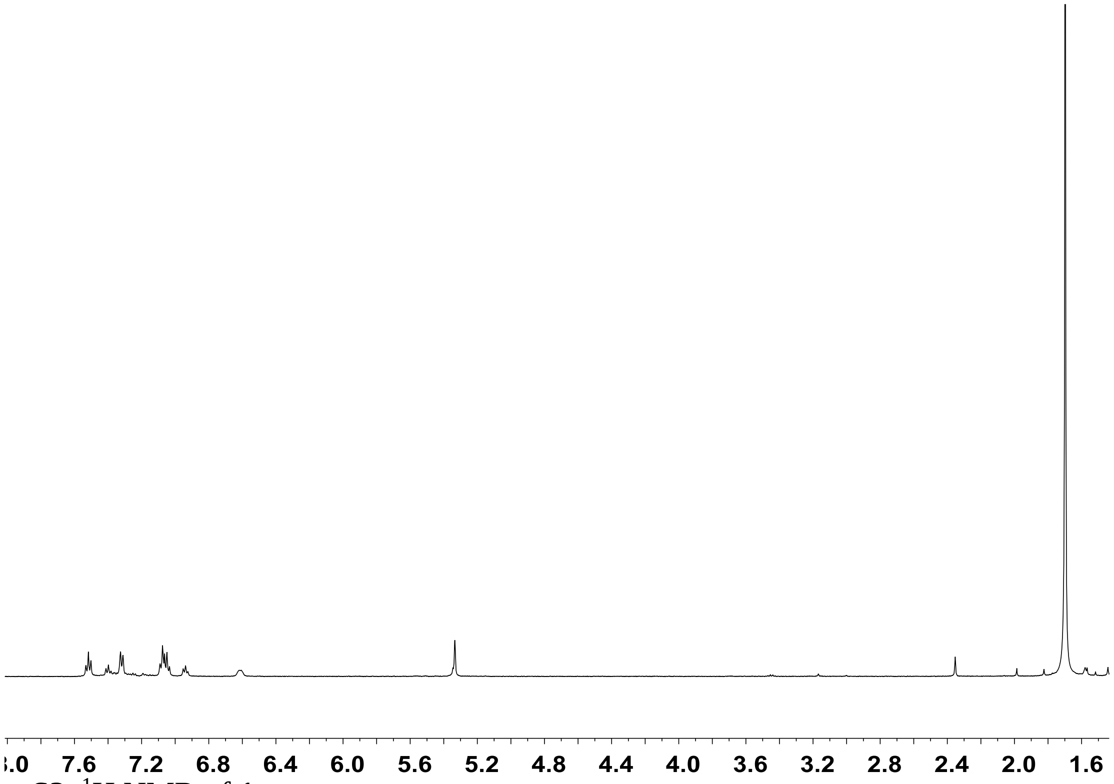

Figure S3. ^{1}H NMR of **1**

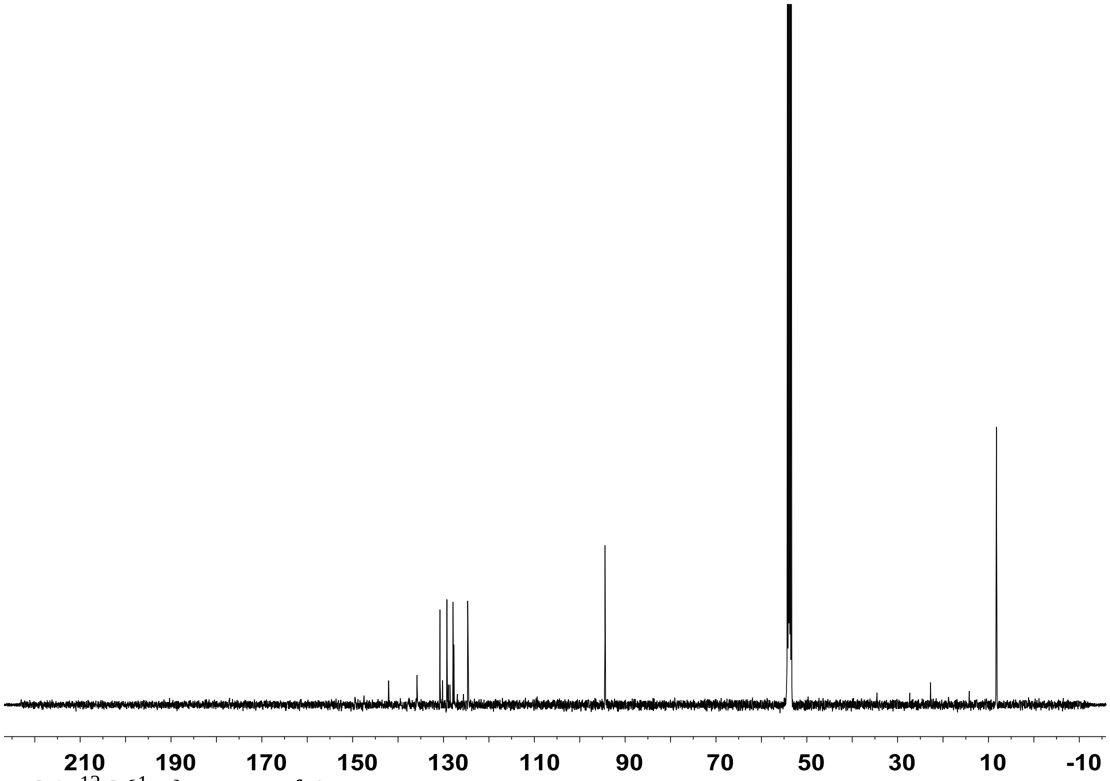

Figure S4. $^{13}C\{^1H\}$ NMR of **1**

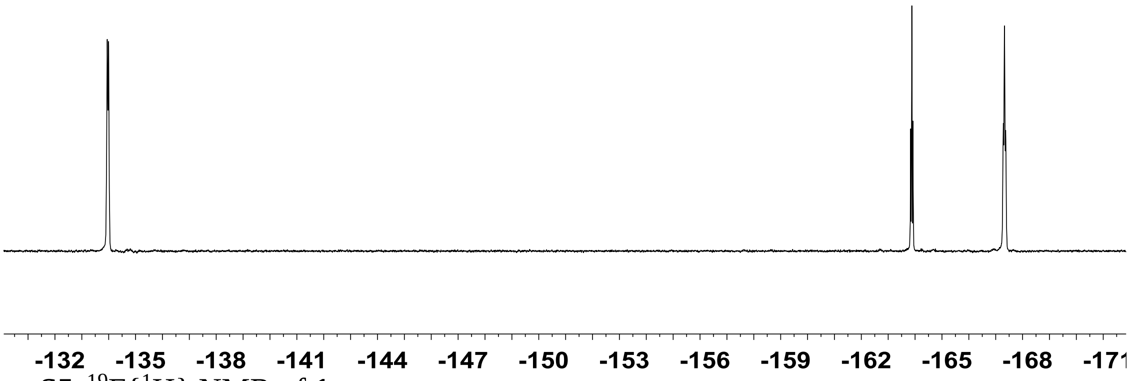

Figure S5. $^{19}F\{^1H\}$ NMR of **1**

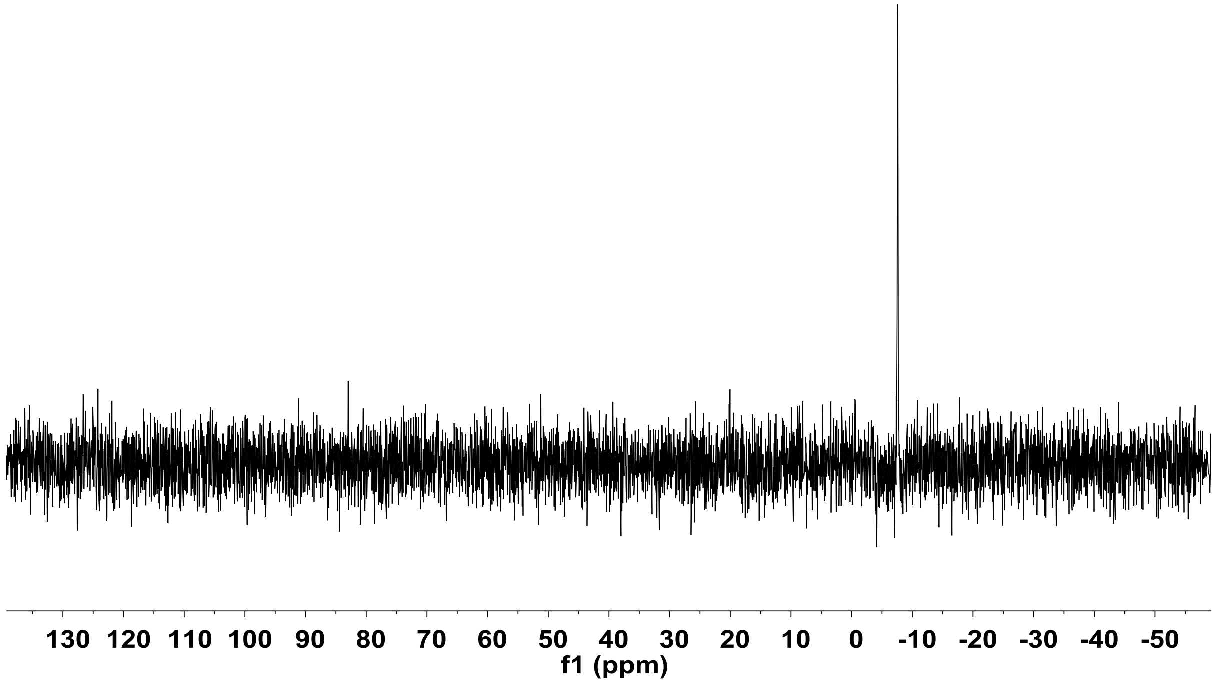

Figure S6. $^{11}B\{^1H\}$ NMR of **1**

For **2**:

^{1}**H NMR** (400MHz, C_6D_6, 298 K, pentane omitted): δ 2.43 (s, br, 2H, CH_2-B), 0.88 (s, 10H, -CH_3), 0.79 (s, 6H, -CH_3) 0.67 (s, 6H, -CH_3). ^{13}**C{^{1}H} NMR** (128 MHz, C_6D_6, 298 K): δ 93.1, 92.3, 91.9, 91.7, 7.1 (-*C*H$_3$), 6.8 (-*C*H$_3$), 6.7 (-*C*H$_3$), 6.6 (-*C*H$_3$). ^{19}**F{^{1}H} NMR** (C_6D_6, 377 MHz, 298 K): δ -130.2 (s, br, 6F, *o*–C_6F_5), -161.9 (t, 3F, *p*–C_6F_5, $^3J_{FF}$ = 26 Hz), -162.7 (s, br, 6F, *m*–C_6F_5). ^{11}**B{^{1}H} NMR** (C_6D_6, 128 MHz, 298 K): δ -13.0.

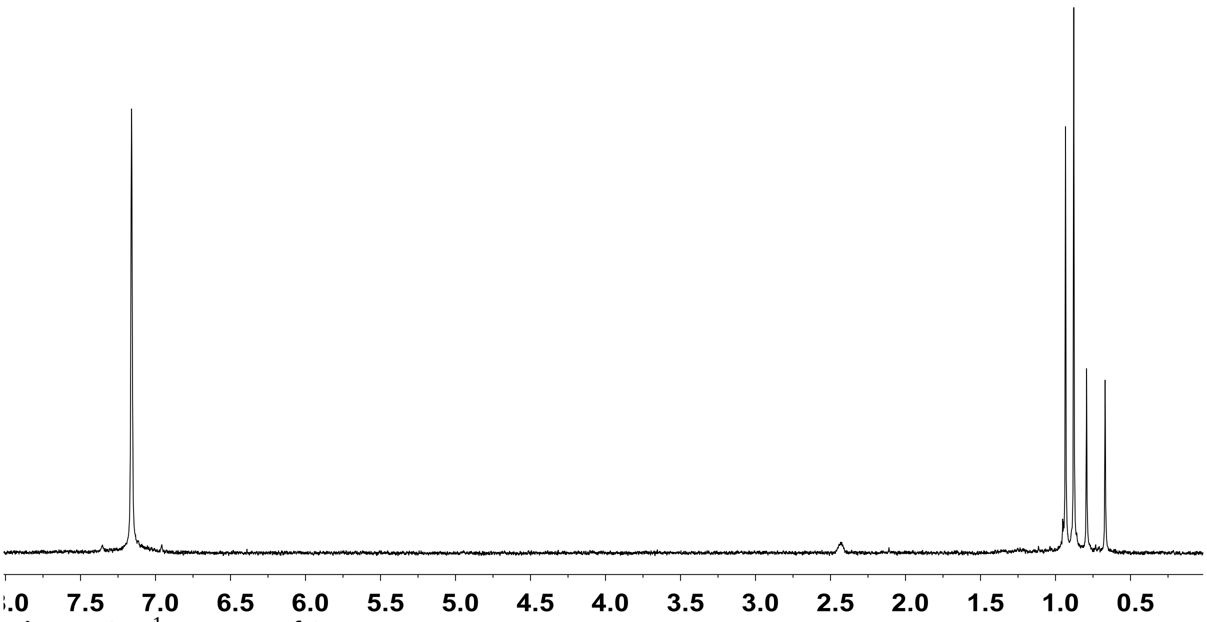

Figure S7. ^{1}H NMR of **2**

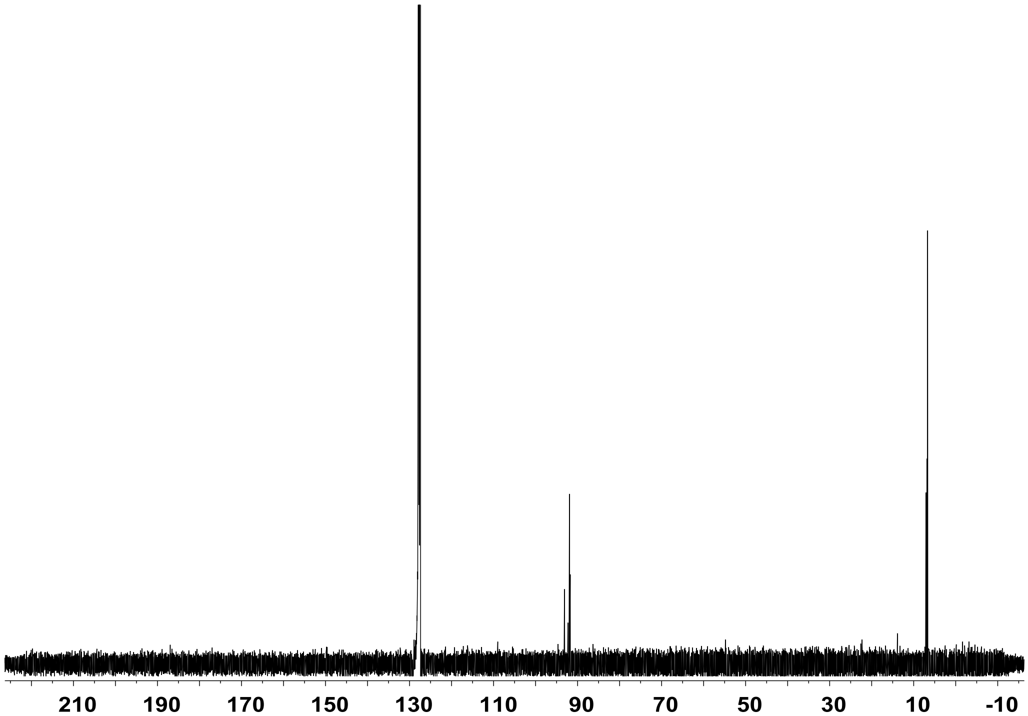

Figure S8. $^{13}C\{^{1}H\}$ NMR of **2**

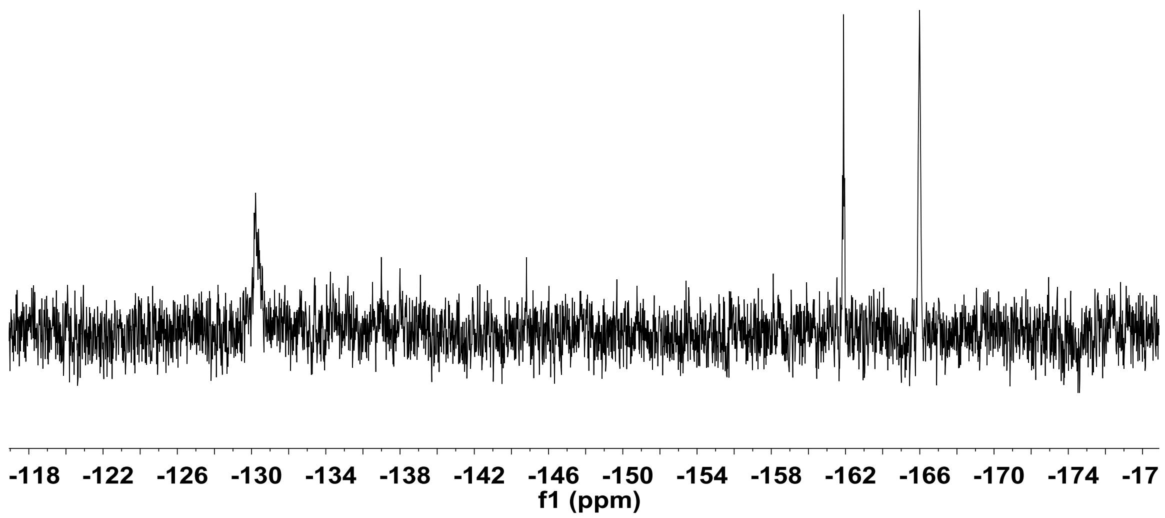

Figure S9. $^{19}F\{^1H\}$ NMR of **2**

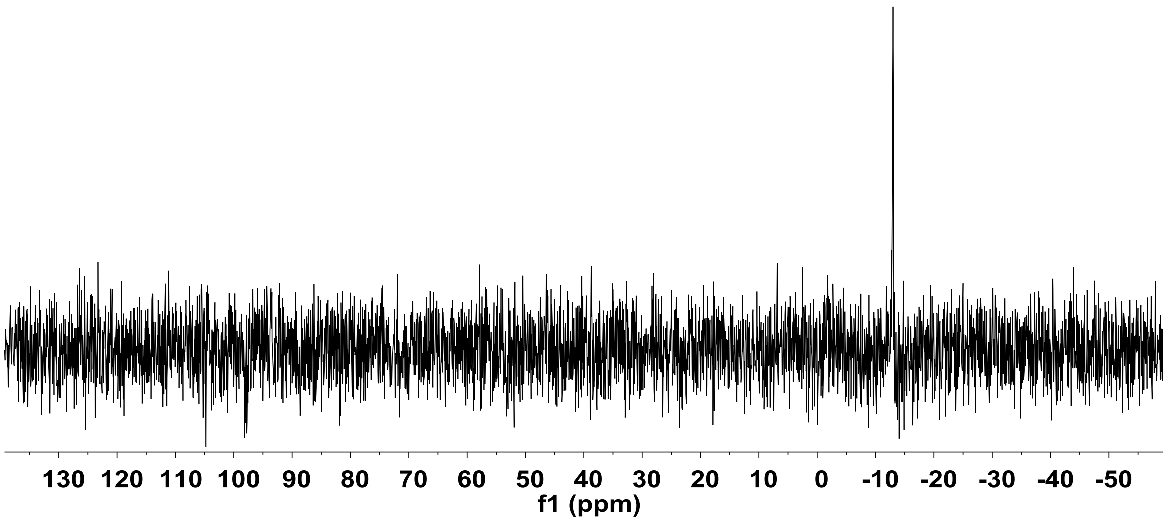

Figure S10. $^{11}B\{^1H\}$ NMR of **2**

Synthesis of [Cp*$_2$Cr][(PhC(C_6H_4)NNBPh$_3$] (3) and [Cp*$_2$Cr][Ph$_2$CNNHBPh$_3$] (4)

A solution of Ph_2CN_2 (55.4 mg, 0.285 mmol) in 2 mL of chlorbenzene was stirred in a vial at -35℃, while BPh_3 (69.1 mg, 0.285 mmol) and Cp*$_2$Cr (92.0 mg, 0.285 mmol) were added sequentially, resulting an orange solid started to crashed out from the solution. The reaction mixture was allowed to further stir for another 2 h at room temperature to ensure the reaction is fully completed. The solvent was removed under vacuum, and the resulting solid was washed with 6 mL of pentane, and extracted with 3 × 2 mL of benzene and dried in *vacuo*. The yield orange solid was further washed with 3 × 2 mL of pentane and dried in *vacuo* to give clean compound **3** (45.6 mg, Yield 42%). The resulting leftover solid after extraction of compound **3** was further washed with 2 mL of chlorobenzene and 3 × 2 mL of pentane, and dried in *vacuo* to give compound **4** (54.2 mg, Yield 50%)

Single crystals for X-ray studies were obtained from slow evaporation of a pentane into a saturated chlorobenzene solution for **3** and a saturated DFB solution of **4** at room temperature to give orange crystals.

For **3**:

^{1}**H NMR** (400MHz, C_6D_6, 298 K, pentane and toluene are omitted): δ 21.35 (s, br, 30H, -C*H*$_3$), 8.44 - 7.09(m, br, 24H), 5.88 (s, br, 2H), 5.07 (s, br, 2H). ^{13}**C{^{1}H} NMR** (128 MHz, C_6D_6, 298 K, partial due to the paramagnetic cation Cp*$_2$Cr, pentane ommitted): δ 143.1, 138.4, 117.6. ^{11}**B{^{1}H} NMR** (C_6D_5Br, 128 MHz, 298 K): δ 23.1.

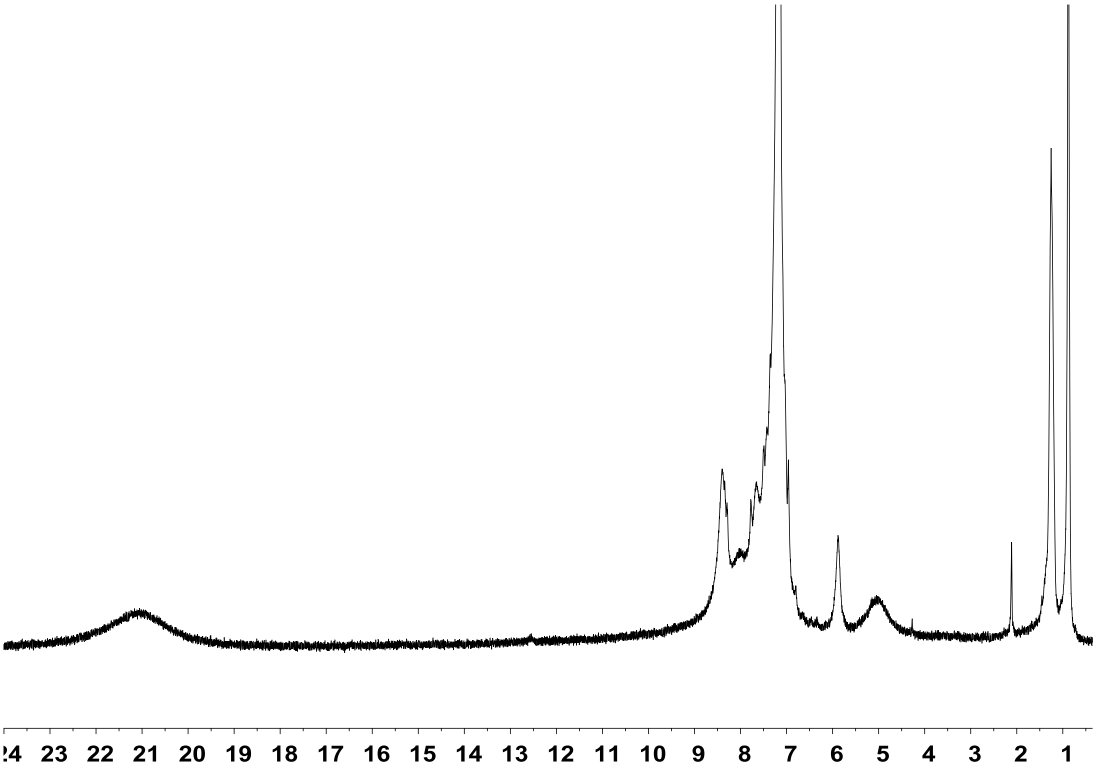

Figure S11. ^{1}H NMR of **3**

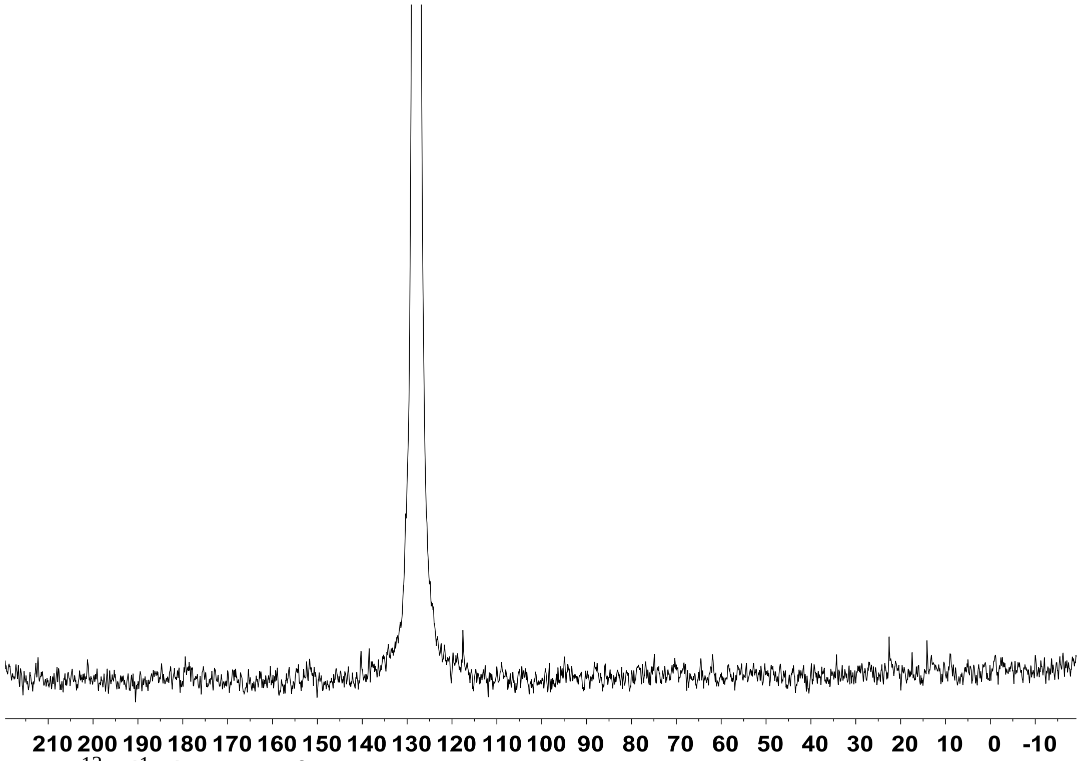

Figure S12. ^{13}C{^{1}H} NMR of **3**

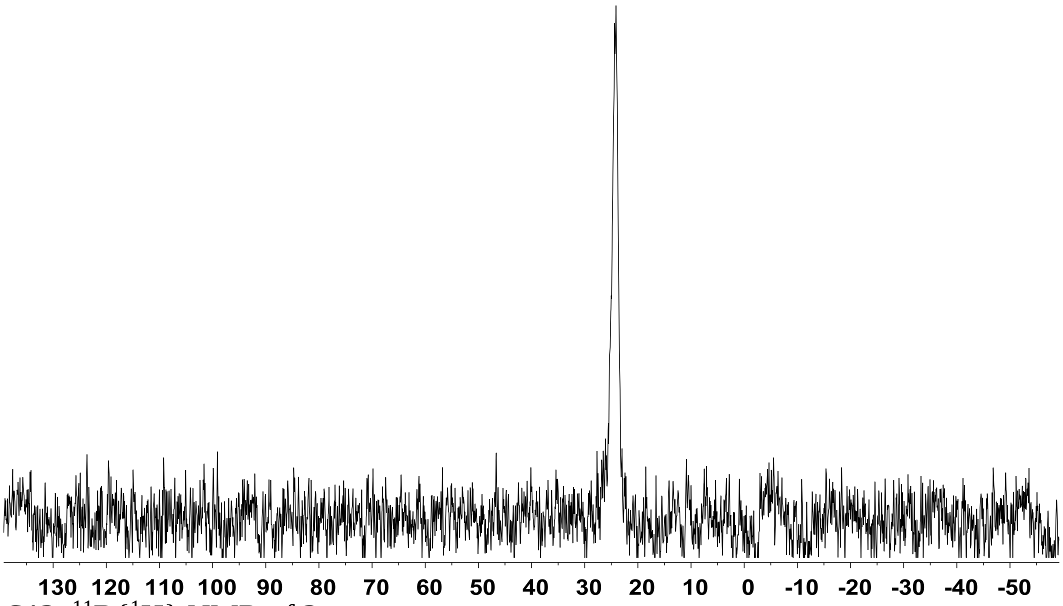

Figure S13. ^{11}B{^{1}H} NMR of **3**

For **4**:

^{1}H NMR (400MHz, $CDCl_3$, 298 K, pentane and toluene are omitted): δ 20.51 (s, br, 30H, -CH_3), 8.23 - 6.41(m, br, 25H), 3.42 (m, br, 1H, -N*H*-). **^{13}C{^{1}H} NMR** (128 MHz, $CDCl_3$, 298 K): δ 135.0, 134.6, 132.8, 132.4, 130.8, 129.2, 128.8, 128.5, 128.4, 126.8, 126.6, 126.0, 125.5, 125.2, 123.3, 121.8. **^{11}B{^{1}H} NMR** ($CDCl_3$, 128 MHz, 298 K): δ -3.7.

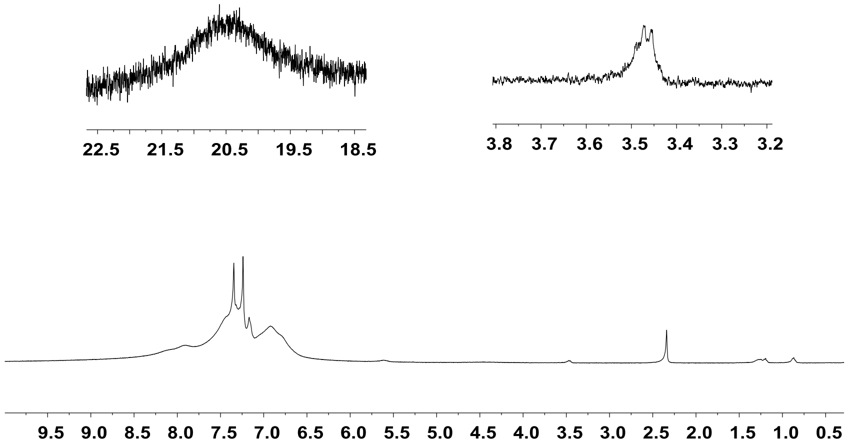

Figure S14. ^{1}H NMR of **4**

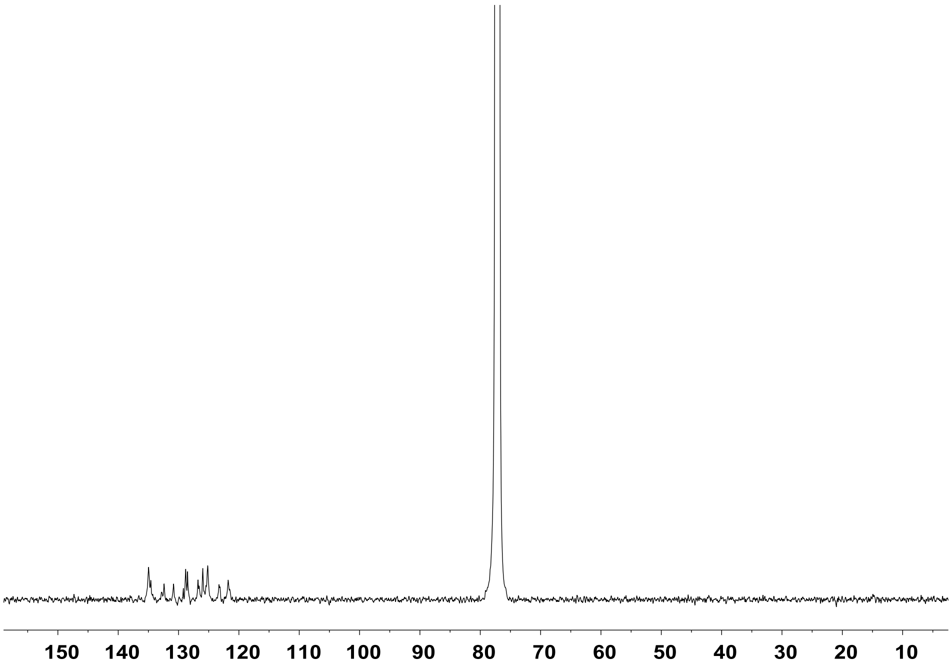

Figure S15. $^{13}C\{^1H\}$ NMR of **4**

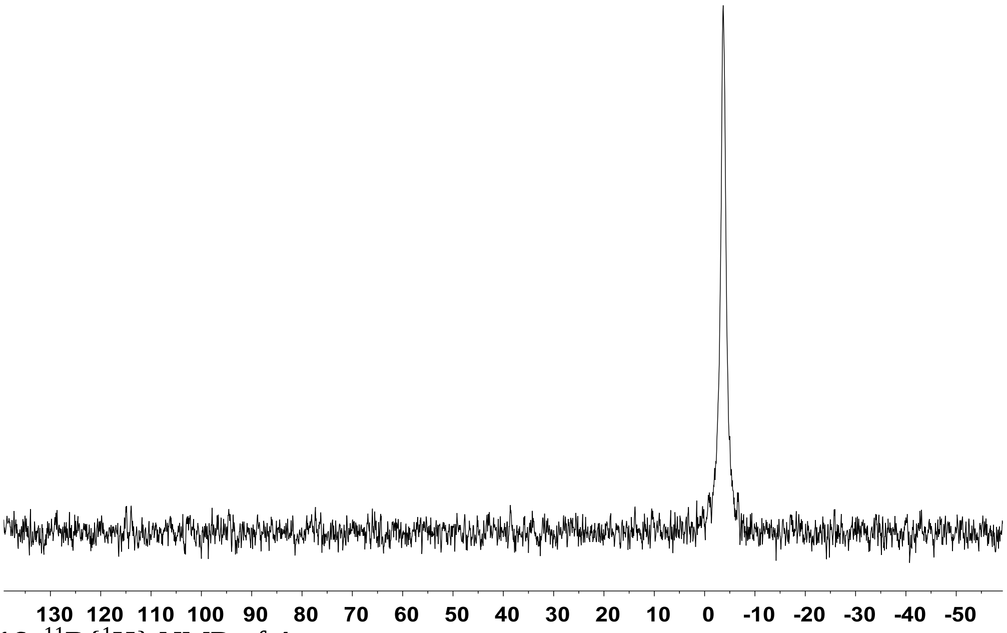

Figure S16. $^{11}B\{^1H\}$ NMR of **4**

Synthesis of $(C_{12}H_8)C(C_6F_5)(B(C_6F_5)_2)$

A solution of $B(C_6F_5)_3$ (94.3 mg, 0.184 mmol) in 2 mL of pentane was added slowly to a solution of $(C_{12}H_8)CN_2$ (35.4 mg, 0.184 mmol) in 2 mL of pentane. The solution was then stored in a -35 °C freezer for one days to give colorless crystals, which is suitable for diffraction study. The crystals was then washed with -35 °C pentane (3 × 2 mL) to give clean $(C_{12}H_8)C(C_6F_5)(B(C_6F_5)_2)$ (67.8 mg, 54 %).

^{1}H NMR (400MHz, C_6D_6, 298 K): δ 7.63 (d, $^3J_{HH}$ = 7.3 Hz, 2H), 7.32 (d, $^3J_{HH}$ = 7.1 Hz, 2H), 7.11 (pd, $^4J_{HH}$ = 1.5 Hz, $^3J_{HH}$ = 7.4 Hz, 4H). **$^{13}C\{^1H\}$ NMR** (128 MHz, C_6D_6, 298 K): δ 147.3 (d, $^1J_{CF}$ = 244 Hz, C_6F_5), 144,1 (s, $C_{12}H_8$), 142.9 (d, $^1J_{CF}$ = 254 Hz, C_6F_5), 142.5 (s, $C_{12}H_8$), 141.2 (s, $C_{12}H_8$), 139.6 (d, $^1J_{CF}$ = 244 Hz, C_6F_5), 137.8 (d, $^1J_{CF}$ = 240 Hz, C_6F_5), 137.0 (d, $^1J_{CF}$ = 254 Hz, C_6F_5), 129.6 (s, $C_{12}H_8$), 127.0 (s, $C_{12}H_8$), 124.0 (s, $C_{12}H_8$), 120.4 (s, $C_{12}H_8$), 57.8 (s, *C*-$(C_6F_5)(B(C_6F_5)_2)$. **^{19}F NMR** (377 MHz, C_6D_6, 298K): δ -125.6 (s, br, 6F), -137.2 (s, br, 1F), -146.6 (s, br, 2F), -155.2 (t, $^3J_{FF}$ = 22 Hz, 2F), -161.4 (s, 4F). **$^{11}B\{^1H\}$ NMR** (128 MHz, C_6D_6, 298 K): δ 61.6 (s, br).

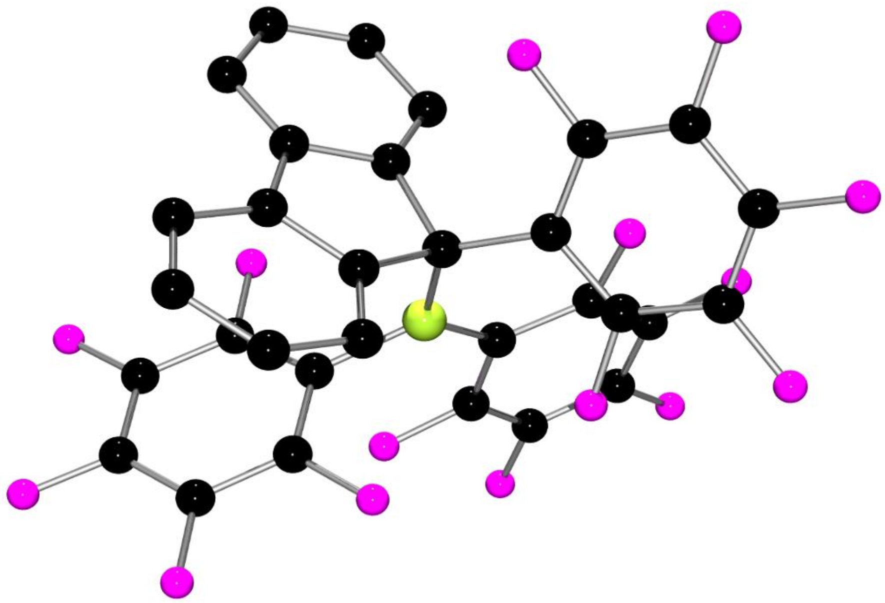

Figure S17. POV-Ray depiction of $(C_{12}H_8)C(C_6F_5)(B(C_6F_5)_2)$. The hydrogen atoms are omitted for clarity. C: black, F: pink B: yellow-green.

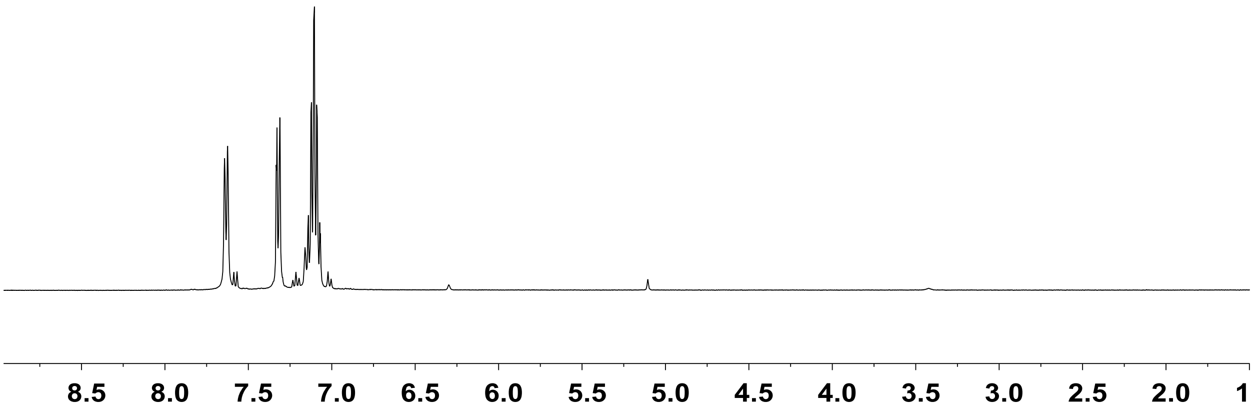

Figure S18. ^{1}H NMR of $(C_{12}H_8)C(C_6F_5)(B(C_6F_5)_2)$.

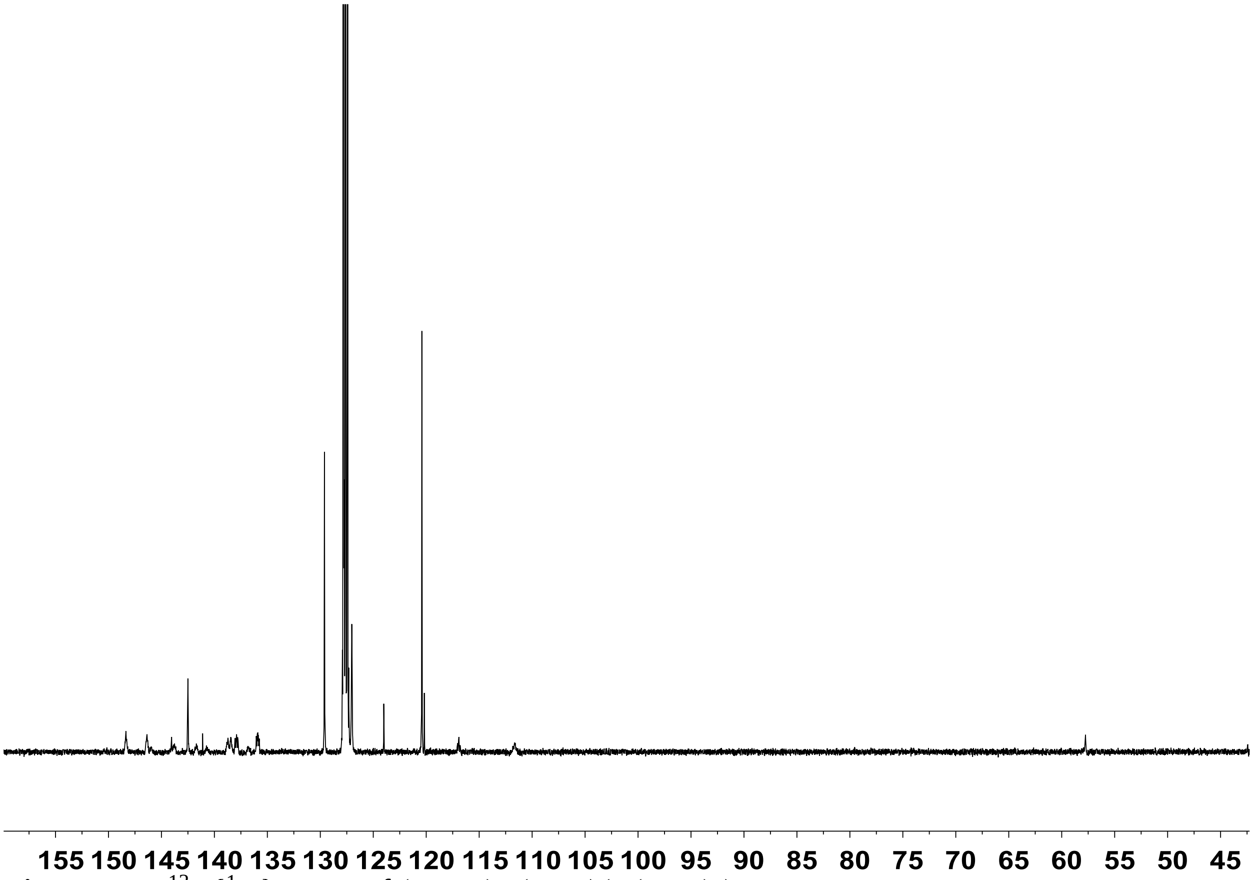

Figure S19. $^{13}C\{^{1}H\}$ NMR of $(C_{12}H_8)C(C_6F_5)(B(C_6F_5)_2)$.

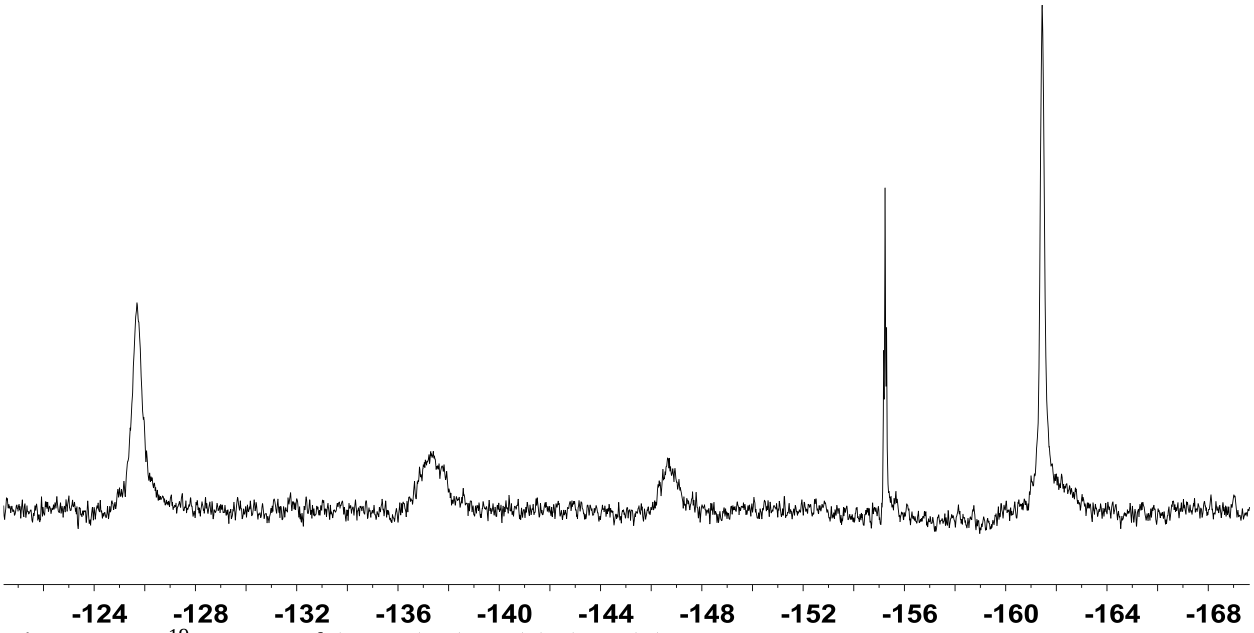

Figure S20. ^{19}F NMR of $(C_{12}H_8)C(C_6F_5)(B(C_6F_5)_2)$.

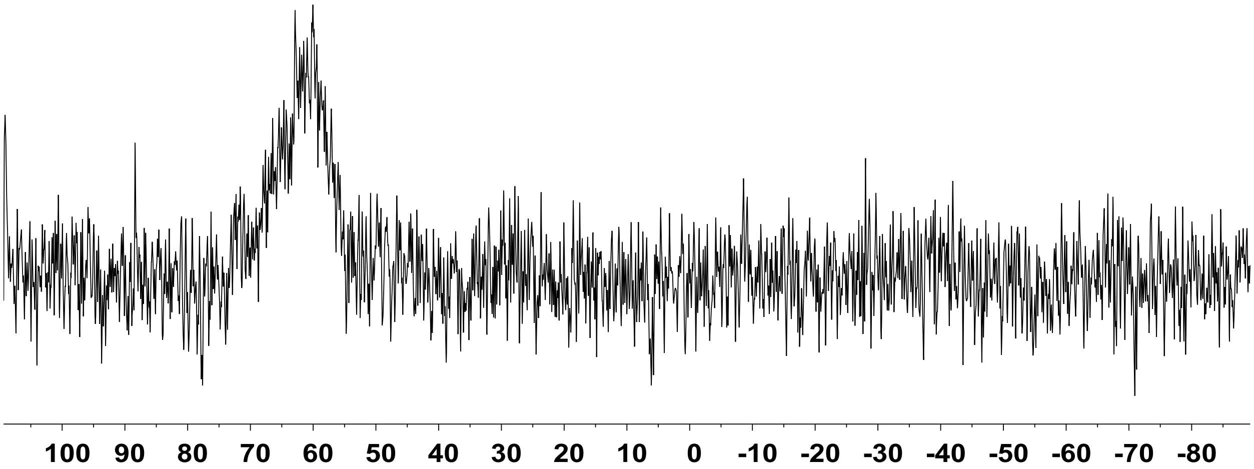

Figure S21. $^{11}B\{^1H\}$ NMR of $(C_{12}H_8)C(C_6F_5)(B(C_6F_5)_2)$.

Generation of $[Cp^*_2Cr][C_{12}H_8CNNHBPh_3]$ (5), $[Cp^*_2Cr][C_{13}H_7N_2(BPh_3)]$ (6) and $[Cp^*Cr(C_5Me_4CH_2BPh_3)]$ (7)

A solution of $(C_{12}H_8)CN_2$ (54.7 mg, 0.285 mmol) in 2 mL of chlorobenzene was stirred in a vial at -35 °C, while BPh_3 (69.1 mg, 0.285 mmol) and Cp^*_2Cr (92.0 mg, 0.285 mmol) were added sequentially, resulting in the precipitation of an orange solid. The reaction mixture was allowed to further stir for another 2 h at room temperature to ensure the reaction is fully completed. The solid was filtered and further washed with 3 × 2 mL of C_6H_5Cl and 3 × 2 mL of pentane and dried under vacuum to give crude compound of **5**. The solvent used to wash **5** were collected and removed under vacuum. The resulting solid was dissolved in C_6H_5Cl and layered with pentane at room temperature to give a mixture of crystals for **5** and **7** suitable for diffraction study. Single crystals suitable for X-ray studies for **5** were obtained from a saturated DFB solution layered with pentane at room temperature (56 mg 52 %), while clean **6** and **7**, were not isolated in pure form due to their similar solubilities.

For **5**:
^{1}H NMR (400MHz, $CDCl_3$, 298 K,): signals were broad due to the paramagnetic $[Cp^*_2Cr]^+$ cation, and low solubility of **5**. **$^{13}C\{^1H\}$ NMR** (128 MHz, $CDCl_3$, 298 K, partial data due to

solubility of **5**, and the paramagnetic [Cp*$_2$Cr]$^+$ cation): δ 160.3, 135.9, 132.2, 131.1, 129.7, 128.9. **^{11}B{^{1}H} NMR** (CDCl$_3$, 128 MHz, 298 K): δ -1.7.

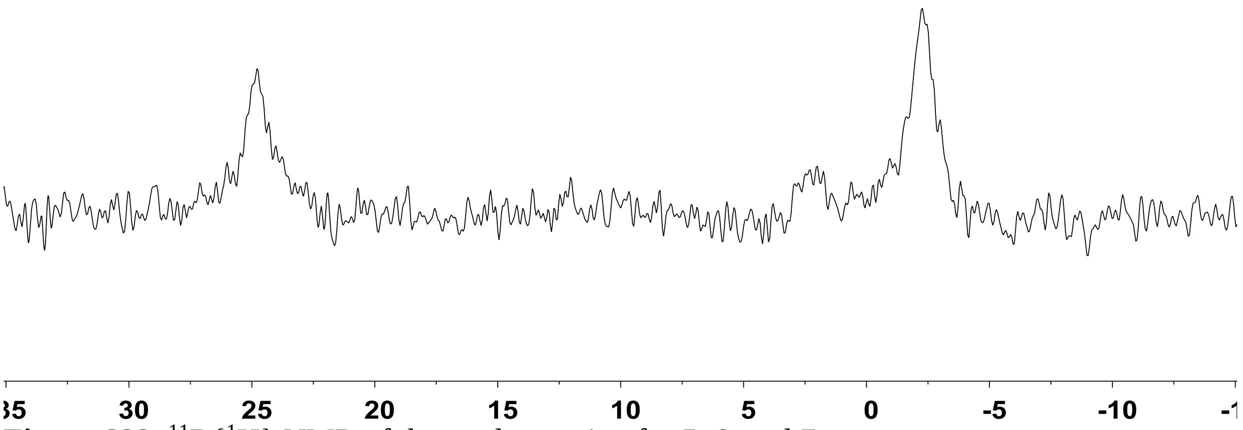

Figure S22. ^{11}B{^{1}H} NMR of the crude reaction for **5**, **6**, and **7**.

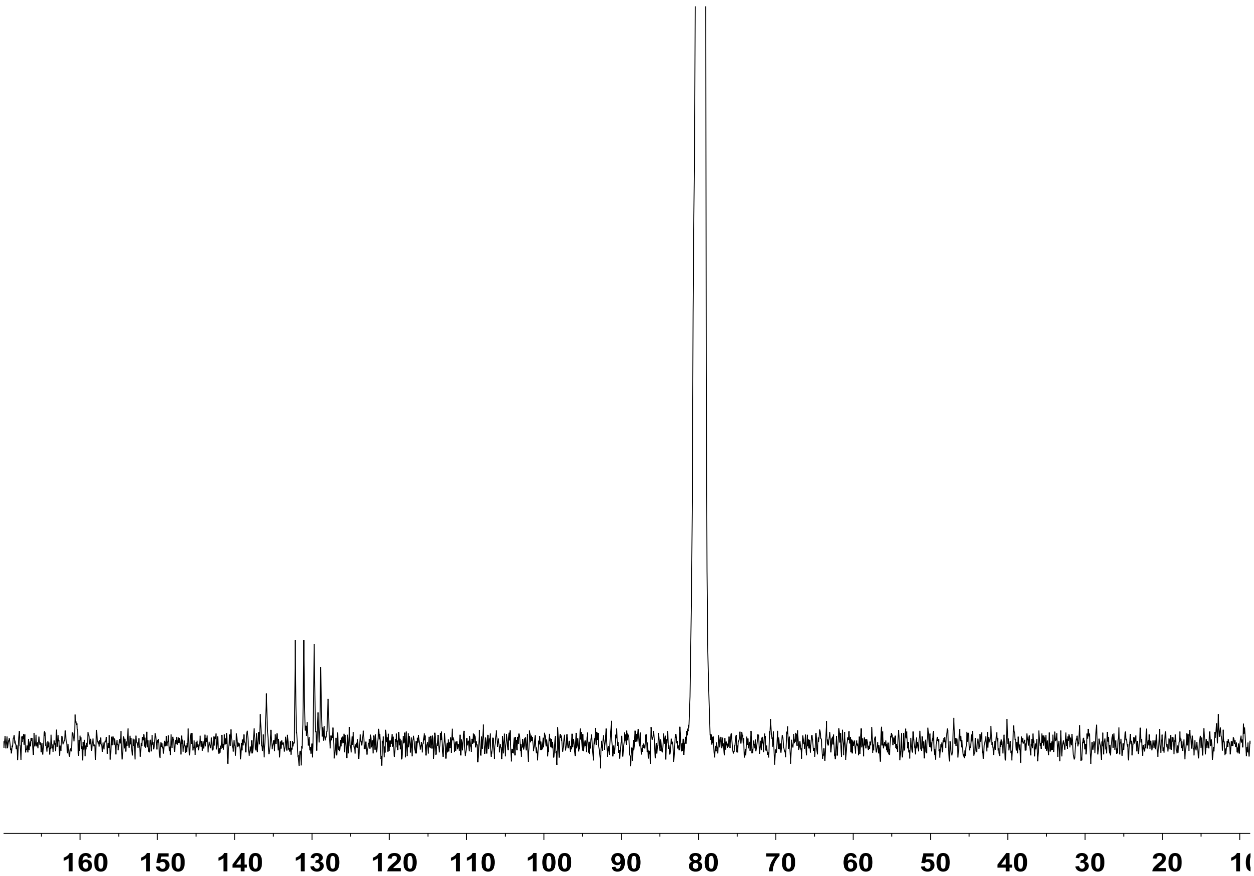

Figure S23. ^{13}C{^{1}H} NMR of **5**.

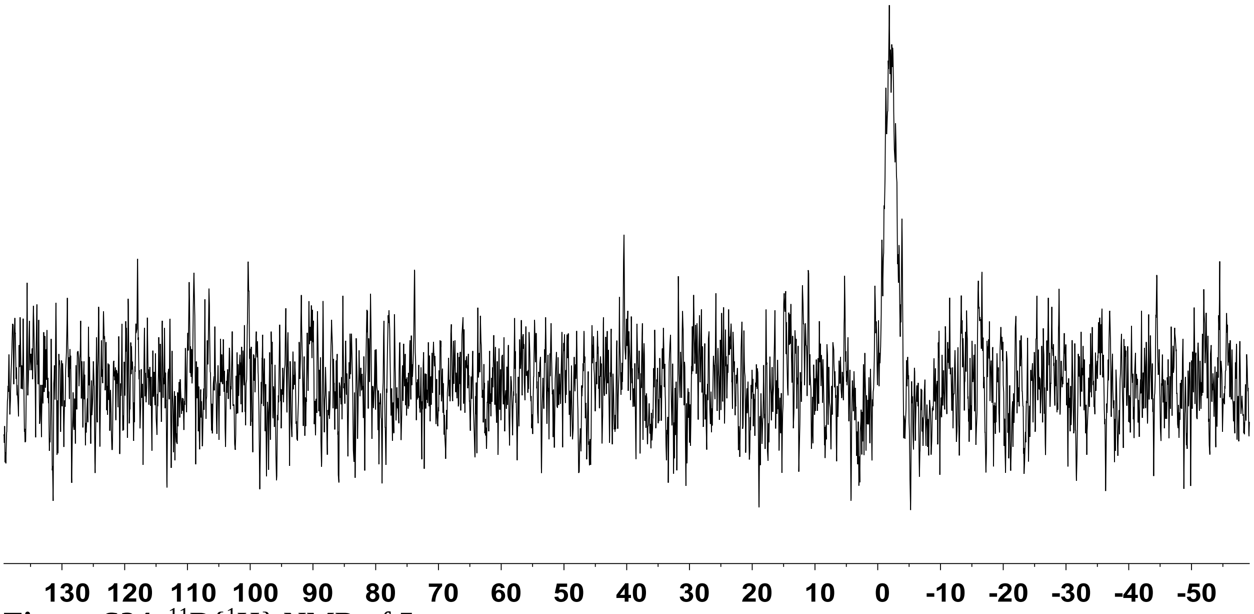

Figure S24. $^{11}B\{^{1}H\}$ NMR of **5**.

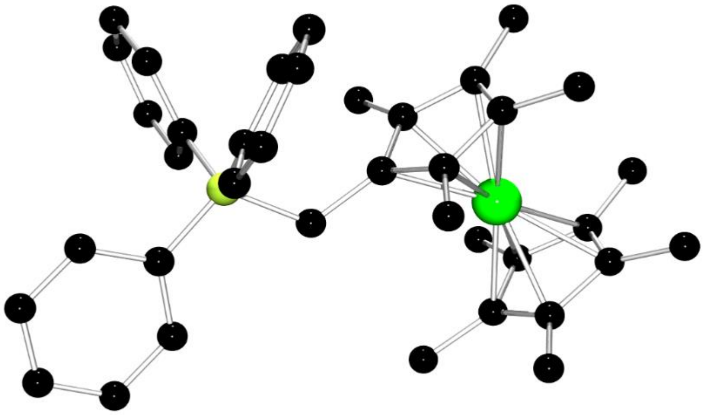

Figure S25. POV-Ray depiction of **6**. The hydrogen atoms are omitted for clarity. C: black, Cr: green B: yellow-green.

Synthesis of [Cp*$_2$Fe][$C_{12}H_8$CNNH(Al(C_6F_5)$_3$)] (8)

A solution of Al(C_6F_5)$_3$ (27.8 mg, 0.0927 mmol) and Cp*$_2$Fe (30.3 mg, 0.0927 mmol) in 1 mL of C_6H_5Cl was added slowly to a solution of ($C_{12}H_8$)CNNH$_2$ (18.0 mg, 0.0927 mmol) in 1 mL of C_6H_5Cl. The solution immediately turned from light yellow to deep green. After stirring at room temperature for 30 min, 6 mL of pentane was added to give green participated. The solvent was then carefully decanted with a pipette, and the solid was further washed with 3 × 2 mL of pentane and dried *in vacuo*. Crystals suitable for X-ray studies was obtained from slow diffusion of pentane into a saturated DFB solution of **8** (42 mg, 43 %).

^{1}H NMR (400MHz, C_6D_5Br, 298 K, signals were broad due to the paramagnetic $[Cp*_2Fe]^+$ cation, and low solubility of **8**): δ 29.5 (s, br, 30H, C*H*$_3$), 11.98 – 6.01 (m, br, 9H). **^{13}C{^{1}H} NMR** (128 MHz, C_6D_6, 298 K, partial data due to solubility of **8**, and the paramagnetic $[Cp*_2Fe]^+$ cation): δ 154.6 (d, $^1J_{CF}$ = 235 Hz, *C*$_6$F$_5$), 150.7 (d, $^1J_{CF}$ = 241 Hz, *C*$_6$F$_5$), 136.2 (d, $^1J_{CF}$ = 251 Hz, *C*$_6$F$_5$). **^{19}F NMR** (377 MHz, C_6D_6, 298K): δ -122.2 (s, br, 6F), -154.3 (s, br, 3F), -162.0 (s, br, 6F). **^{27}Al{^{1}H} NMR** (104 MHz, C_6D_5Br, 298 K): no signal detected.

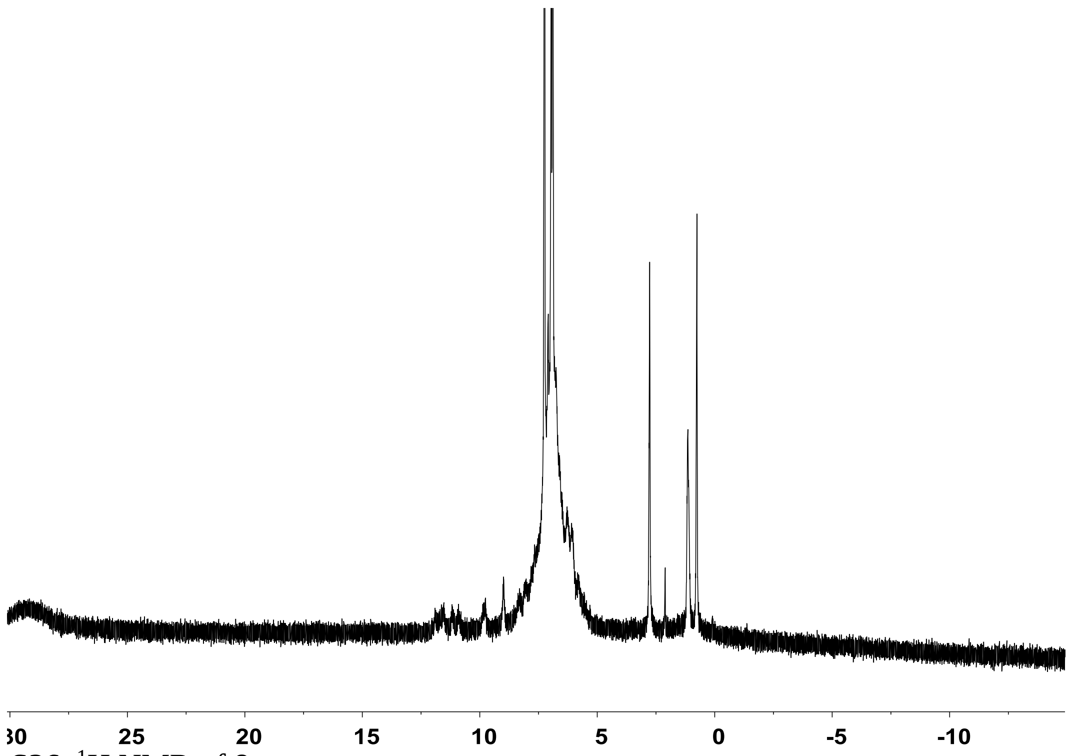

Figure S26. ^{1}H NMR of **8**.

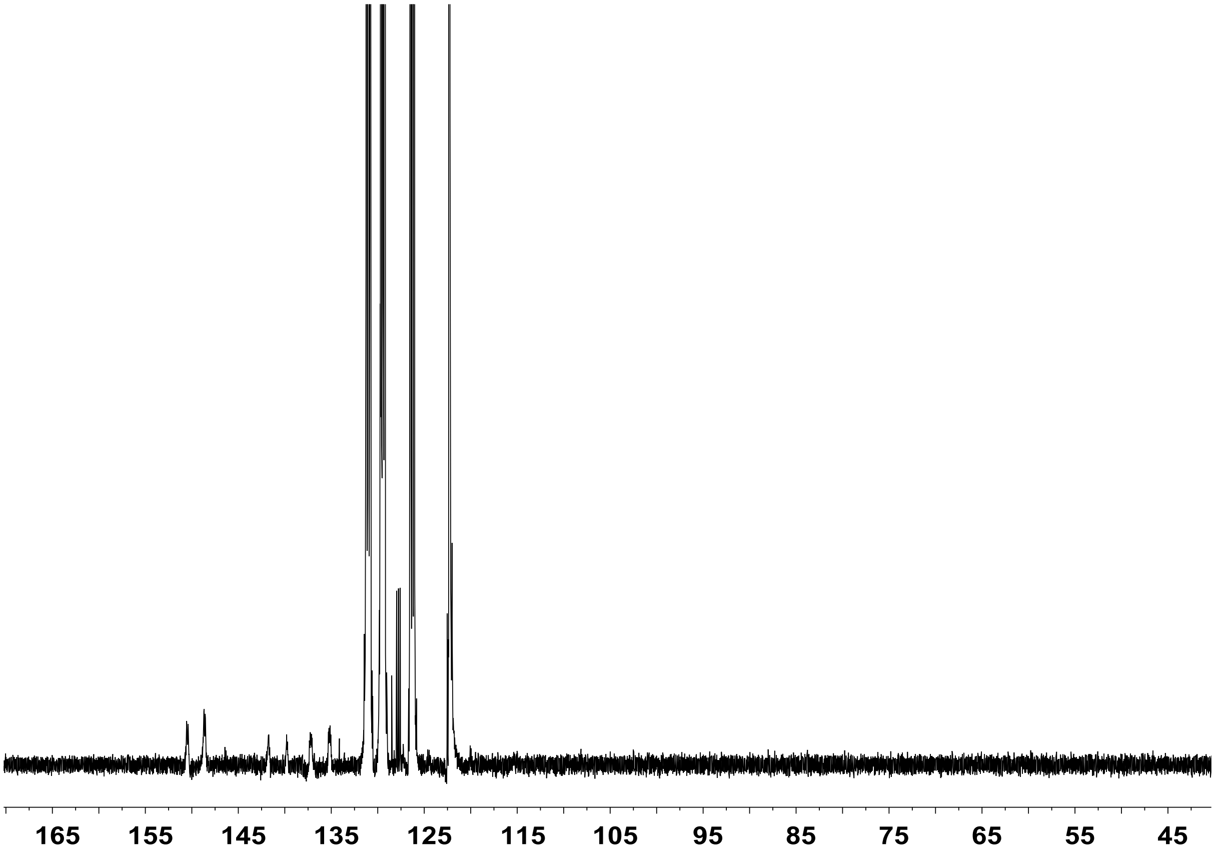

Figure S27. $^{13}C\{^1H\}$ NMR of **8**.

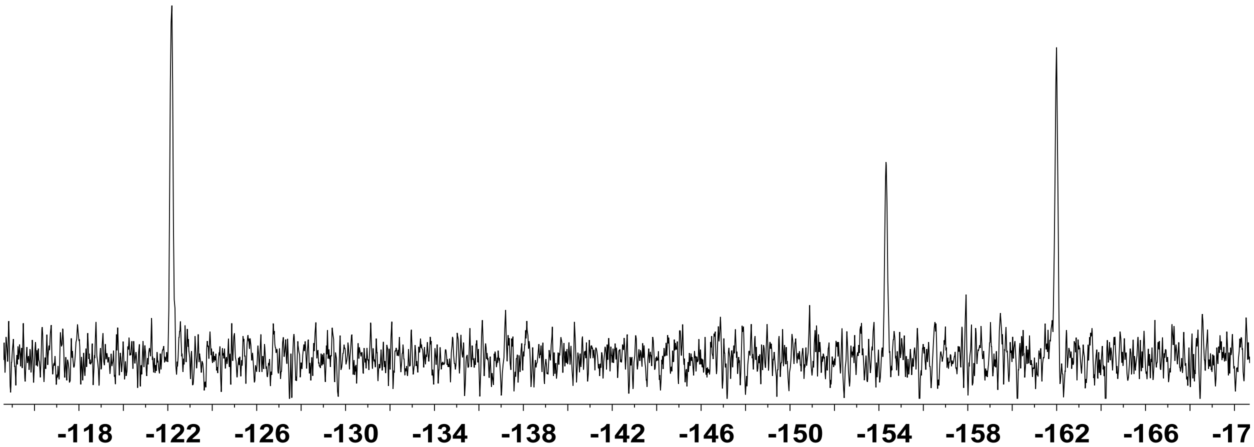

Figure S28. ^{19}F NMR of **8**.

Generation of 8 and [Cp*$_2$Fe][($C_{12}H_8$)CH(Al(C_6F_5)$_3$)] (9)

A solution of Al(C_6F_5)$_3$ (31.4 mg, 0.0546 mmol) and Cp*$_2$Fe (17.8 mg, 0.0546 mmol) in 1 mL of C_6H_5Cl was added slowly to a solution of ($C_{12}H_8$)CN_2 (10.5 mg, 0.0546 mmol) in 1 mL of C_6H_5Cl. The solution immediately turned from light yellow to deep green, the EPR spectra for the reaction was quickly obtained (For 5 min and 5 h at room temperature as shown in Figure S29). Addition of a solution of Ph_3SnH (19.2 mg, 0.0546 mmol) in 1 mL of C_6H_5Cl. The reaction mixture was allowed to stir at room temperature for another hour. The volatiles were then removed under vacuum. While many products were generated in the reaction as evidenced by ^{19}F NMR since the mixture of Cp*$_2$Fe and Al(C_6F_5)$_3$ could also react with Ph_3SnH, the generation of **8** was identified by ^{19}F NMR, while the crystals of **9** that is suitable for X-ray studies were grow from layer a solution of the reaction mixture in C_6H_5Cl with pentane at -35 °C temperature.

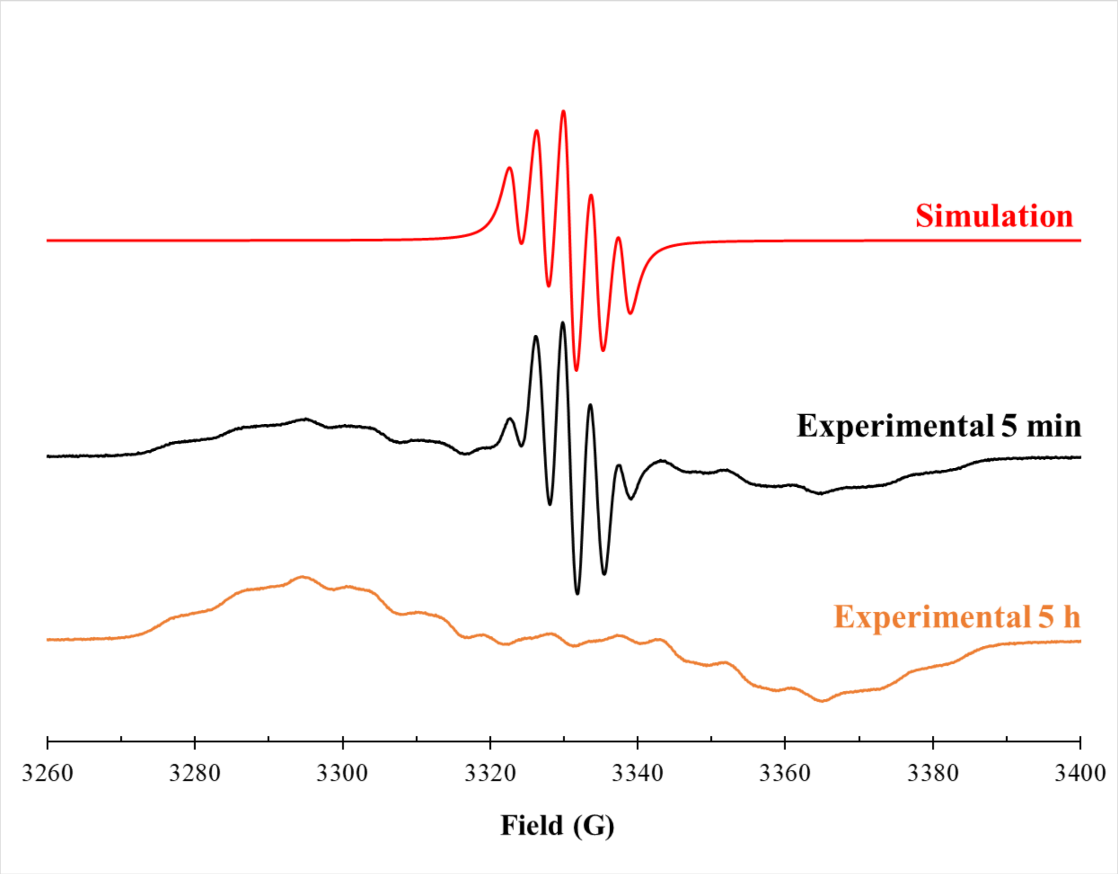

Figure S29. Experimental and simulation EPR spectra of the reaction crude for generation of **8** and **9**.

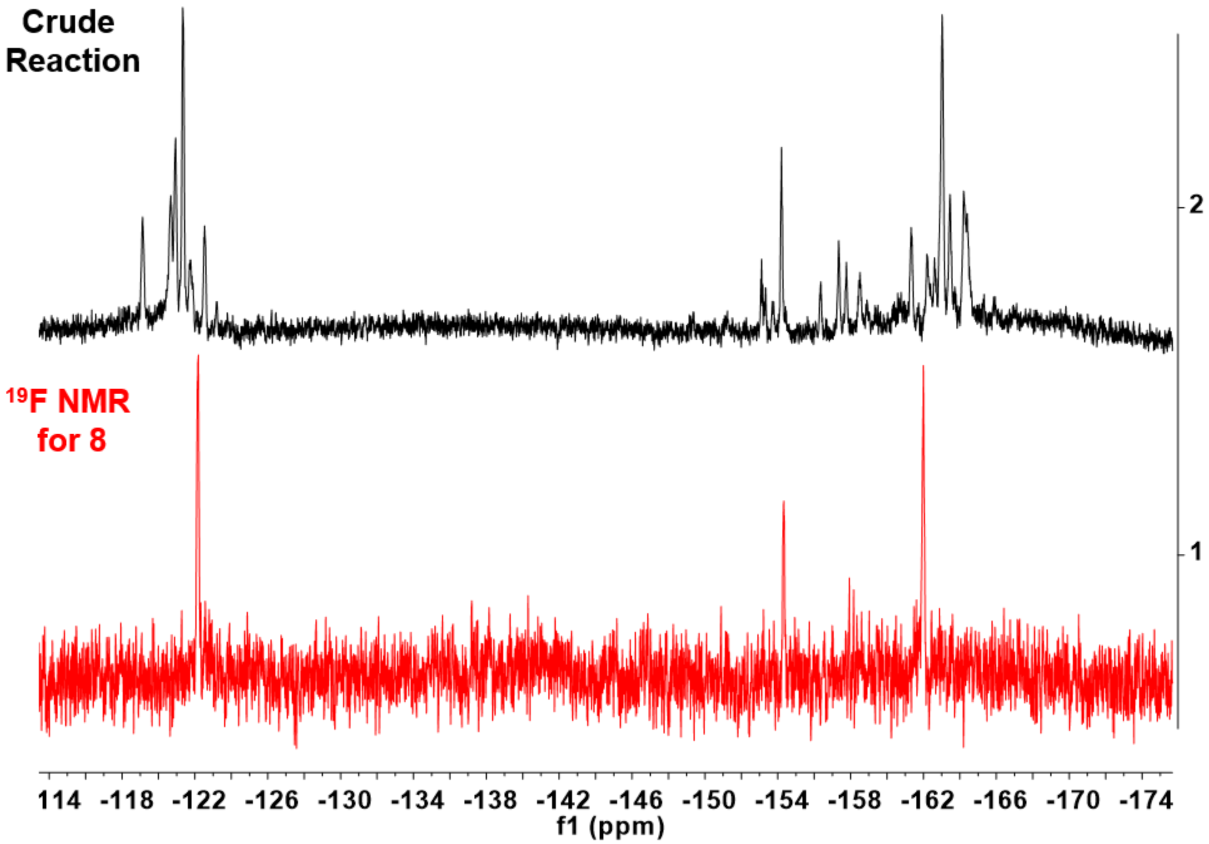

Figure S30. Stacked ^{19}F NMR spectra for the crude reaction comparing with **8**.

2. X-ray Crystallography

X-ray Data Collection and Reduction. Crystals were coated in Paratone-N oil in an N_2 filled glovebox, mounted on a MiTegen Micromount, and placed under a N_2 stream, thus maintaining a dry, O_2-free environment for each crystal. The data were collected on a Bruker Apex II diffractometer using a graphite monochromator with Mo Kα radiation (λ = 0.71073 Å). The data were collected at 150(2) K for all crystals. The frames were integrated with the Bruker SAINT software package using a narrow-frame algorithm. Data were corrected for absorption effects using the empirical multiscan method (SADABS).

Structure Solution and Refinement. The structures were solved by intrinsic phasing using XS. All the structures were subjected to full-matrix least-squares refinement on F^2 using XL as implemented in the SHELXTL suite of programs. All non-hydrogen atoms were refined with anisotropically thermal parameters. Carbon bound hydrogen atoms were placed in geometrically calculated positions and refined using an appropriate riding model and coupled isotropic thermal parameters. For compound **1, 4, 5**, and **8**, the N bound hydrogen atoms and H bound to the C center that is connected with Al of **9** were located through electron density difference map and modeled with appropriate riding model and coupled isotropic thermal parameters.

For compound **1**, the chlorobenzene solvent molecule was modeled as disordered over an inversion center, while for compound **4**, one of the Cp* ring was modeled as position disorder with a ratio of 42:58.

For compound **7**, there are two disordered chlorobenzene solvent molecules modeled in the asymmetric unit. While one of the chlorobenzene molecules was modeled as a positional disorder over an inversion center with Part -1, the other chlorobenzene molecule was treated as simple positional disorder with free variable method resulting a ratio of 43:57.

For compound **8**, one of the Cp* anion on the $[Cp^*_2Fe]^+$ cation was modeled as a positional disorder with a ratio of 43:57.

Table S1. Summary of crystallographic data for compounds **1 - 3.**

	**1**	**2**	**3**
empirical formula	C54 H43.50 B Cl 0.50 Co F15 N2	C38 H29 B Co F15	C51 H54 B Cr N2
formula weight	1092.87	840.35	757.77
crystal system	Triclinic	Monoclinic	Triclinic
space group	$P\bar{1}$	$P2_1/n$	$P\bar{1}$
a (Å)	11.9356(9)	11.506(2)	11.353(2)
b (Å)	11.961(2)	17.317(2)	11.402(2)
c (Å)	18.574(2)	17.589(2)	18.323(2)
α (deg.)	77.087(2)		99.080(4)
β (deg.)	84.528(2)	96.728(4)	103.704(4)
γ (deg.)	72.178(2)		111.170(4)
vol (Å^3)	2459.5(4)	3480.3(8)	2069.8(4)
Z	2	4	2
ρ (calcd) (Mg·cm^3)	1.476	1.604	1.216
μ (mm^{-1})	0.473	0.603	0.314
F(000)	1114	1696	806
Theta range (°)	2.789 to 26.372	2.241 to 25.682	2.576 to 28.283
T(K)	150(2)	150(2)	150(2)
reflections collected	50483	48902	39398
unique reflections	10022	6596	10267
R_{int}	0.1065	0.1438	0.0679
GOF (F^2)	1.058	0.940	1.050
R1 indices [I>2σ(I)]	0.0658	0.0705	0.0665
wR2 indices (all data)	0.1785	0.2237	0.2047
Largest diff. peak and hole (e. Å^{-3})	0.975 & -0.633	0.487 & -0.925	0.898 & -0.459
CCDC No.	1917670	1917669	1917668

Table S2. Summary of crystallographic data for compounds **4**, **5**, and **7**.

	**4**	**5**	**7**
empirical formula	C54 H58 B Cr F N2	C57 H59 B Cl Cr N2	C44 H49 B Cl Cr
formula weight	816.83	870.32	676.09
crystal system	Triclinic	Monoclinic	Triclinic
space group	$P\bar{1}$	$P2_1/n$	$P\bar{1}$
a (Å)	10.814(2)	10.9237(4)	9.8346(9)
b (Å)	11.942(3)	17.3262(7)	10.910(2)
c (Å)	19.224(4)	25.4816(8)	18.520(2)
α (deg.)	85.793(7)		94.323(3)
β (deg.)	84.136(7)	99.658(2)	94.455(3)
γ (deg.)	67.003(6)		108.612(3)
vol (Å^3)	2271.7(8)	4754.5(3)	1867.0(4)
Z	2	2	2
ρ (calcd) (Mg·cm^3)	1.194	1.216	1.203
μ (mm^{-1})	0.294	0.336	0.407
F(000)	868	1844	718
Theta range (°)	2.104 to 25.682	3.451 to 30.508	2.951 to 26.730
T(K)	150(2)	150(2)	150(2)
reflections collected	21869	42470	38536
unique reflections	8517	14470	7930
R_{int}	0.0447	0.1042	0.0707
GOF (F^2)	1.051	1.007	1.038
R1 indices [I>2σ(I)]	0.0600	0.0918	0.0732
wR2 indices (all data)	0.1788	0.2457	0.2150
Largest diff. peak and hole (e. Å^{-3})	0.379 & -0.518	1.261 & -0.804	0.921 & -0.889
CCDC No.	1917667	1955444	1955447

Table S3. Summary of crystallographic data for compounds **8**, **9**, and **$(C_{12}H_8)C(C_6F_5)(B(C_6F_5)_2)$.**

	**8**	**9**	**$(C_{12}H_8)C(C_6F_5)(B(C_6F_5)_2)$**
empirical formula	C51 H39 Al F15 Fe N2	C57 H44 Al Cl F15 Fe	C31 H8 B F15
formula weight	1047.67	1132.20	676.18
crystal system	Monoclinic	Monoclinic	Triclinic
space group	$P2_1/c$	$P2_1/c$	$P\bar{1}$
a (Å)	9.9270(8)	14.2675(9)	9.314(2)
b (Å)	22.685(2)	14.820(2)	10.638(2)
c (Å)	21.241(2)	23.893(2)	14.314(2)
α (deg.)			92.390(4)
β (deg.)	96.544(2)	93.259(4)	102.317(4)
γ (deg.)			110.658(4)
vol (Å^3)	4752.2(6)	5043.6(6)	1285.8(3)
Z	4	4	2
ρ (calcd) (Mg·cm^3)	1.464	1.491	1.746
μ (mm^{-1})	0.433	0.464	0.177
F(000)	2132	2308	668
Theta range (°)	2.038 to 26.729	2.879 to 26.022	2.410 to 26.019
T(K)	150(2)	150(2)	150(2)
reflections collected	72023	39347	22517
unique reflections	10073	9937	5025
R_{int}	0.0605	0.1188	0.0849
GOF (F^2)	1.024	1.026	1.017
R1 indices [I>2σ(I)]	0.0464	0.0679	0.0546
wR2 indices (all data)	0.1339	0.2008	0.1317
Largest diff. peak and hole (e. Å^{-3})	0.407 & -0.455	0.553 & -0.450	0.222 & -0.318
CCDC No.	1955445	1955448	195446

3. Computational Chemistry

Computational Details: The quantum chemical DFT calculations have been performed with the TURBOMOLE 7.3 suite of programs[2] The structures are fully optimized at the TPSS-D3/def2-TZVP + COSMO(PhCl) level of theory, which combines the TPSS meta-GGA density functional[3] with the BJ-damped DFT-D3 dispersion correction[4] and the def2-TZVP basis set,[5] using the Conductor-like Screening Model (COSMO) continuum solvation model[6] for PhCl solvent (dielectric constant ε = 5.7 and solvent diameter R_{solv} = 3.50 Å). The density-fitting RI-J approach[5a, 7] is used to accelerate the geometry optimization and numerical harmonic frequency calculations[8] in solution. The optimized structures are characterized by frequency analysis to identify the nature of located stationary points (no imaginary frequency for true minima and only one imaginary frequency for transition state) and to provide thermal corrections (at 298.15 K and 1 atm) according to the modified ideal gas–rigid rotor–harmonic oscillator model.[9] This choice of dispersion-corrected meta-GGA functional makes the efficient exploration of all potential reaction paths possible.

The final solvation free energies in PhCl are computed with the COSMO-RS solvation model[10] (parameter file: BP_TZVP_C30_1601.ctd) using the COSMOtherm program package[[11] on the above TPSS-D3 optimized structures, and corrected by +1.89 kcal·mol^{-1} to account for higher reference solute concentration of 1 mol·L^{-1} usually used in solution. To check the effects of the chosen DFT functional on the reaction energies and barriers, single-point calculations at the meta-GGA TPSS-D3[3] and hybrid-meta-GGA PW6B95-D3[12] levels are performed using a larger def2-QZVP basis set.[5b, 13] The final reaction Gibbs free energies (ΔG) are determined from the electronic single-point energies plus TPSS-D3 thermal corrections and COSMO-RS solvation free energies.

Since DFT calculations for redox potentials of transition metal complexes like $Cp*_2Co$, $Cp*_2Cr$ and Cp_2Fe could potentially suffer from their multi-reference nature of electronic structure, the experimental values of -1.88 and -1.45 and -0.53 V (-43.4, -33.4, -12.2 kcal/mol)[14] of the $Cp*_2Co/Cp*_2Co^+$, $Cp*_2Cr/Cp*_2Cr^+$ and $Cp*_2Fe/Cp*_2Fe^+$ redox pairs relative to Cp_2Fe/Cp_2Fe^+ in CH_3CN solution are used as benchmark. The DFT-computed values in CH_3CN are (-32.9, -31.6 and -10.6 kcal/mol) and (-47.8, -30.9 and -10.8 kcal/mol) at the PW6B95-D3 and TPSS-D3 levels (see Tables S1). Despite good agreements are observed for the $Cp*_2Cr/Cp*_2Cr^+$ and $Cp*_2Fe/Cp*_2Fe^+$ pairs, a +10.5 kcal/mol too positive value is found for the $Cp*_2Co/Cp*_2Co^+$ pair (poor description for the 19-valecene-electron $Cp*_2Co$) at the PW6B95-D3 level thus it should be used with cation. In PhCl solution, COSMO-RS calculations show that these computed values should be corrected by -2.9, -3.1 and 2.9 kcal/mol, respectively.

The final free energies from both DFT functionals are in good mutual agreement of −0.7 ± 3.3 kcal/mol (mean deviation ± standard deviation, excluding the $Cp*_2Co$ redox potential) but the reaction barriers at PW6B95-D3 level are usually 3.7 ± 1.9 kcal/mol higher (see Table S1 below). In open-shell cases including transition metal complexes, the final results are somewhat sensitive to the choice of DFT functional. In our discussion, the higher-level PW6B95-D3 Gibbs free energies (in kcal/mol, at 298.15 K and 1 mol/L standard state concentration) will be used in our discussion unless specified otherwise, since meta-GGA functionals usually underestimate reaction barriers that could be improved by using hybrid meta-GGA functionals.

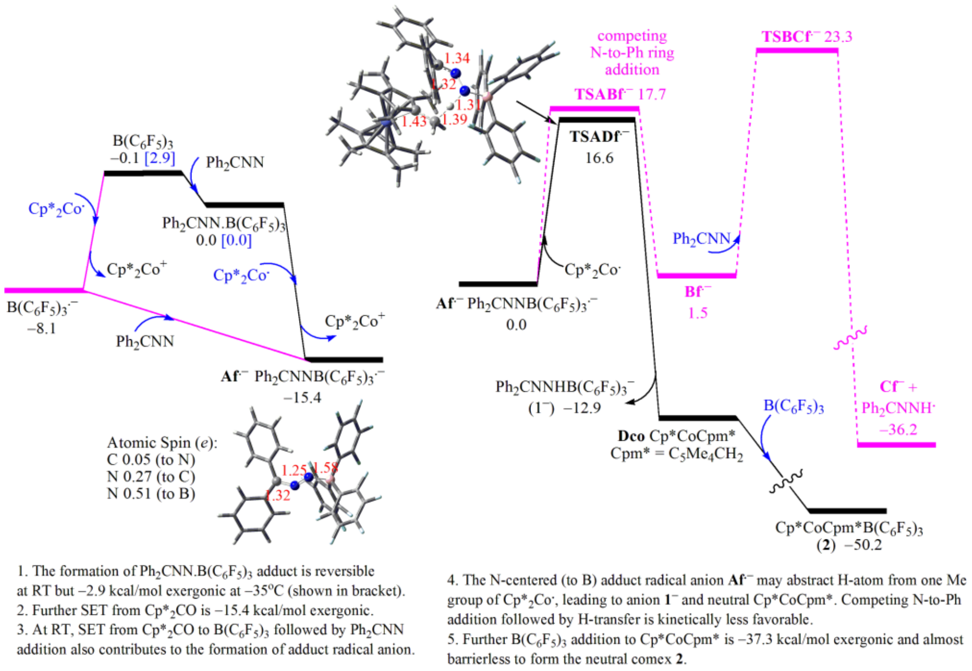

(A)

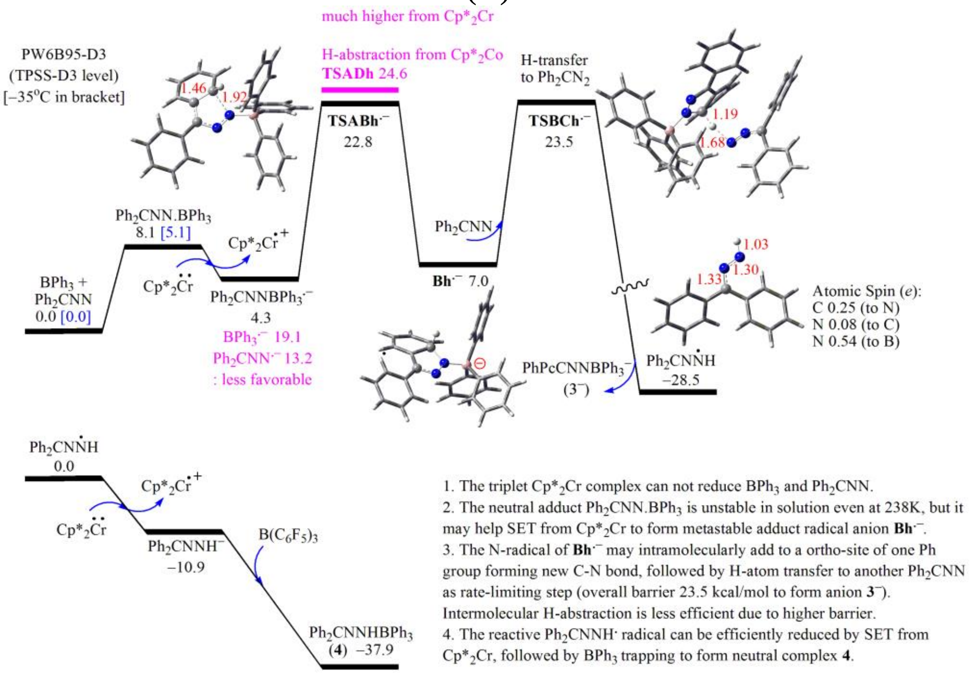

(B)

Figure S31. The DFT-computed reaction free energy paths in PhCl solution (in kcal/mol, at 298 K and 1 mol/L reference concentration) for the reactions of Ph_2CNN with (A) $B(C_6F_5)_3$ and $Cp^*_2Co^·$, and (B) BPh_3 and triplet Cp^*_2Cr, computed at the PW6B95-D3/def2-QZVP + COSMO-RS // TPSS-D3/def2-TZVP + COSMO level of theory. More detailed energies and brief description of potential reactions are given in Tables S1. Selected bond lengths are given in Å; crucial H, C, N and B atoms are highlighted as white, grey, blue and pink balls, respectively.

Table S4. TPSS-D3/def2-TZVP + COSMO computed lowest imaginary frequency (ImF), zero-point energies (ZPE), gas-phase enthalpic (Hc) and Gibbs free-energy (Gc) corrections; the COSMO-RS computed solvation enthalpic (Hsol) and Gibbs free-energy (Gsol) corrections in PhCl solution; TPSS-D3/def2-QZVP and PW6B95-D3/def2-QZVP single-point energies (TPSS-D3 and PW6B95-D3); the relative electronic energies (ΔET and ΔEP) and Gibbs free-energies (ΔGT and ΔGP) at the TPSS-D3 and PW6B95-D3 levels. Each structure is labeled either by its molecular formula or a specific name in bold. Transition structures (with only one imaginary frequency) are indicated by the "TS" suffix while radicals, positive and negative charges are indicated by ˙, + and – signs, respectively. See also Figure S1 for labellings.

Reactions	ImF cm^{-1}	Zpe kcal/mol	Hc kcal/mol	Gc kcal/mol	Hsol kcal/mol	Gsol kcal/mol	TPSS Eh	PW6B95 Eh	ET kcal/mol	EP kcal/mol	GT kcal/mol	GP kcal/mol
*Adduct of BPh_3 (**Rh**) and Ph_2CN_2 (**Rn**): unstable even at 238K (–35 ℃)*												
**Rn** + **Rh** (238K)	0	292.25	304.40	256.56	-35.34	-24.88	-1331.57120	-1333.03099	0.0	0.0	0.0	0.0
**Ah** (238K)	0	293.17	305.42	268.44	-32.68	-24.35	-1331.58415	-1333.03966	-8.1	-5.4	5.1	2.4
**Rn** + **Rh** (298K)	0	292.25	310.46	244.34	-35.34	-24.88	-1331.57120	-1333.03099	0.0	0.0	0.0	0.0
**Ah** (298 K)	0	293.17	311.66	259.19	-32.68	-24.35	-1331.58415	-1333.03966	-8.1	-5.4	8.1	5.4
*Single electron transfer from triplet $Cp^*{}_2Cr$ (**Rcr**) with the ligand Cp* = C_5Me_5, forming quartet $Cp^*{}_2Cr^+$ (**Rcr**$^+$)*												
**Rh** + **Rcr**	0	445.54	472.95	388.69	-36.43	-25.61	-2545.68691	-2547.87482	0.0	0.0	0.0	0.0
**Rh**$^{\cdot-}$ + **Rcr**$^+$	0	446.36	473.37	391.45	-108.22	-94.86	-2545.54636	-2547.73840	88.2	85.6	19.1	21.7
**Rn** + **Rcr**	0	395.11	420.52	339.81	-31.87	-22.10	-2436.64927	-2438.69735	0.0	0.0	0.0	0.0
**Acr** (N...Cr)	0	396.48	422.00	356.60	-30.15	-22.20	-2436.66608	-2438.69990	-10.6	-1.6	13.2	4.3
**Rn**$^{\cdot-}$ + **Rcr**$^+$	0	395.98	421.03	342.17	-107.11	-94.50	-2436.50985	-2438.56463	87.5	83.3	13.3	17.5
**Rn** + **Rh** + **Rcr**	0	566.45	601.97	486.42	-51.82	-36.29	-3156.95369	-3159.80158	0.0	0.0	0.0	0.0
**Ah**$^{\cdot-}$ + **Rcr**$^+$	0	568.68	603.93	504.51	-114.78	-99.07	-3156.86937	-3159.72043	52.9	50.9	4.3	6.3
**Ah**$^{\cdot-}$ + **Rcr**	0	566.95	602.31	501.25	-82.79	-67.84	-3157.04094	-3159.88407	0.0	0.0	0.0	0.0
**Ah**$^{2-}$ + **Rcr**$^+$	95	564.95	600.71	500.29	-209.32	-190.58	-3156.81633	-3159.66754	141.0	135.9	12.2	17.3
Intramolecular N-radical cyclization												
**Ah**$^{\cdot-}$ (**INT1**)	0	292.75	310.81	259.17	-66.31	-56.43	-1331.65845	-1333.11348	0.0	0.0	4.3	0.0
**TSABh**$^{\cdot-}$ (**TS1**)	-341	291.64	309.27	258.68	-65.69	-55.96	-1331.63399	-1333.08404	15.4	18.5	22.8	15.3
**Bh**$^{\cdot-}$ (**INT2**)	0	292.41	310.13	259.33	-66.74	-56.97	-1331.65077	-1333.10869	4.8	3.0	7.0	4.4
*... and subsequent H-atom transfer to Ph_2CN_2 (**Rn**)*												
**Ah**$^{\cdot-}$ + **Rn**	0	413.66	439.82	356.89	-81.70	-67.12	-1942.92523	-1945.04024	0.0	0.0	4.3	0.0
**TSBCh1**$^{\cdot-}$	-151	414.16	439.63	373.93	-74.43	-62.28	-1942.93058	-1945.03663	-3.4	2.3	26.6	16.6

**TSBCh**$^{\cdot-}$ (**TS2**)	-55	413.85	439.36	373.48	-74.51	-62.62	-1942.93431	-1945.04024	-5.7	0.0	23.5	13.5
**Cn**$^{\cdot}$ + **Ch**$^{-}$ (**3**$^{-}$)	0	415.01	440.60	358.40	-84.34	-69.44	-1942.96895	-1945.09128	-27.4	-32.0	-28.5	-28.3
Triplet Cp$_2$Cr (**Rcr**) reduction and BPh$_3$ (**Rh**) trapping of Ph$_2$CNNH (**Cn**)*												
**Cn**$^{\cdot}$ + **Rcr**	0	402.84	428.02	347.45	-33.52	-23.41	-2437.22756	-2439.27468	0.0	0.0	0.0	0.0
Cn^{-} + **Rcr**$^{+}$	0	402.91	428.23	348.83	-109.16	-96.30	-2437.12182	-2439.17805	66.4	60.7	-10.9	-5.2
**Cn**$^{-}$ + **Rh**	0	298.32	316.56	250.10	-80.64	-67.85	-1332.21532	-1333.67533	0.0	0.0	0.0	0.0
**Dh**$^{-}$ (**4**$^{-}$)	19	298.75	317.44	264.23	-66.75	-56.68	-1332.29520	-1333.75574	-50.1	-50.5	-27.1	-26.7
*Reversible Adduct of B(C$_6$F$_5$)$_3$ (**Rf**) and Ph$_2$CN$_2$ (**Rn**): stable at 238K (–35 ℃) in solution*												
**Rn** + **Rf** (238K)	0	215.19	233.38	173.65	-30.96	-22.28	-2820.88910	-2823.80079	0.0	0.0	0.0	0.0
**Af** (238K)	0	216.31	234.56	186.14	-28.43	-21.76	-2820.91192	-2823.82319	-14.3	-14.1	-2.9	-3.2
**Rn** + **Rf** (298K)	0	215.19	242.12	158.38	-30.96	-22.28	-2820.88910	-2823.80079	0.0	0.0	0.0	0.0
**Af** (298K)	0	216.31	243.47	173.95	-28.43	-21.76	-2820.91192	-2823.82319	-14.3	-14.1	0.1	-0.1
Single electron transfer from Cp$_2$Co$^{\cdot}$ (**Rco**$^{\cdot}$): facile formation of adduct radical anion Ph$_2$CNNB(C$_6$F$_5$)$_3^{\cdot-}$ and singlet cation Cp*$_2$Co^{+} (**Rco**$^{+}$):*												
**Af** + **Rco**$^{\cdot}$	0	490.42	534.86	416.57	-44.64	-32.97	-4984.62107	-4989.04730	0.0	0.0	0.0	0.0
**Af**$^{\cdot-}$ + **Rco**$^{+}$	0	492.47	536.10	420.58	-103.41	-90.27	-4984.57682	-4988.98701	27.8	37.8	-15.5	-25.5
**Rf** + **Rco**$^{\cdot}$	0	368.39	404.50	303.27	-31.77	-22.81	-4373.33146	-4377.09814	0.0	0.0	0.0	0.0
**Rf**$^{\cdot-}$ + **Rco**$^{+}$	0	369.94	405.51	306.56	-92.81	-82.44	-4373.27019	-4377.02112	38.5	48.3	-8.0	-17.9
**Rn** + **Rf**$^{\cdot-}$	0	214.33	241.14	158.01	-59.83	-50.46	-2820.97253	-2823.88535	0.0	0.0	0.0	0.0
**Af**$^{\cdot-}$	0	215.96	242.71	174.30	-55.05	-47.60	-2821.01238	-2823.92448	-25.0	-24.6	-7.3	-7.7
**Rn** + **Rco**$^{\cdot}$	0	395.02	420.41	340.35	-31.59	-21.91	-2774.97592	-2777.15087	0.0	0.0	0.0	0.0
**Rn**$^{\cdot-}$ + **Rco**$^{+}$	0	396.56	421.29	343.11	-107.01	-94.52	-2774.86338	-2777.02021	70.6	82.0	12.1	0.8
Intramolecular N-radical cyclization												
**Af**$^{\cdot-}$	0	215.96	242.71	174.30	-55.05	-47.60	-2821.01238	-2823.92448	0.0	0.0	0.0	0.0
**TSABf**$^{\cdot-}$	-326	215.05	241.40	173.76	-54.96	-47.40	-2820.98763	-2823.89572	15.5	18.1	17.7	15.2
**Bf**$^{\cdot-}$	0	215.62	242.04	174.32	-55.20	-47.76	-2821.00512	-2823.92187	4.6	1.6	1.5	4.4
*... and subsequent H-atom transfer to Ph$_2$CN$_2$ (**Rn**)*												
**Bf**$^{\cdot-}$ + **Rn**	0	336.87	371.73	272.03	-70.44	-58.29	-3432.27916	-3435.85124	0.0	0.0	0.0	0.0
**TSBCf**$^{\cdot-}$	-374	335.66	370.19	287.09	-65.79	-55.63	-3432.27402	-3435.83939	3.2	7.4	23.3	19.1
**Cf**$^{-}$ + **Cn**$^{\cdot}$	0	338.42	372.71	273.48	-73.05	-60.45	-3432.32600	-3435.90779	-29.4	-35.5	-36.2	-30.1
**Cn**$^{\cdot}$ + Rco$^{\cdot}$	0	402.75	427.91	347.98	-33.24	-23.22	-2775.55421	-2777.72819	0.0	0.0	0.0	0.0

**Cn**$^-$ + **Rco**$^+$	0	403.49	428.49	349.76	-109.06	-96.33	-2775.47534	-2777.63362	49.5	59.4	-12.0	-21.8
**Cn**$^-$ + **Rf**	0	221.26	248.21	164.14	-76.26	-65.25	-2821.53323	-2824.44512	0.0	0.0	0.0	0.0
**Df**$^-$ (**1**$^-$)	12	222.35	249.76	179.18	-55.19	-47.59	-2821.64846	-2824.56644	-72.3	-76.1	-45.3	-41.5
H-abstraction from Cp$_2$Co$^•$ (**Rco**$^•$): proton-coupled electron transfer (SET to **Af**$^-$ is endergonic from triplet **Rcr** but nearly neutral from Rco$^•$!)*												
**Af**$^{•-}$ + **Rcr**	0	490.16	534.21	416.38	-71.53	-59.01	-4646.39486	-4650.69507	0.0	0.0	0.0	0.0
**Af**$^{2-}$ + **Rcr**$^+$	0	488.23	532.90	414.72	-188.41	-172.83	-4646.19558	-4650.50194	125.1	121.2	5.7	9.6
**Af**$^{•-}$ + **Rco**$^•$	0	490.07	534.10	416.92	-71.25	-58.82	-4984.72152	-4989.14859	0.0	0.0	0.0	0.0
**Af**$^{2-}$ + **Rco**$^+$	0	488.81	533.16	415.66	-188.31	-172.86	-4984.54910	-4988.95752	108.2	119.9	4.6	-7.1
**TSADf**$^-$	-1651	487.57	531.72	430.01	-69.05	-58.98	-4984.73520	-4989.13973	-8.6	5.6	16.6	2.5
**Dco** + **Df**$^-$ (**1**$^-$)	12	489.73	533.83	415.23	-72.82	-59.92	-4984.75039	-4989.16473	-18.1	-10.1	-12.9	-20.9
**Dco** + **Rf**	0	361.66	397.17	296.71	-33.20	-23.92	-4372.72425	-4376.47231	0.0	0.0	0.0	0.0
**TSDEf**	-46	362.06	397.42	312.52	-30.19	-23.24	-4372.74058	-4376.48739	-10.3	-9.5	5.1	4.4
**Ef** (**2**)	0	364.45	399.88	315.03	-36.45	-29.11	-4372.79378	-4376.54972	-43.6	-48.6	-37.3	-32.4
...3.6 kcal/mol higher barrier for H-abstraction from Cp$_2$Co$^•$ (**Rco**$^•$) with N-radical anion **Ah**$^{•-}$*												
**Rco**$^•$ **+ Ah**$^{•-}$ (**INT1**)	0	566.86	602.20	501.78	-82.51	-67.64	-3495.36759	-3498.33758	0.0	0.0	0.0	0.0
**TSADh**$^-$ (**TS1'**)	-1603	563.73	599.21	514.17	-78.83	-66.42	-3495.37906	-3498.32403	-7.2	8.5	20.2	4.5
**Dco** + **Dh**$^-$ (**4**$^-$)	19	566.13	601.51	500.28	-84.37	-69.01	-3495.39713	-3498.35402	-18.5	-10.3	-13.2	-21.4
*Redox potentials relative to the Cp$_2$Fe (**Fc**) in CH_3CN solution (Experimental values: -43.4, -33.4 and -12.2 for **Rco**$^•$, triplet **Rcr** and singlet **Fs**, respectively)*												
**Rco**$^•$ + **Fc**$^{•+}$	0	379.78	403.19	327.65	-62.50	-54.77	-3814.68917	-3817.25186	0.0	0.0	0.0	0.0
**Rco**$^+$ + **Fc**	0	380.95	403.83	331.28	-47.90	-44.04	-3814.78817	-3817.32713	-62.1	-47.2	-32.9	-47.8
**Rcr** + **Fc**$^{•+}$	0	379.88	403.30	327.12	-62.77	-54.95	-3476.36252	-3478.79835	0.0	0.0	0.0	0.0
**Rcr**$^+$ + **Fc**	0	380.37	403.57	330.34	-47.77	-43.83	-3476.43465	-3478.87155	-45.3	-45.9	-31.6	-30.9
**Fs** + **Fc**$^{•+}$	0	381.07	403.97	330.43	-62.38	-54.67	-3695.65763	-3698.16151	0.0	0.0	0.0	0.0
**Fs**$^{•+}$ + **Fc**	0	380.88	403.74	330.40	-47.83	-43.95	-3695.69190	-3698.19543	-21.5	-21.3	-10.6	-10.8
*Redox potentials relative to the Cp$_2$Fe (**Fc**) in PhCl solution: Cp*$_2$Co$^•$ (**Rco**$^•$), triplet Cp*$_2$cr (**Rcr**), Cp*$_2$Fe (**Fs**)*												
**Rco**$^•$ + **Fc**$^{•+}$	0	379.78	403.19	327.65	-68.64	-58.56	-3814.68917	-3817.25186	0.0	0.0	0.0	0.0
**Rco**$^+$ + **Fc**	0	380.95	403.83	331.28	-60.11	-50.73	-3814.78817	-3817.32713	-62.1	-47.2	-35.8	-50.7
**Rcr** + **Fc**$^{•+}$	0	379.88	403.30	327.12	-68.92	-58.75	-3476.36252	-3478.79835	0.0	0.0	0.0	0.0
**Rcr**$^+$ + **Fc**	0	380.37	403.57	330.34	-60.21	-50.71	-3476.43465	-3478.87155	-45.3	-45.9	-34.7	-34.0
**Fs** + **Fc**$^{•+}$	0	381.07	403.97	330.43	-68.46	-58.42	-3695.65763	-3698.16151	0.0	0.0	0.0	0.0
**Fs**$^{•+}$ + **Fc**	0	380.88	403.74	330.40	-48.81	-44.77	-3695.69190	-3698.19543	-21.5	-21.3	-7.7	-7.9

Table S5. The TPSS-D3/def2-TZVP + COSMO optimized atomic Cartesian coordinates (in Å) in PhCl solution. Each structure is labeled by the specific name (See also Figure S1 and Table S1), followed by the number of atoms, the total energy, and the detailed atomic coordinates (in double-column text list).

Acr $Ph_2CNN.CrCp^*_2$ adduct with Cp* = C_5Me_5

```
76
Energy = -2436.565272267
Cr   -1.3212688   -0.2423734    0.0140557
C    -3.9493891   -1.5892018    2.4564204
C     0.1108484   -2.5158754   -1.9415742
C    -3.0006782   -0.5070721    2.0420105
C    -1.0718274   -1.6209830   -1.7368730
C    -0.8467459   -1.7079564    2.9379759
C    -2.5844787   -3.3584967   -0.5393673
C    -1.5633269   -0.5747319    2.2615078
C    -2.2988721   -1.9974379   -1.1014907
C    -3.3006690    0.7521252    1.5185273
C    -1.2241581   -0.2872039   -2.2237132
C    -1.0239984    0.7230424    2.0071179
C    -3.2339432   -0.9329345   -1.2616934
C    -4.6462087    1.3508360    1.2522461
C    -0.2308751    0.4638517   -3.0576529
C    -2.0529393    1.4862492    1.3819011
C    -2.5525071    0.1489696   -1.8922866
C     0.3142303    1.2340083    2.4427701
C    -4.6960449   -1.0162740   -0.9578896
C    -1.9457275    2.9278807    0.9754101
C    -3.1495052    1.4555798   -2.3233608
H    -3.7130345   -2.5509482    1.9883823
H     0.2684459   -3.1752638   -1.0831450
H     0.2215173   -1.6912590    2.7030688
H    -3.5132459   -3.3680867    0.0348724
H    -1.2458646   -2.6773286    2.6223438
H    -1.7742995   -3.7077198    0.1089804
H    -4.9854095   -1.3381247    2.2141824
H     1.0279203   -1.9429016   -2.0935977
H    -5.4493458    0.6210855    1.3772699
H    -0.3697878    0.2259450   -4.1211347
H    -4.7245714    1.7700114    0.2433316
H     0.7972557    0.2051413   -2.7905416
H     1.0459463    0.4281605    2.5336780
H    -4.8919977   -1.3357661    0.0680882
H    -3.8913942   -1.7473972    3.5418477
H    -0.0438497   -3.1484852   -2.8259067
H    -0.9505439   -1.6512673    4.0309020
H    -2.6886769   -4.0909536   -1.3508781
H     0.7161792    1.9749386    1.7470459
H    -5.1932736   -0.0580118   -1.1158177
H    -2.2423257    3.5889835    1.8021286
H    -2.4262883    2.2726018   -2.2446807
H    -2.5976137    3.1582323    0.1272669
H    -4.0254508    1.7199277   -1.7265564
H    -4.8343346    2.1793066    1.9490727
H    -0.3457392    1.5461116   -2.9492797
H     0.2156348    1.7109866    3.4278534
H    -5.1708873   -1.7490187   -1.6234519
H    -0.9198286    3.1850802    0.6983610
H    -3.4682175    1.4022758   -3.3728663
N     0.4374469   -0.2875046    0.0629345
N     1.6248913   -0.5571965    0.0701994
C     2.7820673    0.1101003   -0.0801406
C     2.8198532    1.5604089   -0.2497079
C     3.9731847   -0.7438631   -0.0052077
C     1.6905215    2.2843492   -0.6936119
C     3.9682646    2.3124598    0.0945663
C     3.9406423   -1.9543252    0.7211217
C     5.1671456   -0.4327969   -0.6888700
C     1.7183267    3.6711429   -0.8149686
H     0.7816683    1.7483010   -0.9374280
C     3.9899579    3.6977652   -0.0255156
C     5.0435621   -2.8004316    0.7700259
H     3.0276427   -2.2185442    1.2470317
C     6.2730051   -1.2792101   -0.6337286
C     2.8683863    4.3935620   -0.4896111
H     0.8317989    4.1924887   -1.1685566
H     4.8888064    4.2408709    0.2560040
C     6.2251960   -2.4680964    0.0985136
H     4.9865117   -3.7220943    1.3443511
H     7.1755090   -1.0128499   -1.1785101
H     2.8897198    5.4753767   -0.5853184
H     7.0894604   -3.1248553    0.1424567
H     4.8422229    1.8014076    0.4843919
H     5.2176614    0.4680722   -1.2925701
```

Af^{2-} $Ph_2CNNB(C_6F_5)_3^{2-}$

```
59
Energy = -2820.959203485
B    -0.7650959    0.1857312   -0.0260856
C    -0.3565970    1.7629983   -0.2528061
C    -1.7088597   -0.4588933   -1.2223532
C    -1.5006436   -0.0593951    1.4545886
C    -0.5031306    2.8041958    0.6561561
C     0.4989735    2.0662074   -1.3209358
C    -1.9300684   -1.8430523   -1.2368299
C    -2.2504324    0.2110566   -2.3174354
C    -0.7398405   -0.2265048    2.6151828
C    -2.8706123   -0.2137945    1.6514584
C     0.1406942    4.0350045    0.5538192
F    -1.3376172    2.6858701    1.7397959
C     1.1564242    3.2793445   -1.4717353
F     0.6397379    1.1907951   -2.3547368
C    -2.5607732   -2.5145560   -2.2785630
F    -1.5892634   -2.6130421   -0.1748146
C    -2.8823497   -0.4209388   -3.3866103
F    -2.2192762    1.5746324   -2.3959657
C    -1.2802998   -0.5688764    3.8514879
F     0.5959190   -0.0155806    2.6074889
C    -3.4581096   -0.5589616    2.8658096
F    -3.7487187   -0.0001971    0.6244226
C     0.9885074    4.2751061   -0.5187644
F    -0.0854150    5.0290237    1.4635755
F     1.9851218    3.5028285   -2.5291485
C    -3.0318882   -1.8006530   -3.3754222
F    -2.7448291   -3.8620439   -2.2361287
F    -3.3703169    0.2956160   -4.4370097
C    -2.6529308   -0.7460594    3.9810421
F    -0.4863865   -0.7142624    4.9461382
F    -4.8069655   -0.7046620    2.9831263
F     1.6584273    5.4538132   -0.6233815
F    -3.6275351   -2.4413448   -4.4143205
F    -3.1948984   -1.0914398    5.1762192
N     0.4887321   -0.6911416   -0.0705489
```

N	1.6475889	-0.1301425	-0.0998428
C	2.7876843	-0.8605435	-0.0840490
C	2.8049169	-2.3310340	-0.1163080
C	4.0194559	-0.0826749	-0.0138973
C	1.8257631	-3.1110105	0.5403280
C	3.7771573	-3.0308848	-0.8662181
C	4.0308258	1.3084910	-0.2961927
C	5.2533235	-0.6407313	0.4072021
C	1.8369990	-4.5021840	0.4608240
H	1.0542684	-2.6039578	1.1035726
C	3.7877394	-4.4208160	-0.9420478
H	4.5283113	-2.4628400	-1.4084883
C	5.1818258	2.0742939	-0.1718478
H	3.0936206	1.7670070	-0.5870400
C	6.4062279	0.1310934	0.5234868
H	5.2938056	-1.6926246	0.6738802
C	2.8154695	-5.1735602	-0.2764497
H	1.0709061	-5.0696241	0.9868331
H	4.5539443	-4.9198848	-1.5324298
C	6.3931881	1.4981849	0.2329116
H	5.1377900	3.1385184	-0.4007097
H	7.3271179	-0.3419435	0.8604964
H	2.8206310	-6.2595545	-0.3348236
H	7.2954029	2.0975638	0.3237929

Af$^{\cdot-}$ $Ph_2CNNB(C_6F_5)_3^{\cdot-}$ radical anion

59

Energy = -2820.906581298

B	-0.8515817	0.0626044	0.0058986
C	-0.2302603	-0.5269590	1.4132878
C	-1.3781272	1.6132402	0.2014865
C	-1.9886735	-0.9157500	-0.6849378
C	-0.6778788	-1.6363336	2.1272842
C	0.9126421	0.0741721	1.9503539
C	-1.4355000	2.5228828	-0.8583093
C	-1.7776522	2.1470492	1.4273698
C	-1.6401118	-2.2089876	-1.0928678
C	-3.3037608	-0.5616256	-0.9865671
C	-0.0247580	-2.1540710	3.2447443
F	-1.8239227	-2.2796956	1.7779220
C	1.5995064	-0.4048141	3.0598466
F	1.3977917	1.2169954	1.4009333
C	-1.7875042	3.8618743	-0.7165576
F	-1.1856106	2.1222961	-2.1307222
C	-2.1448053	3.4775986	1.6166161
F	-1.8582646	1.3646347	2.5368524
C	-2.5003469	-3.0773571	-1.7547907
F	-0.4100939	-2.6979455	-0.8123915
C	-4.2002559	-1.3965705	-1.6540858
F	-3.8121844	0.6460338	-0.6218230
C	1.1298909	-1.5395627	3.7121466
F	-0.5082917	-3.2418439	3.8878118
F	2.7057811	0.2212949	3.5187855
C	-2.1397501	4.3500979	0.5363240
F	-1.8101013	4.6890830	-1.7863325
F	-2.5128266	3.9268654	2.8376084
C	-3.7965078	-2.6651729	-2.0457518
F	-2.1009746	-4.3198683	-2.1071577
F	-5.4609829	-0.9848653	-1.9208724
F	1.7761705	-2.0254167	4.7917612
F	-2.4892860	5.6427498	0.6959364
F	-4.6476440	-3.4880127	-2.6917551
N	0.3193280	0.1455953	-1.0503383
N	1.3893523	-0.4808284	-0.8959849
C	2.6583958	-0.2502691	-1.1648531
C	3.1616787	1.0961620	-1.4761387
C	3.5322987	-1.4325559	-1.0462390
C	2.3061886	2.0899102	-1.9923700
C	4.5004102	1.4513025	-1.2093746
C	3.2031630	-2.4642168	-0.1454389
C	4.6752285	-1.5969664	-1.8500179
C	2.7769467	3.3748065	-2.2506560
H	1.2660427	1.8450671	-2.1766725
C	4.9663592	2.7369989	-1.4699847
H	5.1726857	0.7169770	-0.7774288
C	3.9854248	-3.6102883	-0.0518310
H	2.3214685	-2.3454047	0.4766940
C	5.4610433	-2.7445008	-1.7505650
H	4.9361752	-0.8296217	-2.5728818
C	4.1096829	3.7083302	-1.9962600
H	2.0953949	4.1225987	-2.6486326
H	6.0019133	2.9850745	-1.2506828
C	5.1244159	-3.7574288	-0.8501260
H	3.7132415	-4.3898468	0.6553881
H	6.3358731	-2.8504244	-2.3873488
H	4.4730552	4.7130259	-2.1943911
H	5.7405396	-4.6489172	-0.7702703

Af $Ph_2CNNB(C_6F_5)_3$ adduct

59

Energy = -2820.778288281

B	-0.9369303	0.0172616	-0.1387546
C	-0.0624368	-0.4518710	1.1594056
C	-1.4464274	1.5515855	0.0536424
C	-2.0759084	-1.0205796	-0.6739531
C	-0.2369952	-1.6232124	1.9018641
C	1.0213825	0.3342963	1.5698921
C	-1.2718137	2.6057682	-0.8383957
C	-2.0962057	1.8910017	1.2431386
C	-1.7038994	-2.2929826	-1.1172328
C	-3.4432920	-0.7527794	-0.7778996
C	0.6330931	-2.0253484	2.9137536
F	-1.2878251	-2.4436834	1.6750236
C	1.9113797	-0.0242804	2.5763305
F	1.2557152	1.5295091	0.9671366
C	-1.6743213	3.9127473	-0.5710171
F	-0.6725273	2.4027407	-2.0409373
C	-2.5266340	3.1763598	1.5475048
F	-2.3637541	0.9280910	2.1589374
C	-2.5894275	-3.2469232	-1.5997059
F	-0.3955050	-2.6591193	-1.0665785
C	-4.3704162	-1.6789751	-1.2552321
F	-3.9585122	0.4431323	-0.4098260
C	1.7211449	-1.2259488	3.2496138
F	0.4306132	-3.1813074	3.5725367
F	2.9452938	0.7733020	2.9006501
C	-2.3060484	4.2021619	0.6327457
F	-1.4575751	4.8948099	-1.4671280
F	-3.1603481	3.4368909	2.7062709
C	-3.9441159	-2.9352218	-1.6674884
F	-2.1585372	-4.4598042	-1.9940062
F	-5.6771077	-1.3627542	-1.3260664
F	2.5672035	-1.6022988	4.2201084
F	-2.7071467	5.4541999	0.9072663
F	-4.8258430	-3.8354490	-2.1287969
N	0.1171667	0.0166248	-1.4239238
N	1.2862957	-0.0493540	-1.3299596
C	2.5693471	-0.0711637	-1.3025258
C	3.2651224	1.2284193	-1.4278373
C	3.2634846	-1.3612558	-1.1154388
C	2.7403614	2.2439516	-2.2395599
C	4.4426492	1.4492113	-0.6980877
C	2.7026764	-2.3720563	-0.3208328
C	4.5129602	-1.5628532	-1.7223643

C	3.3933291	3.4697232	-2.3240823
H	1.8336547	2.0684434	-2.8096614
C	5.0903204	2.6781908	-0.7910289
H	4.8354493	0.6692330	-0.0539513
C	3.3833001	-3.5710796	-0.1361512
H	1.7456269	-2.2073310	0.1610986
C	5.1894355	-2.7646810	-1.5284459
H	4.9452173	-0.7850972	-2.3435059
C	4.5684029	3.6891793	-1.6015135
H	2.9879167	4.2513805	-2.9588731
H	5.9999925	2.8488755	-0.2237724
C	4.6287064	-3.7696566	-0.7365734
H	2.9472804	-4.3460945	0.4868285
H	6.1560913	-2.9165964	-1.9985190
H	5.0766270	4.6462221	-1.6700714
H	5.1623282	-4.7028376	-0.5842964

Ah^{2-} $Ph_2CNNBPh_3^{2-}$ with Ph = C_6H_5
59
Energy = -1331.681190069

B	-0.6644801	0.3106597	0.2369942
C	-0.2929180	-0.3782185	1.6736688
C	-1.4592729	1.7193624	0.4739169
C	-1.5897985	-0.7165685	-0.6449063
C	-0.9308721	-1.5075239	2.2126496
C	0.7416544	0.1765487	2.4528608
C	-1.4009109	2.7327538	-0.5031385
C	-2.2560015	1.9897413	1.5998795
C	-1.0263435	-1.9176913	-1.1249055
C	-2.9490556	-0.5081597	-0.9395526
C	-0.5696041	-2.0542080	3.4502682
H	-1.7241249	-1.9831529	1.6373097
C	1.1145991	-0.3497719	3.6888551
H	1.2762235	1.0346893	2.0503931
C	-2.0980885	3.9361465	-0.3726573
H	-0.7766319	2.5555268	-1.3762483
C	-2.9608700	3.1894295	1.7493215
H	-2.3180487	1.2373086	2.3860572
C	-1.7717248	-2.8534112	-1.8422520
H	0.0288487	-2.0972461	-0.9254048
C	-3.7128170	-1.4408106	-1.6511277
H	-3.4261843	0.4068578	-0.5908911
C	0.4574848	-1.4762837	4.1987408
H	-1.0863095	-2.9361345	3.8290818
H	1.9233059	0.1091179	4.2575358
C	-2.8858755	4.1736050	0.7596245
H	-2.0260784	4.6975479	-1.1493839
H	-3.5652288	3.3613920	2.6397323
C	-3.1284132	-2.6254700	-2.1069745
H	-1.2993149	-3.7701272	-2.1951422
H	-4.7672700	-1.2462409	-1.8479672
H	0.7473948	-1.8981695	5.1594175
H	-3.4294167	5.1108012	0.8685281
H	-3.7167150	-3.3574828	-2.6577699
N	0.6393532	0.6676485	-0.5643926
N	1.5073696	-0.2717119	-0.5679320
C	2.7499047	-0.1522132	-1.1654218
C	3.3009318	1.0818948	-1.6943997
C	3.4889415	-1.4034176	-1.1390647
C	2.4701302	2.1584142	-2.1277122
C	4.7021811	1.3410442	-1.7183370
C	3.1151961	-2.4511982	-0.2443981
C	4.5589601	-1.7099385	-2.0260127
C	3.0089879	3.3669494	-2.5646457
H	1.3989201	2.0140344	-2.0678696
C	5.2314490	2.5464438	-2.1648034
H	5.3828480	0.5781218	-1.3508446
C	3.7610345	-3.6800963	-0.2408964
H	2.2948230	-2.2529403	0.4380290
C	5.2060534	-2.9434551	-2.0119019
H	4.8594191	-0.9727312	-2.7652397
C	4.3916823	3.5830817	-2.6031616
H	2.3314477	4.1576675	-2.8869950
H	6.3121246	2.6869478	-2.1613482
C	4.8252101	-3.9516630	-1.1176177
H	3.4393157	-4.4425888	0.4681010
H	6.0121537	-3.1253330	-2.7225611
H	4.8042397	4.5263243	-2.9542067
H	5.3345278	-4.9126597	-1.1050802

Ah$^{\cdot-}$ $Ph_2CNNBPh_3^{\cdot-}$ radical anion
59
Energy = -1331.629800517

B	-0.7930921	0.1804442	0.0204556
C	-0.2201038	-0.4027230	1.4283049
C	-1.4512926	1.6558849	0.1967458
C	-1.8367473	-0.8656124	-0.6784767
C	-0.8309459	-1.4563630	2.1275419
C	0.9431992	0.1410174	2.0069935
C	-1.4673607	2.5917529	-0.8528784
C	-2.0822600	2.0423135	1.3913395
C	-1.3805906	-2.1105914	-1.1519445
C	-3.2112614	-0.6071451	-0.8065911
C	-0.3129761	-1.9519894	3.3288326
H	-1.7284694	-1.9115303	1.7132919
C	1.4798857	-0.3490362	3.1975722
H	1.4492234	0.9613856	1.5005773
C	-2.0719181	3.8439453	-0.7225216
H	-0.9893516	2.3281386	-1.7942996
C	-2.7023352	3.2867657	1.5349119
H	-2.0816332	1.3497282	2.2314334
C	-2.2440477	-3.0471920	-1.7210816
H	-0.3208356	-2.3459706	-1.0705883
C	-4.0907435	-1.5392093	-1.3686274
H	-3.6014199	0.3464805	-0.4559759
C	0.8518463	-1.4039169	3.8675064
H	-0.8139654	-2.7725093	3.8398131
H	2.3907595	0.0866457	3.6043247
C	-2.6972049	4.1980077	0.4763568
H	-2.0575888	4.5459954	-1.5546144
H	-3.1824750	3.5508667	2.4756332
C	-3.6098919	-2.7654013	-1.8315344
H	-1.8556489	-3.9988733	-2.0794636
H	-5.1511838	-1.3063848	-1.4490562
H	1.2677129	-1.7932488	4.7941305
H	-3.1702070	5.1715429	0.5846374
H	-4.2877456	-3.4917586	-2.2740725
N	0.4104490	0.3491079	-1.0143422
N	1.4313030	-0.3576291	-0.9454887
C	2.7196164	-0.1784589	-1.2106105
C	3.2920279	1.1502062	-1.4363105
C	3.5134676	-1.4199501	-1.1504350
C	2.4687331	2.2661354	-1.7116322
C	4.6841255	1.3825635	-1.3388918
C	3.1508164	-2.4328765	-0.2388544
C	4.6054049	-1.6652251	-2.0043560
C	3.0139629	3.5313492	-1.9169914
H	1.3930606	2.1266702	-1.7460526
C	5.2218485	2.6490357	-1.5451378
H	5.3454528	0.5614708	-1.0833353
C	3.8472037	-3.6365830	-0.1890371
H	2.3186712	-2.2472897	0.4341863
C	5.3085998	-2.8683773	-1.9445224
H	4.8885142	-0.9171291	-2.7390412

C	4.3940965	3.7359202	-1.8455761
H	2.3510034	4.3668724	-2.1297488
H	6.2970524	2.7908082	-1.4630771
C	4.9364496	-3.8629002	-1.0372047
H	3.5493682	-4.3990260	0.5269907
H	6.1450065	-3.0331088	-2.6196009
H	4.8166318	4.7241162	-2.0061805
H	5.4861511	-4.7993950	-0.9911788

Ah $Ph_2CNN.BPh_3$ adduct (**INT1** in text)
59
Energy = -1331.520153428

B	-0.9429423	-0.0937182	-0.1382554
C	-0.0633409	-0.5631053	1.1239515
C	-1.2973916	1.4842269	-0.2407666
C	-2.1493071	-1.0695698	-0.5504926
C	-0.2900580	-1.7917171	1.7706984
C	1.0450910	0.1883578	1.5657475
C	-1.7170492	2.0457100	-1.4623509
C	-1.2288077	2.3423218	0.8688612
C	-1.9240889	-2.3301038	-1.1323768
C	-3.4837718	-0.7170625	-0.2902168
C	0.5462737	-2.2550669	2.7891747
H	-1.1362973	-2.4004366	1.4624271
C	1.8938409	-0.2680413	2.5745637
H	1.2536801	1.1525959	1.1076618
C	-2.0290533	3.4000808	-1.5790378
H	-1.8012760	1.4052099	-2.3377166
C	-1.5556580	3.6967123	0.7667038
H	-0.9246114	1.9432332	1.8328153
C	-2.9731414	-3.1971026	-1.4430706
H	-0.9035510	-2.6455387	-1.3468624
C	-4.5422081	-1.5797726	-0.5837019
H	-3.6971599	0.2555714	0.1482475
C	1.6494433	-1.4980569	3.1884579
H	0.3411440	-3.2098677	3.2672766
H	2.7474336	0.3329359	2.8784042
C	-1.9460501	4.2337053	-0.4609636
H	-2.3415941	3.8055101	-2.5383130
H	-1.5025116	4.3336632	1.6464578
C	-4.2902953	-2.8244253	-1.1649252
H	-2.7655193	-4.1627986	-1.8979320
H	-5.5644307	-1.2789804	-0.3662648
H	2.3111954	-1.8618567	3.9699446
H	-2.1896966	5.2895875	-0.5449479
H	-5.1115648	-3.4960320	-1.4013553
N	0.1289108	-0.1629506	-1.4751899
N	1.2936319	-0.1571241	-1.3729168
C	2.5664971	-0.0483034	-1.2228082
C	3.1167358	1.3146597	-1.1059560
C	3.3452481	-1.3050785	-1.1355025
C	2.3398875	2.4374105	-1.4421450
C	4.4073197	1.5009294	-0.5858521
C	2.8800953	-2.3567464	-0.3325031
C	4.5395798	-1.4501822	-1.8561329
C	2.8523722	3.7182963	-1.2702011
H	1.3334182	2.3119699	-1.8306147
C	4.9144801	2.7886026	-0.4233495
H	5.0023203	0.6420980	-0.2950617
C	3.6060775	-3.5428517	-0.2541294
H	1.9694981	-2.2291857	0.2459459
C	5.2619796	-2.6381948	-1.7675880
H	4.8915838	-0.6417606	-2.4893545
C	4.1426609	3.9003615	-0.7643016
H	2.2414246	4.5772350	-1.5308327
H	5.9141033	2.9208418	-0.0202191
C	4.7972302	-3.6860944	-0.9687177
H	3.2465065	-4.3517254	0.3749297
H	6.1850703	-2.7478410	-2.3288825
H	4.5399546	4.9020454	-0.6313875
H	5.3645381	-4.6096600	-0.9012027

Bf$^{\cdot -}$ (cyclized **Af**$^{\cdot -}$)
59
Energy = -2820.899909686

B	1.3302937	0.1543458	-0.0384461
C	1.9720784	-0.0280099	1.4782446
C	2.1421784	-0.7053231	-1.2005835
C	1.3327826	1.7908090	-0.2784346
C	3.2787530	0.4017110	1.7175790
C	1.2957426	-0.4691051	2.6160157
C	1.7190891	-0.5935411	-2.5304378
C	3.1641528	-1.6351981	-1.0134982
C	0.6012652	2.6139516	0.5868591
C	2.0561220	2.4906336	-1.2446949
C	3.9011596	0.3670636	2.9587033
F	4.0222628	0.8796274	0.6829905
C	1.8802899	-0.5264950	3.8817828
F	-0.0003124	-0.8588599	2.5640326
C	2.2876160	-1.2802036	-3.5957742
F	0.7013382	0.2475175	-2.8430585
C	3.7701205	-2.3452237	-2.0501356
F	3.6353969	-1.9315618	0.2269431
C	0.5184109	3.9968760	0.4616130
F	-0.0532383	2.0903540	1.6505537
C	2.0099918	3.8754323	-1.4013459
F	2.8933826	1.8519010	-2.1030744
C	3.1927674	-0.1091522	4.0576909
F	5.1791997	0.7811382	3.1133588
F	1.1759214	-0.9736390	4.9462048
C	3.3370255	-2.1608951	-3.3553834
F	1.8447133	-1.1002262	-4.8597399
F	4.7707930	-3.2166685	-1.7951130
C	1.2230269	4.6386755	-0.5502509
F	-0.2264487	4.7269283	1.3228008
F	2.7349178	4.4842316	-2.3674543
F	3.7714221	-0.1586092	5.2752044
F	3.9149609	-2.8315795	-4.3708163
F	1.1631964	5.9793305	-0.6859219
N	-0.1237244	-0.3731052	-0.1288465
N	-1.1905026	0.4149116	-0.1920165
C	-2.3159159	-0.3210849	-0.2803092
C	-2.0143532	-1.7088327	-0.2533020
C	-3.6081322	0.3612710	-0.2763814
C	-0.5154969	-1.7795458	-0.3666431
C	-2.7466312	-2.8614126	0.0317820
C	-3.7282336	1.6552719	0.2688952
C	-4.7592306	-0.2476042	-0.8071501
C	0.1006600	-2.8939385	0.4209932
C	-2.0776291	-4.0127155	0.4512172
H	-3.8328765	-2.8416629	0.0307519
C	-4.9561067	2.3095373	0.2827921
H	-2.8426163	2.1285117	0.6813538
C	-5.9888423	0.4104619	-0.7883546
H	-4.6840497	-1.2313813	-1.2608942
C	-0.6704060	-3.9766840	0.7193918
H	1.1585392	-2.8591411	0.6580216
H	-2.6438617	-4.9035610	0.7077202
C	-6.0961365	1.6913629	-0.2430501
H	-5.0285681	3.3055380	0.7118437
H	-6.8647600	-0.0759256	-1.2096650
H	-0.2203082	-4.8294588	1.2232731
H	-7.0541855	2.2030047	-0.2272866
H	-0.2592688	-1.9650664	-1.4419395

Bh$^{\bullet-}$ (INT2 in text**)**

59

Energy = -1331.622109290

B	1.3142764	0.2404856	-0.0845892
C	1.9116557	-0.0104844	1.4172087
C	2.1111606	-0.5791583	-1.2575186
C	1.3639526	1.8542699	-0.3267810
C	3.2484373	0.3145477	1.7184295
C	1.1249667	-0.4660505	2.4883104
C	1.6634323	-0.4624018	-2.5885475
C	3.1903065	-1.4484980	-1.0390460
C	0.5755495	2.7362613	0.4392918
C	2.2884766	2.4482957	-1.2025004
C	3.7776605	0.1744190	3.0020515
H	3.8862969	0.7025514	0.9254746
C	1.6382333	-0.6089655	3.7807640
H	0.0823406	-0.7168757	2.3064454
C	2.2535230	-1.1644624	-3.6396999
H	0.8250629	0.2008738	-2.7991138
C	3.7964688	-2.1595338	-2.0808526
H	3.5624538	-1.5821925	-0.0251981
C	0.6826111	4.1212304	0.3200574
H	-0.1399556	2.3221570	1.1433679
C	2.4201700	3.8367306	-1.3206249
H	2.9259079	1.8073640	-1.8079023
C	2.9716614	-0.2927288	4.0453550
H	4.8167162	0.4357379	3.1948349
H	0.9954384	-0.9673665	4.5828699
C	3.3302784	-2.0210714	-3.3886833
H	1.8756117	-1.0483771	-4.6537303
H	4.6291282	-2.8279176	-1.8695496
C	1.6107158	4.6832930	-0.5631124
H	0.0477857	4.7688636	0.9222227
H	3.1533638	4.2562748	-2.0073892
H	3.3773676	-0.4023408	5.0485490
H	3.7947007	-2.5745185	-4.2017506
H	1.7022219	5.7631911	-0.6547387
N	-0.1674201	-0.3102513	-0.1397790
N	-1.2610380	0.4256622	-0.1684275
C	-2.3710265	-0.3540524	-0.2160581
C	-2.0144602	-1.7281635	-0.1754948
C	-3.6774331	0.2928972	-0.2079019
C	-0.5177569	-1.7349008	-0.3450073
C	-2.6795862	-2.9131658	0.1452318
C	-3.7851454	1.6686200	0.0897390
C	-4.8624237	-0.4113441	-0.5009625
C	0.1793355	-2.8064656	0.4299508
C	-1.9390029	-4.0293575	0.5456705
H	-3.7637927	-2.9526945	0.1905616
C	-5.0218460	2.3041969	0.1032253
H	-2.8750132	2.2194386	0.3059582
C	-6.1015395	0.2289974	-0.4805336
H	-4.8121153	-1.4609104	-0.7697703
C	-0.5272720	-3.9238497	0.7618667
H	1.2431031	-2.7146664	0.6131963
H	-2.4522207	-4.9447592	0.8265569
C	-6.1930740	1.5885034	-0.1757524
H	-5.0780574	3.3646111	0.3359797
H	-6.9998284	-0.3377855	-0.7125480
H	-0.0157152	-4.7524030	1.2481805
H	-7.1586662	2.0859780	-0.1601276
H	-0.2814604	-1.9129122	-1.4254861

Cf$^-$

58

Energy = -2820.376796892

B	1.3183512	0.1565454	0.0618419
C	2.0034721	-0.0162137	1.5577859
C	2.0763056	-0.7103884	-1.1287794
C	1.3214933	1.7871390	-0.1967834
C	3.3223009	0.4063260	1.7445807
C	1.3659521	-0.4249928	2.7300044
C	1.5956570	-0.5944359	-2.4378070
C	3.0908929	-1.6549330	-0.9841777
C	0.5973946	2.6213910	0.6634375
C	2.0430670	2.4736294	-1.1741093
C	3.9827809	0.4080428	2.9665408
F	4.0377949	0.8490276	0.6769156
C	1.9888802	-0.4444765	3.9786635
F	0.0692347	-0.8165221	2.7355648
C	2.0939696	-1.3054265	-3.5220585
F	0.5899241	0.2710889	-2.7100643
C	3.6207286	-2.3976217	-2.0393970
F	3.6138410	-1.9492850	0.2371917
C	0.5209460	4.0029290	0.5255586
F	-0.0608955	2.1067674	1.7289966
C	2.0029055	3.8570545	-1.3434616
F	2.8669443	1.8237517	-2.0360945
C	3.3065627	-0.0266423	4.1022982
F	5.2661808	0.8213105	3.0669343
F	1.3137402	-0.8538067	5.0763846
C	3.1248283	-2.2179309	-3.3225906
F	1.5965607	-1.1210618	-4.7650872
F	4.6001763	-3.3035627	-1.8211008
C	1.2259304	4.6314056	-0.4941999
F	-0.2243884	4.7444971	1.3761662
F	2.7207623	4.4524957	-2.3229677
F	3.9190517	-0.0357994	5.3035349
F	3.6240490	-2.9239539	-4.3565069
F	1.1716071	5.9706168	-0.6418666
N	-0.1582541	-0.3596418	0.0161263
N	-1.2164141	0.4702624	-0.1534527
C	-2.3316880	-0.2671080	-0.1990337
C	-2.0020929	-1.6579559	-0.0690050
C	-3.6324940	0.3951356	-0.2983198
C	-0.5857119	-1.6646907	0.0686486
C	-2.7084106	-2.8739543	0.0011048
C	-3.7618675	1.7430512	0.0911593
C	-4.7807488	-0.2765364	-0.7546054
C	0.1306800	-2.8547300	0.2719770
C	-1.9964291	-4.0508407	0.1845890
H	-3.7908415	-2.9017376	-0.0710429
C	-4.9923974	2.3897190	0.0283942
H	-2.8792592	2.2623166	0.4520122
C	-6.0152596	0.3716042	-0.8062779
H	-4.7060765	-1.3055554	-1.0900454
C	-0.5897107	-4.0385911	0.3178446
H	1.2048949	-2.8533932	0.4139098
H	-2.5272612	-4.9974883	0.2391808
C	-6.1305120	1.7068199	-0.4147672
H	-5.0696763	3.4288422	0.3383141
H	-6.8906935	-0.1708715	-1.1542006
H	-0.0632486	-4.9762967	0.4736008
H	-7.0933392	2.2090611	-0.4516967

Ch$^-$ (**3$^-$** in text)

58

Energy = -1331.096611578

B	-0.4254325	0.2716553	0.1741873
C	-0.2204987	0.0773981	1.7831965
C	-1.1719221	1.6839433	-0.1550113
C	-1.2581566	-0.9866857	-0.4718997
C	-1.1891232	-0.5615021	2.5770850

C	0.9017642	0.5874994	2.4612310
C	-1.2895215	2.1614705	-1.4758928
C	-1.7335042	2.4899200	0.8479798
C	-0.8604771	-2.3183477	-0.2315869
C	-2.4017742	-0.8216289	-1.2707709
C	-1.0545870	-0.6883914	3.9623409
H	-2.0690972	-0.9799152	2.0923261
C	1.0540894	0.4681803	3.8445463
H	1.6834317	1.0841230	1.8894037
C	-1.9111361	3.3725926	-1.7788476
H	-0.8561771	1.5770108	-2.2856041
C	-2.3589958	3.7081002	0.5633015
H	-1.6599512	2.1647468	1.8834617
C	-1.5279551	-3.4083865	-0.7902045
H	0.0065400	-2.4948190	0.3968470
C	-3.0876689	-1.9039370	-1.8344258
H	-2.7705579	0.1824398	-1.4654064
C	0.0723877	-0.1716219	4.6058193
H	-1.8261621	-1.1958330	4.5395524
H	1.9409693	0.8707347	4.3310343
C	-2.4486337	4.1585718	-0.7546327
H	-1.9694285	3.7126627	-2.8109847
H	-2.7669043	4.3105348	1.3735532
C	-2.6452114	-3.2067068	-1.6073219
H	-1.1797519	-4.4205614	-0.5903591
H	-3.9676814	-1.7272812	-2.4507200
H	0.1883041	-0.2722320	5.6826652
H	-2.9243398	5.1101344	-0.9817921
H	-3.1646844	-4.0523979	-2.0531112
N	1.0368586	0.3024866	-0.4866214
N	1.6883785	-0.8665923	-0.6688310
C	2.9370240	-0.6214008	-1.0946000
C	3.1283828	0.7945649	-1.2113097
C	3.8511176	-1.7485231	-1.2922184
C	1.8829624	1.3493062	-0.7763866
C	4.1631644	1.6595970	-1.6236736
C	3.3822719	-3.0714373	-1.1357509
C	5.2145885	-1.5735163	-1.5933308
C	1.7040974	2.7426624	-0.6731945
C	3.9625554	3.0300989	-1.5559630
H	5.1062607	1.2783273	-2.0004921
C	4.2415433	-4.1585783	-1.2603585
H	2.3322110	-3.2205109	-0.9039609
C	6.0729128	-2.6661994	-1.7235070
H	5.6203020	-0.5754367	-1.7068224
C	2.7484546	3.5628175	-1.0673640
H	0.7814346	3.1617462	-0.2956866
H	4.7514682	3.7066265	-1.8739068
C	5.5974959	-3.9668720	-1.5524567
H	3.8549454	-5.1660465	-1.1247995
H	7.1217347	-2.4934496	-1.9524102
H	2.6302529	4.6415351	-1.0004898
H	6.2715263	-4.8154999	-1.6394653

Cn$^-$ Ph$_2$CNNH$^-$

26

Energy = -611.9337590283

N	0.2973065	0.7198764	-1.5406591
N	1.1169210	-0.2854097	-1.3295770
C	2.4545848	-0.1068523	-1.3020228
C	3.1297919	1.1911647	-1.4597883
C	3.2200951	-1.3354829	-1.0804869
C	2.6234653	2.2092231	-2.3020178
C	4.3054347	1.4970508	-0.7344921
C	2.6065405	-2.4927908	-0.5338520
C	4.5845880	-1.4674269	-1.4415926
C	3.2696881	3.4390453	-2.4245538
H	1.7096209	2.0210976	-2.8505747
C	4.9469960	2.7265614	-0.8542544
H	4.7136000	0.7518000	-0.0575527
C	3.3013099	-3.6858202	-0.3706947
H	1.5606790	-2.4247381	-0.2513128
C	5.2803229	-2.6622528	-1.2682816
H	5.1002717	-0.6218716	-1.8869785
C	4.4370213	3.7129349	-1.7058790
H	2.8551464	4.1942295	-3.0899506
H	5.8473186	2.9193155	-0.2740481
C	4.6517637	-3.7900662	-0.7312675
H	2.7875060	-4.5460708	0.0549377
H	6.3251570	-2.7161626	-1.5687309
H	4.9329644	4.6761227	-1.7953402
H	5.1948301	-4.7221729	-0.5990443
H	-0.6384055	0.2926075	-1.4696229

Cn$^{\cdot}$ Ph$_2$CNNH$^{\cdot}$

26

Energy = -611.8183764767

N	0.3418236	0.7377854	-1.2785209
N	1.1588316	-0.2534635	-1.4526547
C	2.4780406	-0.1317077	-1.3491487
C	3.1451308	1.1739865	-1.4846227
C	3.2342013	-1.3700626	-1.1243138
C	2.6270891	2.1734522	-2.3292176
C	4.2965437	1.4656929	-0.7271149
C	2.6216133	-2.4610819	-0.4765179
C	4.5652570	-1.5086920	-1.5601339
C	3.2383135	3.4212867	-2.4120470
H	1.7573551	1.9594629	-2.9404065
C	4.8979382	2.7188975	-0.8034864
H	4.6977137	0.7149079	-0.0539053
C	3.3203164	-3.6439687	-0.2635631
H	1.5944126	-2.3627921	-0.1393061
C	5.2616762	-2.6966381	-1.3481812
H	5.0448053	-0.6895749	-2.0868793
C	4.3743704	3.7006825	-1.6484953
H	2.8286240	4.1768013	-3.0763748
H	5.7738407	2.9312792	-0.1971455
C	4.6453021	-3.7668639	-0.6960334
H	2.8349191	-4.4725039	0.2445577
H	6.2848110	-2.7902481	-1.7011330
H	4.8486607	4.6758765	-1.7109447
H	5.1906387	-4.6910389	-0.5280230
H	-0.5285728	0.5017288	-1.7795297

Dco Cp*CoC$_5$(=CH$_2$)Me$_4$

50

Energy = -2163.024531970

Co	0.0235156	-0.0760027	-0.0980577
C	-0.0831927	-1.9668568	-2.7137622
C	2.7883156	0.7260175	-1.3319673
C	-0.3721951	-1.7423453	-1.2607564
C	1.5633211	1.1004039	-0.5565282
C	1.8390622	-2.7161179	-0.2633630
C	0.0424861	1.8743041	-2.5472836
C	0.4898398	-2.0733746	-0.1634974
C	0.3322171	1.6157063	-1.1011654
C	-1.5771468	-1.1777465	-0.7148433
C	1.4186463	1.0750144	0.8768070
C	-0.2058725	-1.7536176	1.0659097
C	-0.5411938	1.8951141	0.0109626
C	-2.7688232	-0.7335786	-1.5060123
C	2.4788974	0.6539514	1.8449714
C	-1.4814628	-1.2100199	0.7302832
C	0.2042217	1.7978914	1.2689006

C	0.2893268	-1.9698460	2.4614900
C	-1.9153226	2.4796465	-0.0861132
C	-2.4882935	-0.7197097	1.7231560
C	-0.2146643	2.1371984	2.5263960
H	0.9885369	-1.8945108	-2.9230709
H	2.5320536	0.3503222	-2.3271540
H	2.4870691	-2.4160990	0.5654434
H	-1.0233131	1.7629313	-2.7672772
H	2.3400562	-2.4485856	-1.1980755
H	0.5962004	1.1872917	-3.1937031
H	-0.5995205	-1.2335028	-3.3400536
H	3.3660232	-0.0459842	-0.8159443
H	-2.4737986	-0.2979240	-2.4652570
H	3.1016029	1.5151221	2.1275276
H	-3.3558884	0.0109985	-0.9623749
H	3.1396535	-0.1083863	1.4221544
H	1.3745370	-2.0959690	2.4893698
H	-2.3744090	2.2850900	-1.0587115
H	-0.4147910	-2.9645258	-3.0331019
H	3.4448794	1.5970714	-1.4637630
H	1.7537872	-3.8110388	-0.2349319
H	0.3309375	2.8977697	-2.8236961
H	0.0250622	-1.1232983	3.1052171
H	-2.5714182	2.0790946	0.6932924
H	-2.8540040	-1.5394690	2.3542735
H	0.3910467	1.9161903	3.3997901
H	-3.3520687	-0.2700501	1.2265344
H	-1.1881785	2.5880567	2.6912450
H	-3.4295535	-1.5846761	-1.7200558
H	2.0396122	0.2575296	2.7649799
H	-0.1618207	-2.8719769	2.8961146
H	-1.8757193	3.5697101	0.0531793
H	-2.0413182	0.0385586	2.3831089

Dco$^+$ Cp*CoC$_5$(=CH$_2$)Me$_4$$^+$ (singlet)
50
Energy = -2162.876969460

Co	-0.0092597	-0.0318889	0.0177430
C	-0.0292886	-1.8439950	-2.6443679
C	2.7691652	0.6968232	-1.4131918
C	-0.3513768	-1.6634337	-1.1961522
C	1.5783846	1.1069412	-0.6089460
C	1.8527940	-2.6368102	-0.1875097
C	0.0377357	1.8366917	-2.5906488
C	0.4946258	-2.0209456	-0.0900258
C	0.3401637	1.6237189	-1.1427943
C	-1.5879779	-1.1557252	-0.6616881
C	1.4899311	1.1108047	0.8190273
C	-0.2213826	-1.7406824	1.1286573
C	-0.5211094	1.9489904	-0.0475507
C	-2.7708928	-0.7162086	-1.4623067
C	2.5661545	0.7157017	1.7758559
C	-1.5107494	-1.2114381	0.7745374
C	0.2063311	1.6956278	1.1916071
C	0.2606814	-2.0014082	2.5190395
C	-1.8777131	2.5669962	-0.1366505
C	-2.5840645	-0.8180421	1.7368980
C	-0.2670165	1.9053761	2.4906338
H	1.0400882	-1.7290757	-2.8387932
H	2.4797916	0.2662470	-2.3748346
H	2.4894028	-2.3490432	0.6528369
H	-1.0306823	1.7391773	-2.7978639
H	2.3548871	-2.3598038	-1.1170962
H	0.5829298	1.1337911	-3.2241828
H	-0.5757602	-1.1347181	-3.2700143
H	3.3862629	-0.0271213	-0.8767288
H	-2.4715906	-0.2616173	-2.4099478
H	3.1986226	1.5838774	2.0008893
H	-3.3879403	-0.0023719	-0.9122622
H	3.2104362	-0.0616940	1.3581827
H	1.3511638	-2.0380910	2.5669775
H	-2.3606836	2.3384019	-1.0890051
H	-0.3146421	-2.8544003	-2.9638233
H	3.3927149	1.5757103	-1.6196136
H	1.7630545	-3.7303924	-0.1730509
H	0.3395404	2.8500570	-2.8835263
H	-0.0941835	-1.2361384	3.2150854
H	-2.5287468	2.2296654	0.6739705
H	-3.1727786	-1.6997826	2.0195491
H	0.3422614	1.6643101	3.3541831
H	-3.2700396	-0.0886831	1.2999021
H	-1.2510081	2.3247528	2.6657848
H	-3.3991552	-1.5844874	-1.6978313
H	2.1524041	0.3537162	2.7199414
H	-0.1200249	-2.9692361	2.8686759
H	-1.7925441	3.6584853	-0.0606838
H	-2.1619783	-0.3928565	2.6516986

Df$^-$ Ph$_2$CNNHB(C$_6$F$_5$)$_3$$^-$ anion (**1$^-$** in text)
60
Energy = -2821.541490934

B	-0.7405543	0.1876153	0.2928834
C	-0.3391348	-0.4620495	1.7641811
C	-1.3511854	1.7174608	0.4695166
C	-1.7581060	-0.8271298	-0.5552178
C	-1.0979624	-1.4030164	2.4594115
C	0.8492665	-0.1070289	2.4071432
C	-1.3631969	2.6270475	-0.5949770
C	-1.8609648	2.2433422	1.6577566
C	-1.4207524	-2.1704488	-0.7688621
C	-2.9527251	-0.4505189	-1.1708822
C	-0.7023453	-1.9993534	3.6525920
F	-2.3229798	-1.7807065	2.0014245
C	1.2913921	-0.6790917	3.5973111
F	1.6436030	0.8753656	1.9079825
C	-1.7929739	3.9455444	-0.4990555
F	-0.9867363	2.2314684	-1.8413194
C	-2.3077575	3.5568023	1.8024301
F	-1.9768041	1.4784995	2.7740752
C	-2.1611620	-3.0478193	-1.5554799
F	-0.3473116	-2.7215928	-0.1550845
C	-3.7298073	-1.2949873	-1.9635664
F	-3.4677180	0.8008426	-1.0154031
C	0.5114937	-1.6413178	4.2258862
F	-1.4880687	-2.9134899	4.2683639
F	2.4600050	-0.2960248	4.1611993
C	-2.2682126	4.4212234	0.7175054
F	-1.7780774	4.7609623	-1.5765213
F	-2.7844145	3.9963405	2.9880990
C	-3.3278533	-2.6068073	-2.1681862
F	-1.7726546	-4.3345065	-1.7110302
F	-4.8754715	-0.8499634	-2.5312280
F	0.9162001	-2.2048817	5.3836345
F	-2.6964949	5.6932609	0.8363181
F	-4.0619411	-3.4414974	-2.9333825
N	0.5504404	0.3812672	-0.5382255
N	1.4485618	-0.5797181	-0.6743673
C	2.6359472	-0.3478215	-1.1834857
C	3.0629774	0.9997073	-1.6523843
C	3.5600360	-1.4864111	-1.2489209
C	2.3408068	1.6783710	-2.6481225
C	4.1758004	1.6417359	-1.0849505
C	3.2636354	-2.6964023	-0.5833140
C	4.7593139	-1.4197056	-1.9853798

C	2.7180180	2.9557006	-3.0637680
H	1.4781720	1.1960199	-3.0993428
C	4.5593761	2.9148177	-1.5038033
H	4.7386010	1.1314072	-0.3078982
C	4.1253656	-3.7830653	-0.6601731
H	2.3425399	-2.7550387	-0.0132562
C	5.6236678	-2.5130211	-2.0560470
H	5.0092734	-0.5048583	-2.5152407
C	3.8301670	3.5781358	-2.4943934
H	2.1436823	3.4632202	-3.8342614
H	5.4238361	3.3944647	-1.0520280
C	5.3153590	-3.7035492	-1.3961806
H	3.8745293	-4.7026549	-0.1367906
H	6.5398424	-2.4338133	-2.6366915
H	4.1251183	4.5731631	-2.8164152
H	5.9881950	-4.5554614	-1.4504841
H	0.8623016	1.3249170	-0.7746774

Dh$^-$ $Ph_2CNNHBPh_3^-$ (**4$^-$** in text)
60
Energy = -1332.263755700

B	-0.6322974	0.1836501	0.3574749
C	-0.4098946	-0.5193636	1.8142638
C	-1.2740360	1.6800128	0.5021312
C	-1.6025865	-0.7523616	-0.5760285
C	-1.3833521	-1.3363664	2.4139938
C	0.7570856	-0.2884531	2.5645198
C	-1.3966729	2.5031922	-0.6344999
C	-1.7338269	2.2183276	1.7143097
C	-1.0955701	-1.8292709	-1.3262184
C	-2.9955347	-0.5614163	-0.6193129
C	-1.2128749	-1.8886111	3.6866343
H	-2.2977524	-1.5525203	1.8639574
C	0.9463685	-0.8346346	3.8354040
H	1.5410186	0.3295167	2.1319676
C	-1.9286762	3.7915977	-0.5692842
H	-1.0747345	2.1171689	-1.6018692
C	-2.2821246	3.5022555	1.7961602
H	-1.6587489	1.6138085	2.6162181
C	-1.9255115	-2.6653290	-2.0764113
H	-0.0234434	-2.0140049	-1.3096194
C	-3.8411082	-1.3949761	-1.3578747
H	-3.4282518	0.2673224	-0.0607121
C	-0.0422245	-1.6408596	4.4066577
H	-1.9910943	-2.5181359	4.1158889
H	1.8668473	-0.6360059	4.3820746
C	-2.3778672	4.2992095	0.6534619
H	-2.0006804	4.3991083	-1.4698629
H	-2.6316869	3.8841369	2.7540738
C	-3.3078281	-2.4543894	-2.0953453
H	-1.4958459	-3.4870601	-2.6477254
H	-4.9152362	-1.2151734	-1.3632707
H	0.1004796	-2.0727756	5.3950303
H	-2.7985836	5.3005933	0.7129302
H	-3.9578259	-3.1035269	-2.6783248
N	0.7492419	0.4210664	-0.3393575
N	1.6382881	-0.5275698	-0.5271143
C	2.7860661	-0.3216788	-1.1485654
C	3.1678194	0.9917612	-1.7265672
C	3.7015821	-1.4625347	-1.2156279
C	2.3204191	1.6711694	-2.6212866
C	4.3791816	1.6113087	-1.3720513
C	3.4903616	-2.6091831	-0.4148065
C	4.8122970	-1.4758838	-2.0858278
C	2.6684583	2.9200707	-3.1357944
H	1.3864257	1.2053968	-2.9255283
C	4.7342163	2.8545555	-1.8928876

H	5.0467262	1.1024387	-0.6816749
C	4.3418561	-3.7046255	-0.4914369
H	2.6428400	-2.6086982	0.2634487
C	5.6678194	-2.5751082	-2.1533192
H	4.9978845	-0.6166439	-2.7245545
C	3.8774566	3.5188717	-2.7746685
H	1.9962738	3.4237553	-3.8258403
H	5.6792805	3.3093721	-1.6057131
C	5.4436229	-3.7007023	-1.3578807
H	4.1549721	-4.5709747	0.1398427
H	6.5127035	-2.5521301	-2.8382066
H	4.1492870	4.4918115	-3.1756216
H	6.1131672	-4.5554046	-1.4069702
H	0.9883784	1.3559501	-0.6835274

Dh$^\cdot$ $Ph_2CNNHBPh_3^\cdot$
60
Energy = -1332.120768434

B	-0.6134242	0.1834973	0.4042523
C	-0.3824571	-0.5803774	1.8151465
C	-1.1747396	1.7017920	0.5468609
C	-1.5783915	-0.7093914	-0.5979815
C	-1.3045891	-1.5092323	2.3203721
C	0.7262849	-0.2774285	2.6265690
C	-1.2869463	2.5300559	-0.5854125
C	-1.5799273	2.2470518	1.7743392
C	-1.1046455	-1.8649412	-1.2362505
C	-2.9347818	-0.3873394	-0.7568078
C	-1.1401412	-2.1017125	3.5753253
H	-2.1693814	-1.7759531	1.7168334
C	0.9094543	-0.8696059	3.8760435
H	1.4628503	0.4438610	2.2768766
C	-1.7646381	3.8386661	-0.5020599
H	-1.0190679	2.1359001	-1.5662621
C	-2.0734756	3.5514206	1.8716672
H	-1.5093079	1.6352706	2.6705469
C	-1.9498823	-2.6786510	-1.9930514
H	-0.0587599	-2.1434913	-1.1276385
C	-3.7917399	-1.2025410	-1.5023998
H	-3.3291395	0.5078305	-0.2829224
C	-0.0272721	-1.7880002	4.3576336
H	-1.8774555	-2.8135945	3.9397698
H	1.7789166	-0.6124720	4.4771764
C	-2.1635195	4.3545819	0.7333551
H	-1.8371717	4.4511652	-1.3977408
H	-2.3853715	3.9429087	2.8375025
C	-3.3009242	-2.3497537	-2.1278718
H	-1.5565433	-3.5704366	-2.4759016
H	-4.8429134	-0.9387093	-1.5969600
H	0.1097231	-2.2524755	5.3308637
H	-2.5442442	5.3701305	0.8064853
H	-3.9631603	-2.9802193	-2.7158072
N	0.7541545	0.3367732	-0.3307296
N	1.6445296	-0.6029460	-0.4306992
C	2.7797404	-0.3726455	-1.1159637
C	3.0607343	0.9247206	-1.7698363
C	3.7254778	-1.4692075	-1.1537162
C	2.1979080	1.4354075	-2.7562211
C	4.1719125	1.6859395	-1.3702717
C	3.5291658	-2.6142300	-0.3425447
C	4.8393879	-1.4328314	-2.0229389
C	2.4443122	2.6824889	-3.3276335
H	1.3533174	0.8402615	-3.0936937
C	4.4067124	2.9365971	-1.9366584
H	4.8383245	1.2984483	-0.6051300
C	4.4175685	-3.6762083	-0.4056939
H	2.6762312	-2.6361773	0.3268368

C	5.7226830	-2.5037320	-2.0799749
H	4.9893164	-0.5707512	-2.6643187
C	3.5435141	3.4373230	-2.9140508
H	1.7789348	3.0620761	-4.0969684
H	5.2629755	3.5214718	-1.6146628
C	5.5170865	-3.6277669	-1.2735545
H	4.2618718	-4.5493164	0.2211080
H	6.5695618	-2.4706902	-2.7586407
H	3.7290543	4.4121762	-3.3546710
H	6.2083710	-4.4637513	-1.3213508
H	0.9686095	1.2288893	-0.8032418

Ef Cp*CoC$_5$Me$_4$CH$_2$B(C$_6$F$_5$)$_3$ (**2** in text)

84

Energy = -4372.619077765

Co	0.0050225	0.2265172	-0.0455656
C	-1.9454209	2.4022141	1.2582990
C	1.2520849	2.9577087	-1.1515199
C	-1.7367056	1.0707713	0.6093927
C	1.4238167	1.5711296	-0.6205504
C	-2.1002869	1.7935094	-1.8771051
C	1.6439567	2.1525811	1.9195963
C	-1.8044014	0.7974269	-0.8017049
C	1.5984668	1.2114190	0.7594274
C	-1.4902160	-0.1719569	1.2912709
C	1.5145830	0.3700461	-1.4060606
C	-1.5995372	-0.6152114	-0.9911261
C	1.8009684	-0.2113827	0.8221638
C	-1.4105179	-0.3549306	2.7731827
C	1.4297334	0.3089940	-2.8956322
C	-1.4053027	-1.2141697	0.3018313
C	1.7716376	-0.7367909	-0.5207360
C	-1.6371062	-1.3492522	-2.2932027
C	2.0386803	-0.9901786	2.0751496
C	-1.2048769	-2.6717620	0.5653202
C	1.9807152	-2.1556429	-0.9028008
H	-1.6009498	3.2197497	0.6195572
H	0.7106327	3.5969531	-0.4491599
H	-1.6040385	1.5330647	-2.8155611
H	1.2425651	1.6947167	2.8275947
H	-1.7896409	2.8003250	-1.5884486
H	1.0889278	3.0714196	1.7171142
H	-1.4233233	2.4675037	2.2158169
H	0.7209399	2.9603985	-2.1060572
H	-0.9425990	0.5038807	3.2622274
H	0.7350398	1.0550744	-3.2909957
H	-0.8487161	-1.2533530	3.0376469
H	1.1132272	-0.6781468	-3.2348928
H	-1.4234636	-0.6866639	-3.1347411
H	1.4765791	-0.5774458	2.9163687
H	-3.0141120	2.5611388	1.4512798
H	2.2372475	3.4114312	-1.3198598
H	-3.1799853	1.8219003	-2.0718165
H	2.6855600	2.4318277	2.1251707
H	-0.9193263	-2.1739932	-2.3098608
H	1.7662146	-2.0407846	1.9518528
H	-0.6589580	-2.8403994	1.4967834
H	1.2984971	-2.4196585	-1.7113187
H	-0.6559788	-3.1539797	-0.2465815
H	1.7140673	-2.7814719	-0.0456997
H	-2.4206120	-0.4604926	3.1891221
H	2.4170801	0.5112302	-3.3274231
H	-2.6352417	-1.7772112	-2.4504153
H	3.1012782	-0.9532925	2.3409205
H	-2.1783622	-3.1707313	0.6543035
C	4.4883422	-1.9647992	-0.1213993
C	4.9069054	-2.6725740	1.0075010
C	4.7904746	-0.5995602	-0.0645593
C	5.5992810	-2.1059709	2.0773872
C	5.4646131	0.0157843	0.9843869
C	5.8822002	-0.7459678	2.0701560
C	3.9797786	-2.1098390	-2.8516577
C	3.1358220	-2.3835693	-3.9345796
C	5.1999552	-1.5432003	-3.2384046
C	3.4113995	-2.0638009	-5.2596676
C	5.5234697	-1.2039542	-4.5516614
C	4.6198747	-1.4554047	-5.5756133
C	3.6805690	-4.2478947	-1.4390709
C	4.9443497	-4.8150372	-1.6377782
C	2.6436172	-5.1816931	-1.4649024
C	5.1840507	-6.1695397	-1.8317922
C	2.8308296	-6.5506073	-1.6596024
C	4.1106707	-7.0539867	-1.8446930
B	3.5472118	-2.5962159	-1.3220279
F	6.0427745	-4.0160518	-1.6335260
F	6.4395360	-6.6370073	-2.0035101
F	4.3100848	-8.3725374	-2.0345079
F	1.7738051	-7.3930676	-1.6733978
F	1.3432609	-4.8108115	-1.2980059
F	1.9466269	-3.0209143	-3.7362504
F	2.5223654	-2.3418674	-6.2382457
F	4.9155386	-1.1339664	-6.8492739
F	6.7175324	-0.6432147	-4.8415630
F	6.1841649	-1.3009989	-2.3382869
F	4.4181079	0.2279247	-1.0757818
F	5.7102433	1.3445227	0.9651092
F	6.5352189	-0.1731957	3.0994387
F	5.9779628	-2.8608931	3.1312059
F	4.6290472	-3.9961683	1.1456654

Fc Cp$_2$Fe with Cp = C$_5$H$_5$ (singlet)

21

Energy = -1651.175682763

Fe	-0.0001158	-0.0009877	-0.0000167
C	-0.0004729	1.2195061	1.6288779
C	0.0012303	1.2194391	-1.6289953
C	0.7160966	-0.9863575	1.6312076
C	-0.7167423	-0.9859705	-1.6313307
C	0.7166383	-0.9868063	-1.6306581
C	-0.7172799	-0.9862841	1.6308529
C	1.1603767	0.3761777	-1.6292575
C	-1.1601647	0.3769824	1.6294340
C	1.1591248	0.3768564	1.6300183
C	-1.1589056	0.3775314	-1.6302569
H	-0.0004184	2.3011206	1.6008622
H	0.0018732	2.3010641	-1.6009335
H	1.3518875	-1.8614922	1.6063216
H	-1.3530782	-1.8606984	-1.6065264
H	1.3519375	-1.8622784	-1.6051446
H	-1.3531305	-1.8613592	1.6056688
H	2.1893048	0.7097597	-1.6020806
H	-2.1888645	0.7112222	1.6024255
H	2.1878936	0.7109857	1.6034713
H	-2.1874673	0.7123316	-1.6040783

Fc$^{\cdot+}$ Cp$_2$Fe$^{\cdot+}$ (doublet)

21

Energy = -1650.986708241

Fe	-0.0000011	0.0000009	-0.1099526
C	-0.0000099	-1.7636857	-1.3153289
C	0.0000101	1.7636863	-1.3153302
C	0.7180561	-1.6498600	0.8783424
C	-0.7180560	1.6498739	0.8783412
C	0.7180792	1.6498710	0.8783385

C	-0.7180797	-1.6498800	0.8783407
C	1.1535123	1.7182867	-0.4835042
C	-1.1535088	-1.7183096	-0.4835006
C	1.1534765	-1.7183119	-0.4834914
C	-1.1534736	1.7183369	-0.4834906
H	0.0000403	-1.7663672	-2.3972284
H	-0.0000431	1.7663672	-2.3972298
H	1.3568245	-1.5994027	1.7491487
H	-1.3568243	1.5994087	1.7491496
H	1.3568901	1.5993454	1.7491215
H	-1.3568978	-1.5993610	1.7491178
H	2.1796895	1.6988504	-0.8247268
H	-2.1796865	-1.6988884	-0.8247236
H	2.1796824	-1.6989257	-0.8246200
H	-2.1796801	1.6989647	-0.8246183

Fs Cp*$_2$Fe (singlet)

51

Energy = -2044.601100297

Fe	0.0000076	-0.0000026	0.0149362
C	-0.1721470	-1.7793641	-2.6514301
C	2.8473026	0.6324474	-1.3246263
C	-0.4209841	-1.6021585	-1.1846509
C	1.6246918	1.0864436	-0.5872386
C	1.8151108	-2.6295418	-0.2927769
C	0.1723868	1.7798678	-2.6510717
C	0.4730803	-1.9850611	-0.1237040
C	0.4210978	1.6023835	-1.1843042
C	-1.6246266	-1.0863167	-0.5876008
C	1.4744790	1.1502042	0.8427740
C	-0.1781533	-1.7059386	1.1290425
C	-0.4730747	1.9850680	-0.1233693
C	-2.8471565	-0.6321709	-1.3250303
C	2.5140751	0.7748678	1.8542878
C	-1.4745531	-1.1503528	0.8424145
C	0.1780476	1.7057184	1.1293834
C	0.3665506	-2.0096830	2.4913125
C	-1.8150995	2.6295641	-0.2924356
C	-2.5142517	-0.7751936	1.8538893
C	-0.3667780	2.0092157	2.4916585
H	0.8882059	-1.6607732	-2.8955455
H	2.5890163	0.1648535	-2.2798823
H	2.5003224	-2.3461554	0.5123392
H	-0.8879242	1.6611794	-2.8953225
H	2.2772630	-2.3460142	-1.2425051
H	0.7366865	1.0533027	-3.2424468
H	-0.7362858	-1.0525963	-3.2427091
H	3.4198905	-0.0927318	-0.7395637
H	-2.5887558	-0.1642348	-2.2800884
H	3.1890835	1.6187450	2.0557191
H	-3.4198968	0.0927652	-0.7398153
H	3.1289251	-0.0611023	1.5063552
H	1.4601613	-1.9992331	2.4953680
H	-2.2771009	2.3463105	-1.2423181
H	-0.4763061	-2.7822286	-2.9830407
H	3.5123855	1.4801952	-1.5421229
H	1.7289174	-3.7253031	-0.2814641
H	0.4764398	2.7828439	-2.9824400
H	0.0208704	-1.2826397	3.2328547
H	-2.5004273	2.3459059	0.5124851
H	-3.1894538	-1.6190185	2.0548916
H	-0.0211636	1.2820145	3.2330785
H	-3.1288897	0.0610206	1.5061658
H	-1.4603879	1.9987913	2.4956301
H	-3.5121368	-1.4798989	-1.5429203
H	2.0588994	0.4840083	2.8053272
H	0.0443474	-3.0044388	2.8302768
H	-1.7289321	3.7253236	-0.2807519
H	-2.0591792	-0.4847776	2.8051134
H	-0.0445795	3.0038893	2.8308621

Fs$^{•+}$ Cp*$_2$Fe$^{•+}$ (doublet)

51

Energy = -2044.435375424

Fe	0.0000201	-0.0000087	0.0102361
C	0.5722914	1.7906370	-2.6461279
C	-2.8924774	0.0193179	-1.4029148
C	0.8092817	1.5461261	-1.1894780
C	-1.8396242	-0.7140891	-0.6317453
C	-1.0861868	3.0654568	-0.2426909
C	-0.5723700	-1.7907058	-2.6461768
C	0.0668857	2.1226472	-0.1098266
C	-0.8093519	-1.5461993	-1.1895281
C	1.8395740	0.7140022	-0.6316894
C	-1.7310500	-0.7784870	0.8059241
C	0.6284931	1.6518396	1.1232204
C	-0.0668426	-2.1225948	-0.1098960
C	2.8924493	-0.0193828	-1.4028533
C	-2.6649697	-0.1443116	1.7891897
C	1.7310580	0.7785203	0.8059884
C	-0.6284077	-1.6517283	1.1231761
C	0.1942146	2.0521498	2.4979302
C	1.0862325	-3.0654018	-0.2427694
C	2.6649680	0.1443576	1.7892694
C	-0.1941561	-2.0521211	2.4978794
H	-0.4860465	1.9605089	-2.8606744
H	-2.5179962	0.3633318	-2.3706212
H	-1.7991009	2.9498391	0.5778059
H	0.4859779	-1.9604913	-2.8607430
H	-1.6146226	2.9249616	-1.1883479
H	-0.9228084	-0.9550082	-3.2560740
H	0.9226395	0.9548980	-3.2560203
H	-3.2663612	0.8833456	-0.8488031
H	2.5179846	-0.3633873	-2.3705691
H	-3.5184280	-0.8081695	1.9751104
H	3.2663299	-0.8834183	-0.8487505
H	-3.0604171	0.8036696	1.4154023
H	-0.8638651	2.3230822	2.5231151
H	1.6146184	-2.9249484	-1.1884611
H	1.1204734	2.6861055	-2.9650340
H	-3.7441776	-0.6449258	-1.5956103
H	-0.7214423	4.1001455	-0.2176809
H	-1.1204803	-2.6862282	-2.9650556
H	0.3665836	1.2533058	3.2237404
H	1.7991949	-2.9497295	0.5776773
H	3.5184712	0.8081797	1.9751135
H	-0.3668881	-1.2534423	3.2237850
H	3.0603512	-0.8036723	1.4155377
H	0.8640079	-2.3227073	2.5231690
H	3.7441488	0.6448686	-1.5955254
H	-2.1743084	0.0394924	2.7478282
H	0.7682973	2.9270082	2.8282393
H	0.7215105	-4.1000978	-0.2176888
H	2.1743239	-0.0393401	2.7479374
H	-0.7680023	-2.9271997	2.8279998

Rco$^{•}$ Cp*$_2$Co$^{•}$ (doublet)

51

Energy = -2163.629450756

Co	0.0000019	-0.0000032	0.0133037
C	-0.1272345	-1.9013442	-2.6411480
C	2.8215841	0.6852846	-1.3892988
C	-0.4301256	-1.7046733	-1.1867471
C	1.6145902	1.1457929	-0.6295598

C	1.8044191	-2.6409769	-0.2042511
C	0.1278118	1.9023230	-2.6407262
C	0.4497362	-2.0102529	-0.0862327
C	0.4300625	1.7054353	-1.1862219
C	-1.6149676	-1.1463270	-0.6304130
C	1.5119959	1.2018874	0.8235079
C	-0.2555183	-1.7726609	1.1548068
C	-0.4496961	2.0104257	-0.0861119
C	-2.8220784	-0.6858064	-1.3899430
C	2.5907615	0.7970815	1.7818637
C	-1.5115894	-1.2013716	0.8227125
C	0.2554580	1.7717253	1.1552832
C	0.2656017	-2.0608477	2.5299921
C	-1.8043838	2.6412241	-0.2036334
C	-2.5903198	-0.7966205	1.7811408
C	-0.2661751	2.0596871	2.5303206
H	0.9359197	-1.7463616	-2.8521927
H	2.5517141	0.3160841	-2.3834393
H	2.4637337	-2.3317284	0.6126850
H	-0.9356594	1.7496225	-2.8518522
H	2.2870768	-2.3756915	-1.1490348
H	0.6950115	1.2027633	-3.2622321
H	-0.6959472	-1.2032840	-3.2629464
H	3.3435284	-0.1174006	-0.8605131
H	-2.5524404	-0.3174449	-2.3844614
H	3.3242291	1.6046028	1.9173254
H	-3.3433268	0.1176026	-0.8615572
H	3.1375409	-0.0801205	1.4216220
H	1.3548080	-1.9642475	2.5707011
H	-2.2870081	2.3766997	-1.1486540
H	-0.3804162	-2.9189817	-2.9721295
H	3.5381221	1.5076691	-1.5248963
H	1.7288489	-3.7367540	-0.1679131
H	0.3833607	2.9192449	-2.9720976
H	-0.1589558	-1.3744855	3.2695685
H	-2.4637067	2.3312510	0.6130179
H	-3.3232295	-1.6045509	1.9171172
H	0.1594830	1.3743557	3.2702043
H	-3.1376933	0.0800673	1.4205742
H	-1.3552243	1.9614550	2.5710634
H	-3.5392173	-1.5078247	-1.5246183
H	2.1786250	0.5564933	2.7662493
H	0.0163549	-3.0834804	2.8479937
H	-1.7288786	3.7369750	-0.1663233
H	-2.1780881	-0.5553766	2.7653218
H	-0.0184993	3.0828640	2.8477632

Rco$^+$ Cp*$_2$Co$^+$ (singlet)
51
Energy = -2163.528475998

Co	0.0000034	0.0000041	-0.0002448
C	0.5480265	1.7181956	-2.6495343
C	-2.8847516	0.0641667	-1.3962185
C	0.7774962	1.4688595	-1.1939625
C	-1.8298655	-0.6656707	-0.6293177
C	-1.1215135	2.9918399	-0.2419481
C	-0.5480344	-1.7182001	-2.6495118
C	0.0254538	2.0429641	-0.1093815
C	-0.7774997	-1.4688613	-1.1939387
C	1.8298671	0.6656737	-0.6293421
C	-1.7281742	-0.7433041	0.8042129
C	0.6131498	1.5946307	1.1254804
C	-0.0254455	-2.0429515	-0.1093576
C	2.8847546	-0.0641628	-1.3962409
C	-2.6590269	-0.1081788	1.7857634
C	1.7281805	0.7433163	0.8041881
C	-0.6131394	-1.5946113	1.1255061
C	0.1840709	1.9972254	2.4992591
C	1.1215275	-2.9918224	-0.2419288
C	2.6590336	0.1082000	1.7857437
C	-0.1840845	-1.9972755	2.4992761
H	-0.5108167	1.8725268	-2.8722116
H	-2.5108644	0.4233057	-2.3583630
H	-1.8344916	2.8831639	0.5793108
H	0.5108153	-1.8724668	-2.8722035
H	-1.6530556	2.8524097	-1.1858098
H	-0.9168032	-0.8933078	-3.2630209
H	0.9167379	0.8932779	-3.2630435
H	-3.2760302	0.9152176	-0.8345408
H	2.5108902	-0.4232415	-2.3584166
H	-3.5123044	-0.7737640	1.9673644
H	3.2759883	-0.9152527	-0.8345910
H	-3.0558720	0.8394840	1.4129847
H	-0.8757226	2.2595311	2.5302406
H	1.6530483	-2.8524008	-1.1858021
H	1.0844315	2.6248557	-2.9567831
H	-3.7247579	-0.6115961	-1.6008293
H	-0.7498106	4.0240954	-0.2205677
H	-1.0843900	-2.6248936	-2.9567474
H	0.3692531	1.2057395	3.2299315
H	1.8345263	-2.8831285	0.5793103
H	3.5123197	0.7737794	1.9673257
H	-0.3695714	-1.2059297	3.2300256
H	3.0558684	-0.8394753	1.4129839
H	0.8757746	-2.2592991	2.5303542
H	3.7247886	0.6115849	-1.6007883
H	-2.1712150	0.0718957	2.7461584
H	0.7529054	2.8795317	2.8188301
H	0.7498468	-4.0240844	-0.2205116
H	2.1712231	-0.0718430	2.7461454
H	-0.7527406	-2.8797529	2.8186810

Rcr Cp*$_2$Cr (triplet)
51
Energy = -1825.308773808

Cr	0.0109040	-0.0002171	0.0361800
C	-1.8143082	1.9236902	-1.8966629
C	1.8216219	0.3795208	-2.6465642
C	-1.7641000	0.8584780	-0.8405794
C	1.7587533	0.1777586	-1.1604029
C	-1.8500028	-1.2266840	-2.4051837
C	1.8602549	2.6849041	-0.4198786
C	-1.8120895	-0.5551834	-1.0642410
C	1.8171239	1.2082256	-0.1572943
C	-1.7126605	1.0859635	0.5852324
C	1.7342255	-1.1010541	-0.4784837
C	-1.7867137	-1.2152610	0.2031218
C	1.8280411	0.5836466	1.1274611
C	-1.7811528	2.4196318	1.2713765
C	1.8091242	-2.4516395	-1.1297433
C	-1.7257126	-0.2106562	1.2340812
C	1.7737457	-0.8363125	0.9421891
C	-1.8269736	-2.6977948	0.4312992
C	1.8656732	1.2937001	2.4484722
C	-1.7894584	-0.4528945	2.7142101
C	1.8131318	-1.8737180	2.0258659
H	-1.3907299	1.5693512	-2.8413495
H	1.3907712	1.3429641	-2.9363087
H	-1.3825261	-2.2153639	-2.3704473
H	1.3808932	3.2492449	0.3859616
H	-1.3292018	-0.6331388	-3.1629168
H	1.3512061	2.9370137	-1.3552430
H	-1.2553900	2.8139170	-1.5916122
H	1.2723728	-0.4047493	-3.1769725

H	-1.3342804	3.2064111	0.6553319
H	1.3165754	-2.4503265	-2.1072682
H	-1.2504869	2.4040917	2.2285781
H	1.3265018	-3.2178863	-0.5149040
H	-1.4172589	-3.2454429	-0.4225724
H	1.4043505	2.2836840	2.3832821
H	-2.8486088	2.2384960	-2.0952707
H	2.8585387	0.3577536	-3.0120011
H	-2.8848417	-1.3626224	-2.7480390
H	2.8960834	3.0422070	-0.5031263
H	-1.2514207	-2.9795147	1.3187608
H	1.3397302	0.7261992	3.2225431
H	-1.2594255	0.3289166	3.2674226
H	1.2696428	-2.7768967	1.7311506
H	-1.3396683	-1.4142625	2.9811283
H	1.3651668	-1.5011553	2.9521557
H	-2.8208074	2.7138379	1.4770165
H	2.8516876	-2.7647452	-1.2877826
H	-2.8574582	-3.0472429	0.5850548
H	2.9005974	1.4339007	2.7896673
H	-2.8282705	-0.4628783	3.0753751
H	2.8459311	-2.1730451	2.2546573

Rcr^{+} Cp*$_2$Cr^{+} (quartet)
51
Energy = -1825.180947584

Cr	-0.0002648	0.0533939	-0.0213084
C	-1.8309508	2.2069230	1.6815834
C	1.8695295	2.7336049	-0.4731502
C	-1.8168414	1.0255473	0.7587775
C	1.8341704	1.2530683	-0.2397074
C	-1.8811928	2.3151631	-1.5155379
C	1.9080239	1.2803800	2.3740572
C	-1.8399126	1.0741050	-0.6755319
C	1.8511520	0.6011544	1.0388695
C	-1.8168697	-0.3538303	1.1560790
C	1.8153762	0.2383046	-1.2544850
C	-1.8540774	-0.2753046	-1.1645404
C	1.8432463	-0.8165812	0.8143495
C	-1.8302579	-0.8619117	2.5665085
C	1.8286975	0.4711748	-2.7354552
C	-1.8397328	-1.1578041	-0.0326133
C	1.8210348	-1.0407846	-0.6031132
C	-1.9139947	-0.6907139	-2.6038197
C	1.8893836	-1.8753940	1.8747318
C	-1.8811135	-2.6556361	-0.0844199
C	1.8401647	-2.3748682	-1.2867288
H	-1.3530028	3.0791340	1.2275618
H	1.3787881	3.2814423	0.3358300
H	-1.3951136	2.1636568	-2.4832250
H	1.4167341	0.6869291	3.1500293
H	-1.3978583	3.1574409	-1.0139731
H	1.4393298	2.2673023	2.3443732
H	-1.3251365	1.9872381	2.6253858
H	1.3878074	3.0044653	-1.4161218
H	-1.2997685	-0.1868379	3.2437172
H	1.3466819	1.4162761	-2.9996856
H	-1.3773100	-1.8537161	2.6419786
H	1.3265944	-0.3359857	-3.2749266
H	-1.4461958	0.0503199	-3.2571296
H	1.4080376	-1.5425465	2.7980253
H	-2.8652450	2.4848742	1.9192307
H	2.9090116	3.0808748	-0.5220749
H	-2.9219634	2.6011472	-1.7116108
H	2.9523510	1.4203049	2.6794973
H	-1.4234129	-1.6543088	-2.7663224
H	1.4041859	-2.7979622	1.5448859
H	-1.3945316	-3.1027578	0.7864750
H	1.3091776	-2.3463715	-2.2421873
H	-1.3983724	-3.0415025	-0.9860820
H	1.3908793	-3.1529881	-0.6644717
H	-2.8634295	-0.9425736	2.9263640
H	2.8629341	0.5165350	-3.0985700
H	-2.9589656	-0.7964167	-2.9205558
H	2.9313208	-2.1194388	2.1161350
H	-2.9219695	-3.0020656	-0.0943900
H	2.8745925	-2.6750337	-1.4948530

Rf$^{\cdot-}$ B(C$_6$F$_5$)$_3$$^{\cdot-}$
34
Energy = -2209.632469086

B	0.0012033	-0.0109023	-0.0014887
C	0.0008166	1.5556515	-0.0005609
C	1.3574151	-0.7956919	0.0057122
C	-1.3549682	-0.7958387	-0.0081460
C	-0.9727283	2.3283194	0.6612238
C	0.9736905	2.3297199	-0.6617090
C	2.5021306	-0.3560458	0.6968789
C	1.5486165	-2.0111401	-0.6780352
C	-1.5461370	-2.0111004	0.6759469
C	-2.5005237	-0.3550835	-0.6972464
C	-0.9933812	3.7157595	0.6710392
C	0.9920768	3.7171961	-0.6717822
C	3.7111279	-1.0369712	0.7153314
C	2.7396722	-2.7233016	-0.6845275
C	-2.7378989	-2.7220838	0.6845456
C	-3.7102477	-1.0347595	-0.7134813
C	-0.0012742	4.4260241	-0.0005116
C	3.8391085	-2.2356093	0.0177173
C	-3.8379891	-2.2335268	-0.0160654
F	-1.9483274	1.7140932	1.3823473
F	1.9511388	1.7169096	-1.3815073
F	2.4487964	0.7829520	1.4377346
F	0.5390722	-2.5325226	-1.4251964
F	-0.5361795	-2.5330099	1.4221655
F	-2.4476543	0.7843792	-1.4374258
F	-1.9531120	4.3915308	1.3464623
F	1.9506581	4.3944137	-1.3473924
F	4.7640920	-0.5649930	1.4241788
F	2.8563253	-3.8761755	-1.3854885
F	-2.8547457	-3.8742396	1.3866236
F	-4.7644142	-0.5611151	-1.4194283
F	-0.0024448	5.7773108	-0.0007637
F	5.0085016	-2.9125388	0.0222082
F	-5.0078157	-2.9097055	-0.0190789

Rf B(C$_6$F$_5$)$_3$
34
Energy = -2209.515903711

B	-0.0048640	0.0003719	0.0001165
C	0.7789229	1.3532379	-0.0020124
C	0.7789482	-1.3525317	0.0021270
C	-1.5672766	0.0002564	0.0001365
C	0.3578124	2.4682525	0.7383232
C	1.9538299	1.5349954	-0.7472212
C	1.9538023	-1.5345280	0.7473674
C	0.3576413	-2.4675363	-0.7382159
C	-2.3182270	-0.9428809	0.7192528
C	-2.3182682	0.9432280	-0.7191447
C	1.0480835	3.6729219	0.7560863
C	2.6565140	2.7326004	-0.7761789
C	2.6562282	-2.7322802	0.7763149
C	1.0475713	-3.6724315	-0.7558612
C	-3.7066190	-0.9516225	0.7407790

C	-3.7066565	0.9517836	-0.7408482
C	2.2019773	3.8070888	-0.0137989
C	2.2015057	-3.8067939	0.0140194
C	-4.4051322	0.0000490	-0.0000635
F	-0.7473587	2.3900302	1.5089166
F	2.4300703	0.5310749	-1.5131689
F	2.4303773	-0.5306497	1.5131408
F	-0.7475477	-2.3891138	-1.5088482
F	-1.6946191	-1.8783563	1.4653526
F	-1.6946813	1.8786397	-1.4653387
F	0.6181848	4.7045402	1.4992386
F	3.7614981	2.8678091	-1.5261969
F	3.7613171	-2.8676634	1.5261525
F	0.6173824	-4.7037750	-1.4992184
F	-4.3799580	-1.8611150	1.4624410
F	-4.3800051	1.8611354	-1.4626804
F	2.8710400	4.9636148	-0.0208090
F	2.8702687	-4.9635451	0.0213702
F	-5.7410307	-0.0000288	-0.0001288

Rh⁻ BPh_3^{-}

34

Energy = -720.3448410792

B	0.0020553	-0.0136715	0.0001346
C	0.0008924	1.5533619	0.0007692
C	1.3587474	-0.7971243	0.0085525
C	-1.3555386	-0.7967208	-0.0093016
C	-1.0654689	2.3183597	0.5499975
C	1.0655721	2.3211926	-0.5477352
C	2.5512726	-0.2588385	0.5675096
C	1.4937699	-2.1021104	-0.5420294
C	-1.4927855	-2.1001764	0.5439121
C	-2.5461512	-0.2587373	-0.5722507
C	-1.0712745	3.7106981	0.5550679
C	1.0676506	3.7135365	-0.5528962
C	3.7585833	-0.9522834	0.5814097
C	2.6972299	-2.8023063	-0.5383226
C	-2.6973130	-2.7987057	0.5407614
C	-3.7545571	-0.9503457	-0.5855218
C	-0.0028087	4.4295380	0.0010279
C	3.8489269	-2.2366163	0.0265979
C	-3.8475708	-2.2325676	-0.0263894
H	-1.9043305	1.7913277	0.9999819
H	1.9056662	1.7965473	-0.9982571
H	2.5129067	0.7308640	1.0180290
H	0.6223841	-2.5647252	-1.0009318
H	-0.6224166	-2.5630483	1.0045280
H	-2.5050744	0.7288298	-1.0271942
H	-1.9099677	4.2449114	0.9992816
H	1.9048114	4.2499776	-0.9973097
H	4.6379127	-0.4951068	1.0329571
H	2.7450341	-3.7948371	-0.9840769
H	-2.7470061	-3.7901023	0.9888083
H	-4.6324323	-0.4936418	-1.0403440
H	-0.0041754	5.5168581	0.0010701
H	4.7899153	-2.7813467	0.0346501
H	-4.7898588	-2.7750854	-0.0322060

Rh BPh_3

34

Energy = -720.2691895757

B	0.0009313	-0.0029900	0.0005893
C	0.7809350	1.3510984	0.0011166
C	0.7822076	-1.3571638	0.0002734
C	-1.5620046	-0.0020497	-0.0001684
C	0.2629725	2.4997015	0.6381202
C	2.0333183	1.4774582	-0.6378070
C	2.0346005	-1.4860835	0.6390288
C	0.2635375	-2.5046144	-0.6382764
C	-2.2984662	-1.0232005	0.6384579
C	-2.2972191	1.0205124	-0.6382776
C	0.9636050	3.7043821	0.6527937
C	2.7252188	2.6872604	-0.6549828
C	2.7243971	-2.6974196	0.6558333
C	0.9623976	-3.7104437	-0.6541242
C	-3.6920709	-1.0173867	0.6545382
C	-3.6909100	1.0163881	-0.6541327
C	2.1945151	3.8023874	-0.0012544
C	2.1924024	-3.8112069	0.0012354
C	-4.3918352	-0.0000616	-0.0000294
H	-0.6973305	2.4366708	1.1441010
H	2.4578012	0.6138536	-1.1436238
H	2.4616351	-0.6237776	1.1449825
H	-0.6962730	-2.4392902	-1.1448079
H	-1.7635683	-1.8227611	1.1447793
H	-1.7617323	1.8198977	-1.1442614
H	0.5513603	4.5687639	1.1666763
H	3.6791009	2.7627567	-1.1700627
H	3.6777360	-2.7745403	1.1719976
H	0.5491710	-4.5731754	-1.1700414
H	-4.2348756	-1.8055233	1.1695322
H	-4.2330176	1.8052943	-1.1687925
H	2.7376825	4.7437707	-0.0017541
H	2.7339178	-4.7534768	0.0012137
H	-5.4785200	0.0002141	-0.0002142

Rn⁻ $Ph_2CN_2^{-}$

25

Energy = -611.3220482677

N	0.2831716	0.5625642	-1.5703589
N	1.1968608	-0.2163212	-1.3689735
C	2.5687448	-0.0961961	-1.3138625
C	3.2038440	1.2059934	-1.4648894
C	3.2701680	-1.3465236	-1.0728015
C	2.5646680	2.2613755	-2.1650630
C	4.4521088	1.5156514	-0.8635728
C	2.5905798	-2.4753509	-0.5408290
C	4.6373860	-1.5399586	-1.3980112
C	3.1466804	3.5222157	-2.2759158
H	1.5987224	2.0756896	-2.6221183
C	5.0270714	2.7774346	-0.9755745
H	4.9604387	0.7559277	-0.2782816
C	3.2308646	-3.6934865	-0.3461797
H	1.5399660	-2.3683002	-0.2861919
C	5.2763396	-2.7605186	-1.1924410
H	5.1970785	-0.7257817	-1.8473210
C	4.3853274	3.7977217	-1.6891765
H	2.6248363	4.2987133	-2.8324060
H	5.9826554	2.9709831	-0.4918958
C	4.5870303	-3.8550376	-0.6605396
H	2.6686287	-4.5278345	0.0693568
H	6.3251871	-2.8606206	-1.4655934
H	4.8355958	4.7828213	-1.7771358
H	5.0881328	-4.8055040	-0.4988319

Rn Ph_2CN_2

25

Energy = -611.2387774962

N	0.1227301	-0.1152459	-1.5371991
N	1.2633835	-0.0865771	-1.4433748
C	2.5703931	-0.0523598	-1.3357008
C	3.2086795	1.2682973	-1.4630008
C	3.2488823	-1.3372477	-1.1009519
C	2.6177243	2.2942062	-2.2227277

C	4.4184934	1.5363131	-0.7967920
C	2.5919845	-2.4040875	-0.4609432
C	4.5702366	-1.5300021	-1.5439400
C	3.2202531	3.5462695	-2.3144456
H	1.6876044	2.1079375	-2.7531366
C	5.0213166	2.7874577	-0.9024372
H	4.8771932	0.7664706	-0.1848859
C	3.2386224	-3.6220679	-0.2676407
H	1.5735658	-2.2765823	-0.1034647
C	5.2137715	-2.7481060	-1.3390701
H	5.0856912	-0.7277224	-2.0616820
C	4.4283213	3.7999012	-1.6604043
H	2.7479846	4.3232923	-2.9088333
H	5.9547010	2.9741423	-0.3786090
C	4.5546616	-3.8013687	-0.7007904
H	2.7137790	-4.4313771	0.2320529
H	6.2331242	-2.8769012	-1.6923842
H	4.9005687	4.7745762	-1.7389576
H	5.0590853	-4.7504122	-0.5454296

TSABf$^{\cdot-}$ (cyclization of **Af**$^{\cdot-}$)
59
Energy = -2820.882414361

B	0.4181161	0.1803281	0.0710944
C	1.1768536	0.6660065	1.4535129
C	1.3450098	-0.8056416	-0.8832619
C	-0.0623954	1.5849269	-0.6513106
C	2.3443116	1.4258325	1.3537684
C	0.7133660	0.4939403	2.7586547
C	0.8742188	-1.1754743	-2.1483526
C	2.5066996	-1.4728675	-0.4948888
C	-1.0592558	2.3526666	-0.0407542
C	0.4666658	2.1541793	-1.8094550
C	3.0275466	1.9638101	2.4364908
F	2.8817580	1.6616825	0.1272124
C	1.3685830	1.0130049	3.8770869
F	-0.4217861	-0.1900589	3.0271195
C	1.5047640	-2.0920494	-2.9809311
F	-0.2743445	-0.6353831	-2.6240856
C	3.1733785	-2.4020632	-1.2937048
F	3.0603772	-1.2720416	0.7303668
C	-1.5425533	3.5514460	-0.5538067
F	-1.5982222	1.9588074	1.1392319
C	0.0180089	3.3551200	-2.3567693
F	1.4859931	1.5613171	-2.4827891
C	2.5325881	1.7525771	3.7192836
F	4.1612707	2.6805138	2.2634847
F	0.8728768	0.8117400	5.1193354
C	2.6729347	-2.7133480	-2.5502383
F	0.9995945	-2.3893769	-4.1982234
F	4.2964561	-3.0108492	-0.8519140
C	-1.0017615	4.0585588	-1.7297439
F	-2.5223479	4.2341757	0.0810785
F	0.5703858	3.8480680	-3.4889517
F	3.1727418	2.2617733	4.7918550
F	3.3009409	-3.6096481	-3.3363476
F	-1.4508843	5.2212775	-2.2448931
N	-0.8153706	-0.6942735	0.4037336
N	-1.9717242	-0.5699850	-0.1353038
C	-2.7965516	-1.6309856	-0.0035980
C	-2.2024371	-2.6974747	0.7631013
C	-4.1730538	-1.5076814	-0.4605819
C	-0.7521465	-2.5807895	0.7899029
C	-2.8538756	-3.4696872	1.7375614
C	-4.7308004	-0.2418137	-0.7414475
C	-4.9836225	-2.6445884	-0.6490798
C	-0.0465681	-3.1585071	1.8968636
C	-2.1312507	-4.0550016	2.7742289
H	-3.9382142	-3.5355734	1.7329432
C	-6.0448744	-0.1229981	-1.1789392
H	-4.1104181	0.6386706	-0.6060486
C	-6.3025342	-2.5201561	-1.0823093
H	-4.5643159	-3.6311297	-0.4750785
C	-0.7326895	-3.8595840	2.8656908
H	1.0353234	-3.0711650	1.9397792
H	-2.6494112	-4.6237478	3.5411593
C	-6.8442029	-1.2603112	-1.3481044
H	-6.4548361	0.8623222	-1.3866051
H	-6.9072242	-3.4126616	-1.2222850
H	-0.1869027	-4.3015912	3.6960574
H	-7.8717297	-1.1640091	-1.6877025
H	-0.2393713	-2.6433344	-0.1728142

TSABh$^{\cdot-}$ (cyclization of **Ah**$^{\cdot-}$)
59
Energy = -1331.604737242

B	1.3296694	0.4587188	0.0750052
C	2.1851745	0.1751825	1.4305066
C	1.9846200	-0.2683232	-1.2395172
C	1.1784203	2.0705338	-0.1229378
C	3.4665824	0.7375653	1.5851373
C	1.7129993	-0.6084829	2.4963607
C	1.3573690	-0.1247685	-2.4930614
C	3.1173070	-1.0955185	-1.1957215
C	0.3754765	2.8304907	0.7489299
C	1.8938962	2.7862903	-1.0962253
C	4.2404990	0.5240878	2.7263475
H	3.8633425	1.3650322	0.7879495
C	2.4749586	-0.8300546	3.6482977
H	0.7267374	-1.0594312	2.4185134
C	1.8318242	-0.7634032	-3.6386670
H	0.4690636	0.5015189	-2.5634188
C	3.6059036	-1.7436365	-2.3355434
H	3.6260322	-1.2399745	-0.2449761
C	0.2768934	4.2175674	0.6481067
H	-0.1907813	2.3173854	1.5229637
C	1.8168124	4.1795834	-1.2025374
H	2.5263479	2.2395687	-1.7928511
C	3.7462350	-0.2659274	3.7694897
H	5.2276454	0.9757584	2.8082328
H	2.0755825	-1.4451392	4.4530258
C	2.9651971	-1.5800881	-3.5644782
H	1.3197256	-0.6301205	-4.5898029
H	4.4856368	-2.3807424	-2.2632284
C	1.0015788	4.9038478	-0.3323325
H	-0.3624861	4.7694065	1.3350349
H	2.3901552	4.6985221	-1.9690133
H	4.3428085	-0.4348985	4.6632532
H	3.3395295	-2.0838902	-4.4527265
H	0.9307052	5.9862980	-0.4139500
N	-0.0880001	-0.2066829	0.2412382
N	-1.2106403	0.3023739	-0.0844932
C	-2.2440830	-0.5639153	-0.2284433
C	-1.8597237	-1.9205403	0.0736176
C	-3.5721759	-0.0268686	-0.4686772
C	-0.4151934	-2.0842953	-0.0186841
C	-2.6190261	-2.8559348	0.7938269
C	-3.8365569	1.3521490	-0.3033946
C	-4.6353991	-0.8528721	-0.8905844
C	0.1946889	-3.1302777	0.7518006
C	-1.9953538	-3.8898425	1.4916079
H	-3.6933634	-2.7208800	0.8845137
C	-5.1061815	1.8694320	-0.5323049
H	-3.0205356	2.0005384	0.0016983

C	-5.9079528	-0.3295832	-1.1128030
H	-4.4507004	-1.9085563	-1.0657089
C	-0.5838963	-3.9923306	1.4967301
H	1.2728630	-3.2532943	0.7069070
H	-2.5922560	-4.5893845	2.0706102
C	-6.1569360	1.0334199	-0.9325083
H	-5.2837265	2.9336775	-0.3958809
H	-6.7081692	-0.9897721	-1.4387527
H	-0.1092675	-4.7858155	2.0700919
H	-7.1490661	1.4405473	-1.1072200
H	0.0471365	-1.8938517	-0.9909247

TSADf$^{\bullet-}$ (H-abstraction from **Rco** with **Af**$^{\bullet-}$)
110
Energy = -4984.553414776

B	2.0977449	-0.4914364	-0.4067589
C	3.6437525	0.1384089	-0.3171255
C	2.2856053	-1.9905270	0.2951434
C	1.5123941	-0.5487261	-1.9525643
C	4.5917263	0.1376654	-1.3439545
C	4.1224978	0.6667321	0.8883683
C	1.9600403	-2.3397931	1.6094996
C	2.9319115	-3.0164625	-0.4037331
C	1.6140911	0.5567293	-2.8060272
C	0.6885457	-1.5590202	-2.4459358
C	5.8791574	0.6559378	-1.2214499
F	4.3072257	-0.3834297	-2.5667918
C	5.3991023	1.2000685	1.0538840
F	3.3558827	0.6602958	2.0019676
C	2.2060295	-3.5978476	2.1640369
F	1.3761536	-1.4671389	2.4633545
C	3.1993661	-4.2814207	0.1060083
F	3.3410227	-2.8121308	-1.6830289
C	0.9651580	0.6548536	-4.0330471
F	2.4282000	1.6039936	-2.5081727
C	0.0386053	-1.5158499	-3.6764572
F	0.4307807	-2.6798166	-1.7087109
C	6.2894507	1.1995560	-0.0119071
F	6.7392558	0.6348674	-2.2692317
F	5.7916491	1.7020386	2.2494341
C	2.8276829	-4.5820172	1.4106873
F	1.8481048	-3.8663453	3.4428062
F	3.8129825	-5.2227154	-0.6501178
C	0.1677309	-0.3913421	-4.4791526
F	1.1028844	1.7610128	-4.8059611
F	-0.7547331	-2.5402938	-4.0813051
F	7.5339956	1.7068572	0.1288345
F	3.0744593	-5.8023721	1.9347687
F	-0.4897664	-0.3060840	-5.6582648
N	1.1094251	0.3792534	0.4018211
N	0.7082843	1.4984501	-0.1784029
C	0.0636842	2.4218238	0.5479603
C	0.1947236	2.5608816	2.0147940
C	-0.5999702	3.4758261	-0.2287106
C	0.2521136	1.4802578	2.9154913
C	0.4611939	3.8450479	2.5470894
C	-0.3584678	3.6379783	-1.6128239
C	-1.5621347	4.3314823	0.3539454
C	0.5587951	1.6737138	4.2638680
H	0.0470175	0.4822596	2.5655991
C	0.7518102	4.0397605	3.8924717
H	0.4671697	4.7002079	1.8777814
C	-1.0024815	4.6236627	-2.3506133
H	0.3548504	2.9735529	-2.0858881
C	-2.2035601	5.3220464	-0.3861676
H	-1.8110223	4.2062519	1.4027349
C	0.8032516	2.9489819	4.7689407
H	0.5988481	0.8099749	4.9241631
H	0.9560588	5.0443664	4.2567840
C	-1.9279624	5.4858241	-1.7463810
H	-0.7809245	4.7244232	-3.4110845
H	-2.9398551	5.9587018	0.0999692
H	1.0349586	3.0953962	5.8210933
H	-2.4324356	6.2539015	-2.3266047
Co	-4.3790129	-0.0635843	0.3411630
C	-7.5051641	0.6674998	0.1199433
C	-4.2847441	2.5944358	-1.3636710
C	-6.4112216	-0.3554222	0.1414970
C	-3.6734907	1.7085816	-0.3269653
C	-5.9353422	-0.4786493	-2.4263887
C	-4.8237045	2.7091767	1.7976564
C	-5.7052254	-0.8664218	-0.9987746
C	-3.9204781	1.7506547	1.0889208
C	-5.9003658	-1.0232955	1.3087650
C	-2.6996638	0.6831449	-0.5670062
C	-4.7799347	-1.8774328	-0.5389918
C	-3.0925881	0.7489961	1.7082300
C	-6.3880991	-0.8305524	2.7110597
C	-2.1026005	0.3388028	-1.8880172
C	-4.9029231	-1.9774229	0.8824688
C	-2.2567991	0.1343419	0.6963697
C	-3.8721741	-2.7085111	-1.3902039
C	-3.0251381	0.4675523	3.1750544
C	-4.1257621	-2.9104872	1.7552392
C	-1.2312075	-0.8516352	0.8827775
H	-7.3724369	1.3773506	-0.7016745
H	-5.2719896	2.9498006	-1.0539858
H	-5.0108415	-0.5290851	-3.0084606
H	-5.2443387	2.2688203	2.7060752
H	-6.3314928	0.5370783	-2.5051019
H	-5.6482259	3.0307108	1.1556988
H	-7.5396575	1.2331303	1.0551636
H	-4.3913497	2.0730351	-2.3184439
H	-6.6994779	0.2027153	2.8895068
H	-1.1065150	0.7937168	-1.9528228
H	-5.6162308	-1.0852509	3.4415522
H	-2.7080227	0.7094442	-2.7191637
H	-3.7335176	-2.2679083	-2.3802210
H	-3.9705331	0.7007533	3.6726061
H	-8.4840566	0.1871940	-0.0125580
H	-3.6453185	3.4695482	-1.5280517
H	-6.6603408	-1.1560449	-2.8969768
H	-4.2634438	3.6057355	2.0926594
H	-2.8869115	-2.8234496	-0.9267801
H	-2.7803499	-0.5816152	3.3644184
H	-4.1104359	-2.5670728	2.7929183
H	-1.1065742	-1.5495977	0.0526558
H	-3.0896013	-2.9946474	1.4111549
H	-1.2477872	-1.3619761	1.8465705
H	-7.2551802	-1.4756595	2.9063204
H	-1.9778301	-0.7400297	-1.9981327
H	-4.2915383	-3.7135388	-1.5289536
H	-2.2377756	1.0761778	3.6350980
H	-4.5673826	-3.9155444	1.7421767
H	0.0139407	-0.2296686	0.7941259

TSADh$^{\bullet-}$ (H-abstraction from **Rco** with **Ah**$^{\bullet-}$)
110
Energy = -3495.270313862

B	3.1626648	-0.8633309	-0.3023400
C	4.5799770	-0.0659855	-0.4834110
C	3.4396779	-2.0690318	0.7660470
C	2.5815627	-1.4125905	-1.7254909
C	5.5363257	-0.4329840	-1.4464988

C	4.9337441	0.9890444	0.3781501
C	3.0697671	-1.9785429	2.1197434
C	4.1297514	-3.2372208	0.3907715
C	2.6052924	-0.5905169	-2.8710662
C	1.9215822	-2.6474589	-1.8675457
C	6.7737732	0.2083911	-1.5521326
H	5.2996165	-1.2397602	-2.1388258
C	6.1642502	1.6417987	0.2848007
H	4.2166874	1.3032978	1.1339779
C	3.3424103	-2.9963949	3.0376958
H	2.5609310	-1.0791087	2.4566436
C	4.4149332	-4.2620805	1.2944224
H	4.4503461	-3.3455721	-0.6445311
C	2.0013562	-0.9663428	-4.0729183
H	3.1050728	0.3728733	-2.8050517
C	1.3198378	-3.0437344	-3.0655883
H	1.8720369	-3.3163442	-1.0099183
C	7.0958545	1.2553089	-0.6843481
H	7.4869162	-0.1030023	-2.3145084
H	6.4010175	2.4571967	0.9672834
C	4.0150787	-4.1505385	2.6298356
H	3.0307779	-2.8896164	4.0761114
H	4.9485717	-5.1510403	0.9605654
C	1.3530907	-2.2002249	-4.1790101
H	2.0305715	-0.2947845	-4.9299798
H	0.8146522	-4.0067974	-3.1288425
H	8.0550767	1.7630647	-0.7634882
H	4.2294179	-4.9474155	3.3392732
H	0.8796646	-2.4975692	-5.1124510
N	2.1243947	0.1158104	0.3519129
N	1.7335646	1.1447418	-0.3446095
C	0.9852945	2.1124386	0.2383539
C	1.0490450	2.4156625	1.6777987
C	0.3302801	3.0256093	-0.6922142
C	1.1391617	1.4423253	2.6982160
C	1.2380549	3.7621799	2.0806941
C	0.6068511	2.9851130	-2.0828220
C	-0.6727248	3.9348787	-0.2754452
C	1.4087328	1.7955917	4.0231986
H	0.9612220	0.4053019	2.4633557
C	1.4892785	4.1139261	3.4007358
H	1.2202717	4.5403120	1.3234335
C	-0.0296348	3.8379114	-2.9751326
H	1.3352219	2.2601378	-2.4309575
C	-1.3089536	4.7877104	-1.1732582
H	-0.9608264	3.9556909	0.7708708
C	1.5807652	3.1274717	4.3933050
H	1.4706382	1.0109227	4.7750036
H	1.6348983	5.1614776	3.6570122
C	-0.9909499	4.7600315	-2.5349166
H	0.2202889	3.7835485	-4.0331383
H	-2.0769910	5.4671678	-0.8086856
H	1.7821147	3.3974413	5.4271187
H	-1.4921113	5.4205709	-3.2376123
Co	-3.2823048	-0.6263844	0.1066143
C	-6.4155711	-0.0996464	-0.4166419
C	-3.1800155	1.8013075	-1.9183033
C	-5.2818120	-1.0513093	-0.1863267
C	-2.6050814	1.0803180	-0.7425980
C	-4.6092749	-1.4756189	-2.6777725
C	-3.9359696	2.2840007	1.1602401
C	-4.4709852	-1.6656044	-1.1989761
C	-2.9492989	1.2886800	0.6375335
C	-4.8264482	-1.5324584	1.0903059
C	-1.5745329	0.0832414	-0.7902399
C	-3.5377207	-2.5584033	-0.5490057
C	-2.1234209	0.4164716	1.4302128
C	-5.4206249	-1.1833401	2.4198112
C	-0.8760164	-0.3891343	-2.0183267
C	-3.7593127	-2.4800445	0.8613001
C	-1.1968607	-0.2770298	0.5592977
C	-2.5270219	-3.4353519	-1.2193426
C	-2.1350381	0.3322480	2.9229762
C	-3.0039444	-3.2471098	1.8997196
C	-0.1400919	-1.1720121	0.9314501
H	-6.2539243	0.5035545	-1.3148831
H	-4.2008781	2.1408116	-1.7186388
H	-3.6410406	-1.5498249	-3.1810241
H	-4.3971622	1.9423855	2.0915312
H	-5.0414135	-0.4999340	-2.9155406
H	-4.7295221	2.4793361	0.4338368
H	-6.5420135	0.5809000	0.4301128
H	-3.1969260	1.1624110	-2.8052233
H	-5.7892433	-0.1534224	2.4361298
H	0.0921293	0.1184353	-2.0936200
H	-4.6908334	-1.2960412	3.2256093
H	-1.4517133	-0.1695991	-2.9211604
H	-2.3296332	-3.1097677	-2.2432525
H	-3.1195887	0.5756257	3.3322382
H	-7.3614047	-0.6427392	-0.5485936
H	-2.5683477	2.6814976	-2.1494669
H	-5.2664149	-2.2442476	-3.1065266
H	-3.4338510	3.2376216	1.3682026
H	-1.5765697	-3.4311423	-0.6756817
H	-1.8534960	-0.6684802	3.2637598
H	-3.0886373	-2.7772192	2.8832441
H	0.0881871	-1.9497394	0.2013315
H	-1.9404353	-3.3056453	1.6440487
H	-0.1889974	-1.5626138	1.9491423
H	-6.2697373	-1.8421161	2.6468315
H	-0.6614304	-1.4585671	-1.9850935
H	-2.8835712	-4.4731672	-1.2596978
H	-1.4067450	1.0378506	3.3405750
H	-3.3869986	-4.2725952	1.9851655
H	1.0432825	-0.4872484	0.8227648

TSBCf$^{\bullet-}$ (H-transfer from **Bf**$^{\bullet-}$ to **Rn**)
84
Energy = -3432.136986638

N	2.3162034	2.1867311	-0.4091510
N	3.1707596	1.4220379	-0.1181190
C	4.4212114	1.0210494	0.0320238
C	5.4836987	1.9567772	-0.3496107
C	4.6240765	-0.3366942	0.5643331
C	5.1996291	3.1073575	-1.1168928
C	6.8193702	1.7612782	0.0656139
C	3.7384541	-0.8553677	1.5263878
C	5.6597869	-1.1694394	0.1032824
C	6.2059268	4.0020708	-1.4698492
H	4.1785538	3.2980001	-1.4362624
C	7.8199913	2.6619003	-0.2897986
H	7.0666130	0.9069579	0.6868609
C	3.8716580	-2.1574625	1.9981658
H	2.9405771	-0.2197983	1.8965436
C	5.7981323	-2.4696645	0.5850159
H	6.3390514	-0.8069678	-0.6621869
C	7.5264440	3.7873248	-1.0647495
H	5.9559905	4.8761713	-2.0660797
H	8.8372779	2.4865062	0.0515593
C	4.9062889	-2.9739664	1.5345231
H	3.1731114	-2.5328138	2.7412497
H	6.5988509	-3.0981781	0.2034342
H	8.3095782	4.4878520	-1.3402487
H	5.0146194	-3.9893617	1.9053751

B	-1.3547200	-0.6925566	-0.1613717
C	-3.0027719	-0.6848950	-0.2828701
C	-0.6033637	-1.7236587	-1.2101151
C	-0.8052249	-1.1880330	1.3149674
C	-3.9198513	-0.7992669	0.7595487
C	-3.5759196	-0.3439933	-1.5105778
C	0.7937111	-1.7286768	-1.2657872
C	-1.2018344	-2.7407962	-1.9566987
C	0.0293508	-0.4809879	2.1804071
C	-1.1008628	-2.4860388	1.7383170
C	-5.2949225	-0.6235391	0.6014073
F	-3.5205600	-1.0638209	2.0295415
C	-4.9363153	-0.1592662	-1.7185799
F	-2.7764204	-0.1571771	-2.5932530
C	1.5549545	-2.6195635	-2.0144846
F	1.4830340	-0.8096846	-0.5524904
C	-0.4835494	-3.6588467	-2.7223074
F	-2.5449902	-2.9190134	-1.9641627
C	0.5080826	-1.0052783	3.3817613
F	0.4571961	0.7702506	1.8936554
C	-0.6415174	-3.0527358	2.9199652
F	-1.9038597	-3.2710078	0.9715296
C	-5.8113755	-0.3055030	-0.6469137
F	-6.1306070	-0.7474050	1.6575525
F	-5.4198837	0.1581601	-2.9403206
C	0.9037582	-3.5984529	-2.7577132
F	2.8996761	-2.5466820	-2.0289813
F	-1.1257198	-4.6182951	-3.4239649
C	0.1757052	-2.2997104	3.7563976
F	1.3188025	-0.2708336	4.1764781
F	-0.9798027	-4.3142331	3.2684240
F	-7.1377897	-0.1336208	-0.8178490
F	1.6074672	-4.4794219	-3.4940792
F	0.6467776	-2.8231490	4.9064726
N	-1.0202452	0.8211651	-0.4407567
N	-1.6757708	1.6898303	0.3302420
C	-1.4464615	2.9501890	-0.0838614
C	-0.6190917	2.9416197	-1.2325720
C	-2.0923283	4.0630138	0.6142658
C	-0.2021829	1.5324603	-1.3831484
C	-0.2352128	3.8845811	-2.1952524
C	-3.2599265	3.8388270	1.3672589
C	-1.5696696	5.3658756	0.5602609
C	0.1386884	1.0906774	-2.7381623
C	0.3347332	3.4408724	-3.3804052
H	-0.4538172	4.9387730	-2.0601349
C	-3.8867567	4.8868518	2.0340701
H	-3.6592720	2.8306135	1.4181258
C	-2.2030607	6.4153892	1.2255025
H	-0.6481170	5.5528357	0.0183743
C	0.4727467	2.0484781	-3.6553040
H	0.2084897	0.0363999	-2.9701626
H	0.6120658	4.1592015	-4.1462433
C	-3.3655153	6.1830536	1.9628250
H	-4.7894346	4.6961326	2.6087369
H	-1.7807664	7.4155291	1.1750244
H	0.8063600	1.7428220	-4.6435288
H	-3.8587193	7.0010823	2.4805871
H	0.9163948	1.6202183	-0.8490650

TSBCh1$^{\cdot-}$ (higher TS: H-transfer from **Bh**$^{\cdot-}$)
84
Energy = -1942.866836030

N	1.2288018	1.7183178	0.3925768
N	2.2157945	1.0845282	0.5043402
C	3.5176729	0.9148602	0.6834704
C	4.3314683	2.1165447	0.8833906
C	4.0253604	-0.4648589	0.6489347
C	3.8235766	3.4008669	0.5890506
C	5.6397296	2.0377505	1.4114144
C	3.2574655	-1.5214738	1.1732231
C	5.2637768	-0.7773584	0.0555573
C	4.5945241	4.5426046	0.7899106
H	2.8138982	3.4994236	0.1992495
C	6.4042188	3.1841506	1.6099086
H	6.0488849	1.0698151	1.6812050
C	3.7033784	-2.8379006	1.0909332
H	2.3023036	-1.3068666	1.6454056
C	5.7123757	-2.0945381	-0.0128198
H	5.8623679	0.0164233	-0.3813439
C	5.8937765	4.4474239	1.2968419
H	4.1762414	5.5164716	0.5473609
H	7.4055231	3.0891498	2.0231905
C	4.9346903	-3.1351482	0.5027544
H	3.0806480	-3.6314606	1.4930255
H	6.6667631	-2.3104949	-0.4869347
H	6.4929847	5.3399631	1.4529464
H	5.2825621	-4.1628446	0.4433167
B	-1.4507780	-1.6493799	-0.3978173
C	-2.8927511	-2.4062882	-0.5283145
C	-0.4238204	-2.1379333	-1.5745003
C	-0.7601401	-1.8724858	1.0659402
C	-3.2080207	-3.5686153	0.1934708
C	-3.8726288	-1.9550339	-1.4339493
C	0.9645417	-1.9827398	-1.4162316
C	-0.8580498	-2.6639130	-2.8028499
C	-0.7218650	-0.8998932	2.0783659
C	-0.1543219	-3.1086294	1.3699775
C	-4.4197635	-4.2463891	0.0266622
H	-2.4924636	-3.9503674	0.9184966
C	-5.0873190	-2.6183930	-1.6128846
H	-3.6821744	-1.0511377	-2.0088178
C	1.8737323	-2.3014365	-2.4250955
H	1.3449139	-1.5887796	-0.4798803
C	0.0373001	-2.9861265	-3.8274242
H	-1.9233231	-2.8146396	-2.9656250
C	-0.1197626	-1.1387659	3.3174829
H	-1.1707704	0.0727454	1.9002838
C	0.4284175	-3.3715150	2.6102365
H	-0.1243300	-3.8804689	0.6031706
C	-5.3697333	-3.7741803	-0.8804975
H	-4.6250350	-5.1413007	0.6117116
H	-5.8177319	-2.2319699	-2.3215216
C	1.4104206	-2.7995141	-3.6454693
H	2.9384303	-2.1553037	-2.2562681
H	-0.3351644	-3.3819790	-4.7704720
C	0.4539270	-2.3805383	3.5948518
H	-0.0958634	-0.3503047	4.0672137
H	0.8782578	-4.3433029	2.8049171
H	-6.3162779	-4.2938783	-1.0110216
H	2.1086567	-3.0471175	-4.4419103
H	0.9240264	-2.5701605	4.5569204
N	-1.7448887	-0.0959476	-0.6121383
N	-2.7039658	0.4635813	0.1029604
C	-2.7268519	1.8070630	-0.0873832
C	-1.7248275	2.1826601	-1.0060522
C	-3.7518110	2.5993193	0.5891496
C	-0.9521090	0.9404688	-1.2554680
C	-1.4387741	3.3453609	-1.7394099
C	-4.9231286	1.9762245	1.0625156
C	-3.6026750	3.9823852	0.7945037
C	-0.3200614	0.8371906	-2.5863795
C	-0.6074699	3.2551976	-2.8451790
H	-1.9305592	4.2846864	-1.5080301

C	-5.9116900	2.7148626	1.7053710
H	-5.0359093	0.9067153	0.9131048
C	-4.5972091	4.7200993	1.4359322
H	-2.6911138	4.4774424	0.4759522
C	-0.1027264	1.9921593	-3.2821920
H	0.0604056	-0.1142253	-2.9322530
H	-0.4020494	4.1409683	-3.4396445
C	-5.7584627	4.0931109	1.8920955
H	-6.8101500	2.2161328	2.0602677
H	-4.4593659	5.7876628	1.5874766
H	0.4407494	1.9467425	-4.2230268
H	-6.5332365	4.6690040	2.3908553
H	-0.0265191	1.0671792	-0.5149931

TSBCh$^{\cdot-}$ (H-transfer from **Bh**$^{\cdot-}$ to **Rn**)
84
Energy = -1942.871994621

N	1.2384730	1.4965230	0.3091840
N	0.9720890	2.5000630	0.8753690
C	1.0593830	3.3470140	1.8922390
C	1.8912260	2.9569080	3.0337980
C	0.3085210	4.6053610	1.7727370
C	2.3522560	1.6307240	3.1782330
C	2.2828950	3.8956490	4.0143040
C	0.2167580	5.2601340	0.5298340
C	-0.3627280	5.1770300	2.8692380
C	3.1373050	1.2572330	4.2640370
H	2.0733390	0.8824750	2.4429330
C	3.0726790	3.5146580	5.0962160
H	1.9740970	4.9313770	3.9174910
C	-0.5212530	6.4331480	0.3877540
H	0.7242210	4.8331140	-0.3301020
C	-1.0911510	6.3557490	2.7280600
H	-0.3406850	4.6719740	3.8302420
C	3.5019610	2.1919590	5.2374030
H	3.4584730	0.2223800	4.3532010
H	3.3594870	4.2613560	5.8325940
C	-1.1744990	6.9948280	1.4883620
H	-0.5777840	6.9154440	-0.5852500
H	-1.6155640	6.7656250	3.5880510
H	4.1133920	1.8967600	6.0856080
H	-1.7477230	7.9112210	1.3794540
B	-1.3714840	-1.5776210	-0.3875350
C	-2.7753200	-2.4113380	-0.4527150
C	-0.3658600	-2.0115270	-1.6032750
C	-0.6168240	-1.7603540	1.0500120
C	-2.9975790	-3.5868850	0.2826280
C	-3.8181340	-2.0110570	-1.3107610
C	1.0165180	-1.7619320	-1.5147750
C	-0.8265530	-2.5734950	-2.8055810
C	-0.6026840	-0.7997360	2.0739360
C	0.0701010	-2.9617280	1.3184090
C	-4.1797220	-4.3251670	0.1713300
H	-2.2311210	-3.9301520	0.9739180
C	-5.0047090	-2.7355570	-1.4340130
H	-3.7000440	-1.0983470	-1.8913940
C	1.8860560	-2.0218360	-2.5755890
H	1.4148260	-1.3319390	-0.5987940
C	0.0312050	-2.8418050	-3.8764160
H	-1.8867730	-2.7944700	-2.9103530
C	0.0430570	-1.0212210	3.2936440
H	-1.1138630	0.1467130	1.9249490
C	0.7141390	-3.2013950	2.5330960
H	0.1165030	-3.7239230	0.5423990
C	-5.1934130	-3.9032550	-0.6907430
H	-4.3121610	-5.2282200	0.7648260
H	-5.7864140	-2.3874340	-2.1069210
C	1.3947140	-2.5583440	-3.7687060
H	2.9466540	-1.7995790	-2.4759640
H	-0.3632410	-3.2686190	-4.7966290
C	0.7037060	-2.2261490	3.5336310
H	0.0404130	-0.2436270	4.0540380
H	1.2321980	-4.1447190	2.6969240
H	-6.1172490	-4.4707800	-0.7777070
H	2.0660940	-2.7595730	-4.6003350
H	1.2124650	-2.3986560	4.4794170
N	-1.7472570	-0.0431170	-0.6125620
N	-2.6392100	0.5090510	0.1914350
C	-2.6596670	1.8564730	0.0344020
C	-1.7333630	2.2425670	-0.9558760
C	-3.6089660	2.6496070	0.8123310
C	-1.0137150	0.9995280	-1.3188500
C	-1.5245240	3.4190400	-1.6970110
C	-4.7663800	2.0425020	1.3366630
C	-3.3941570	4.0149470	1.0654330
C	-0.5496450	0.9167290	-2.7199850
C	-0.8353180	3.3432720	-2.8941480
H	-1.9737640	4.3557170	-1.3860150
C	-5.6826560	2.7821640	2.0781320
H	-4.9269300	0.9849810	1.1492780
C	-4.3168020	4.7530010	1.8046730
H	-2.4846940	4.4929720	0.7176700
C	-0.4046010	2.0815570	-3.4154780
H	-0.2286870	-0.0332640	-3.1263160
H	-0.6936070	4.2382740	-3.4935040
C	-5.4666130	4.1447920	2.3120450
H	-6.5719290	2.2972550	2.4731760
H	-4.1250140	5.8049830	1.9962910
H	0.0181940	2.0501850	-4.4169380
H	-6.1844570	4.7225690	2.8881970
H	-0.0001660	1.0527340	-0.6963190

TSDEf (trapping **Dco** with $B(C_6F_5)_3$)
84
Energy = -4372.550279455

Co	-0.0389928	-0.6560644	-3.9906661
C	0.6340574	0.3764024	-6.9725306
C	0.9854316	2.2967206	-3.6095956
C	0.4973317	-0.7424025	-5.9856459
C	0.1938160	1.1270566	-3.1155243
C	2.9549258	-0.7664048	-5.1039403
C	-2.1013655	1.6913216	-4.2483113
C	1.5395020	-1.2539855	-5.1377030
C	-1.1954536	0.8603934	-3.3932830
C	-0.6871657	-1.5128174	-5.7350703
C	0.6588198	0.1044743	-2.2176165
C	1.0094570	-2.3782950	-4.3958754
C	-1.5636302	-0.3187341	-2.6563137
C	-2.0069542	-1.3468354	-6.4228629
C	2.0298038	0.0160668	-1.6280241
C	-0.3622878	-2.5322930	-4.7597785
C	-0.4728164	-0.6999249	-1.7557200
C	1.7228481	-3.2218775	-3.3874434
C	-2.9290179	-0.9262522	-2.6140338
C	-1.2758169	-3.5832261	-4.2122363
C	-0.4720584	-1.6911297	-0.8088760
H	1.3930700	1.0967501	-6.6544704
H	0.6009033	2.6638261	-4.5650260
H	3.4365160	-1.0000340	-4.1510677
H	-2.9244507	1.0948315	-4.6505716
H	3.0103189	0.3153124	-5.2571876
H	-1.5598997	2.1361131	-5.0882458
H	-0.3106113	0.9129498	-7.1011558
H	2.0405374	2.0402471	-3.7407284

H	-2.1737450	-0.3081628	-6.7214369
H	2.0433898	0.5181101	-0.6518874
H	-2.8357282	-1.6530700	-5.7784504
H	2.7790922	0.4974512	-2.2612413
H	2.7166663	-2.8253308	-3.1650398
H	-3.4753914	-0.7690678	-3.5477897
H	0.9333309	-0.0054854	-7.9580628
H	0.9326551	3.1224217	-2.8878312
H	3.5442556	-1.2426393	-5.8988107
H	-2.5388902	2.5108831	-3.6643581
H	1.1522211	-3.2642450	-2.4492942
H	-2.8790346	-2.0004360	-2.4172614
H	-1.0508422	-4.5607495	-4.6584553
H	0.4238103	-1.9530236	-0.2597423
H	-2.3235430	-3.3549989	-4.4220176
H	-1.3752025	-2.2559443	-0.6029654
H	-2.0497165	-1.9635361	-7.3306496
H	2.3257457	-1.0243201	-1.4645699
H	1.8459224	-4.2495069	-3.7518110
H	-3.5235327	-0.4770778	-1.8057826
H	-1.1529790	-3.6771591	-3.1267795
B	0.0563805	0.4385979	2.5298123
C	1.5133207	1.0471117	2.5022001
C	-0.1570877	-0.6326450	3.6742324
C	-1.0772293	0.9533745	1.6088299
C	1.7828036	2.4032765	2.7019310
C	2.6306073	0.2228548	2.3467499
C	0.0730758	-0.2610214	5.0024595
C	-0.5147052	-1.9680792	3.4770759
C	-2.4291083	0.5820321	1.7803899
C	-0.8544082	1.8642435	0.5516529
C	3.0712713	2.9213696	2.7355862
C	3.9354967	0.7041892	2.3496456
C	-0.0592891	-1.1366856	6.0745093
C	-0.6353484	-2.8810019	4.5184571
C	-3.4568545	1.0364930	0.9684989
C	-1.8626613	2.3571377	-0.2605126
C	4.1552601	2.0646678	2.5500902
C	-0.4133042	-2.4600469	5.8276513
C	-3.1738634	1.9311131	-0.0616446
F	0.7603995	3.2643644	2.9012162
F	2.4587882	-1.1037619	2.1444388
F	0.4023047	1.0218379	5.2894909
F	-0.7138266	-2.4265147	2.2265809
F	-2.7861835	-0.2681629	2.7628752
F	0.3881187	2.2996331	0.2685165
F	3.2862273	4.2322933	2.9451809
F	4.9792332	-0.1225182	2.1618045
F	0.1439768	-0.7206499	7.3373389
F	-0.9608597	-4.1638452	4.2767081
F	-4.7235672	0.6299184	1.1641572
F	-1.5922027	3.2451072	-1.2317795
F	5.4051313	2.5491750	2.5692077
F	-0.5397564	-3.3249995	6.8444657
F	-4.1555104	2.3857656	-0.8492675

4. References

[1] (a) C. Tang, Q. Liang, A. R. Jupp, T. C. Johnstone, R. C. Neu, D. Song, S. Grimme, D. W. Stephan, *Angewandte Chemie International Edition* **2017**, *56*, 16588-16592; (b) J. Zhou, L. L. Liu, L. L. Cao, D. W. Stephan, *Chem. Commun.* **2018**, *54*, 4390-4393.

[2] TURBOMOLE, version 7.3, **2018**, TURBOMOLE GmbH, Karlsruhe, See http://www.turbomole.com

[3] J. Tao, J. P. Perdew, V. N. Staroverov, G. E. Scuseria, *Physical Review Letters* **2003**, *91*, 146401.

[4] (a) S. Grimme, J. Antony, S. Ehrlich, H. Krieg, *The Journal of Chemical Physics* **2010**, *132*, 154104-154119; (b) S. Grimme, S. Ehrlich, L. Goerigk, *Journal of Computational Chemistry* **2011**, *32*, 1456-1465.

[5] (a) F. Weigend, M. Häser, H. Patzelt, R. Ahlrichs, *Chemical Physics Letters* **1998**, *294*, 143-152; (b) F. Weigend, R. Ahlrichs, *Physical Chemistry Chemical Physics* **2005**, *7*, 3297-3305.

[6] A. Klamt, G. Schüürmann, *Journal of the Chemical Society, Perkin Transactions 2* **1993**, 799-805.

[7] (a) K. Eichkorn, F. Weigend, O. Treutler, R. Ahlrichs, *Theoretical Chemistry Accounts* **1997**, *97*, 119-124; (b) F. Weigend, *Physical Chemistry Chemical Physics* **2006**, *8*, 1057-1065.

[8] P. Deglmann, K. May, F. Furche, R. Ahlrichs, *Chemical Physics Letters* **2004**, *384*, 103-107.

[9] S. Grimme, *Chemistry - A European Journal* **2012**, *18*, 9955-9964.

[10] F. Eckert, A. Klamt, *AIChE Journal* **2002**, *48*, 369-385.

[11] F. Eckert, A. Klamt, COSMOtherm, Version C3.0, Release 16.01, **2015**, COSMOlogic GmbH & Co., Leverkusen, Germany

[12] Y. Zhao, D. G. Truhlar, *The Journal of Physical Chemistry A* **2005**, *109*, 5656-5667.

[13] F. Weigend, F. Furche, R. Ahlrichs, *The Journal of Chemical Physics* **2003**, *119*, 12753-12762.

[14] J. L. Robbins, N. Edelstein, B. Spencer, J. C. Smart, *Journal of the American Chemical Society* **1982**, *104*, 1882-1893.
